# Supplementary material for: PSTPIP2 ameliorates aristolochic acid nephropathy by suppressing interleukin-19-mediated neutrophil extracellular trap formation
Source: eLife. 2024 Feb 5;13:e89740. doi: 10.7554/eLife.89740 (PMC10906995; doi:10.7554/eLife.89740)
Supplement: Figure 1—source data 2. [file elife-89740-fig1-data2.zip › Figure 1-data 2/Figure 1—source data 2.pptx]

## Slide 1
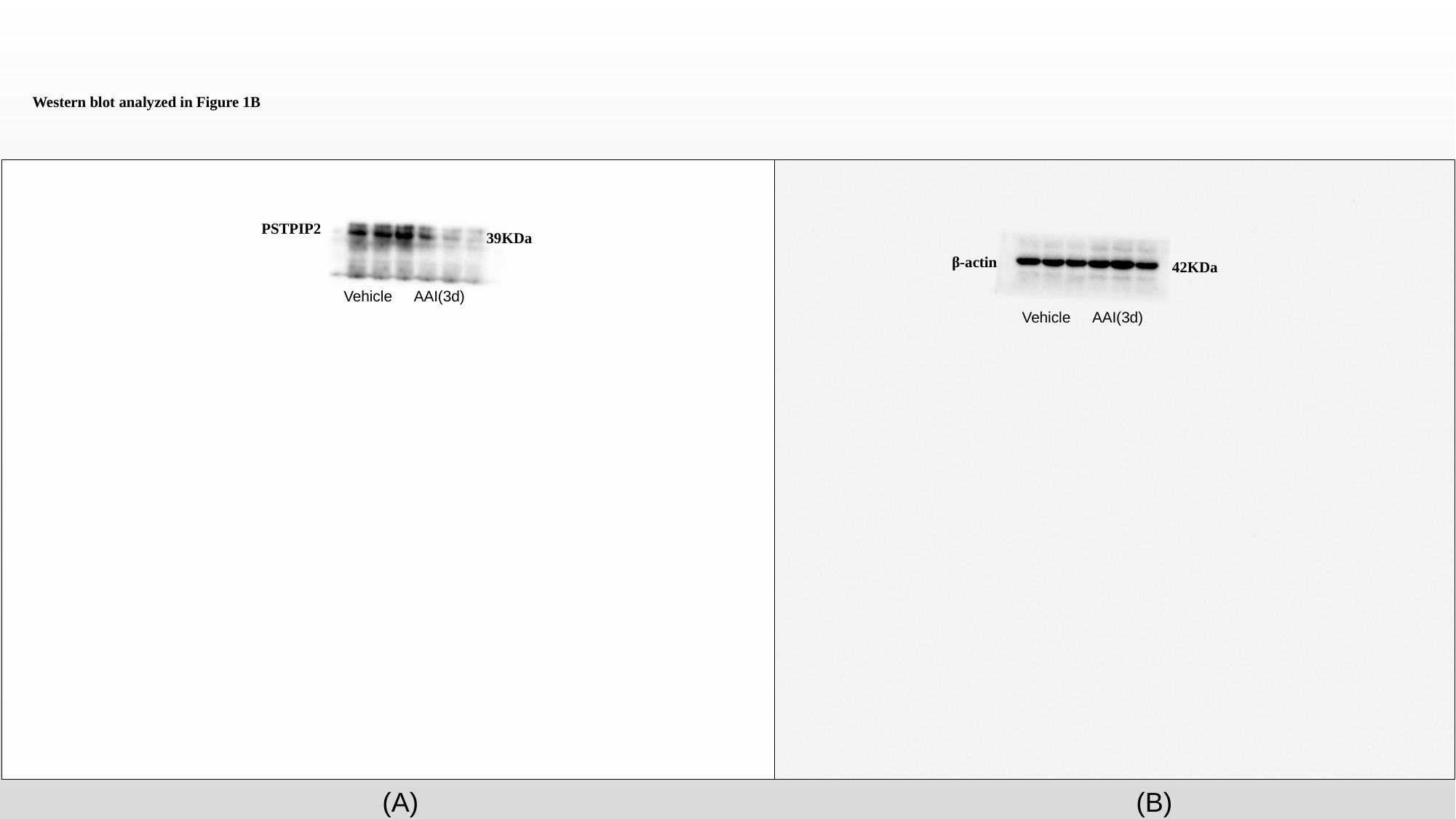

Western blot analyzed in Figure 1B
PSTPIP2
39KDa
β-actin
42KDa
Vehicle
AAI(3d)
Vehicle
AAI(3d)
(A)
(B)

## Slide 2
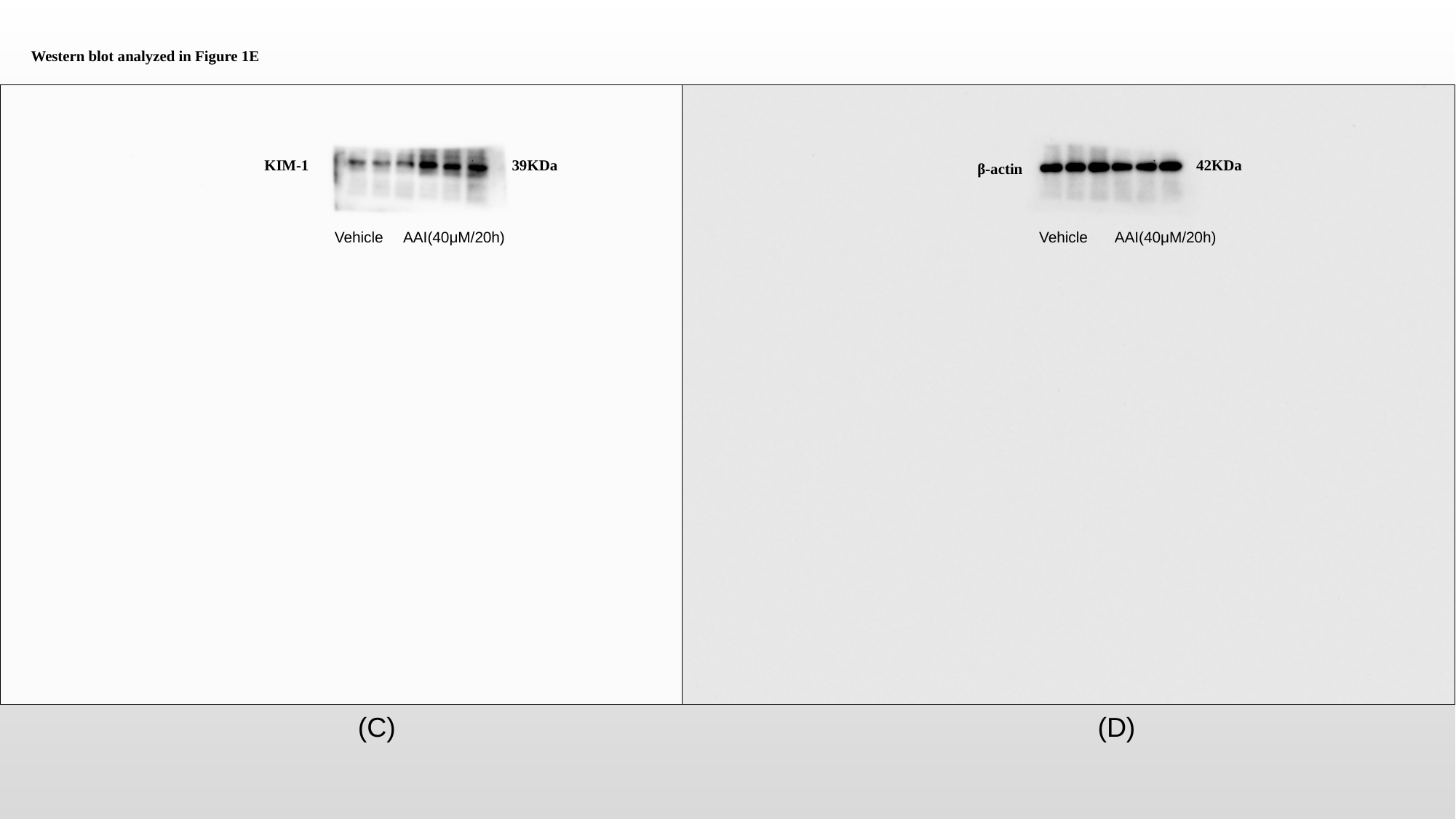

Western blot analyzed in Figure 1E
39KDa
42KDa
KIM-1
β-actin
Vehicle
AAI(40μM/20h)
Vehicle
AAI(40μM/20h)
(C)
(D)

## Slide 3
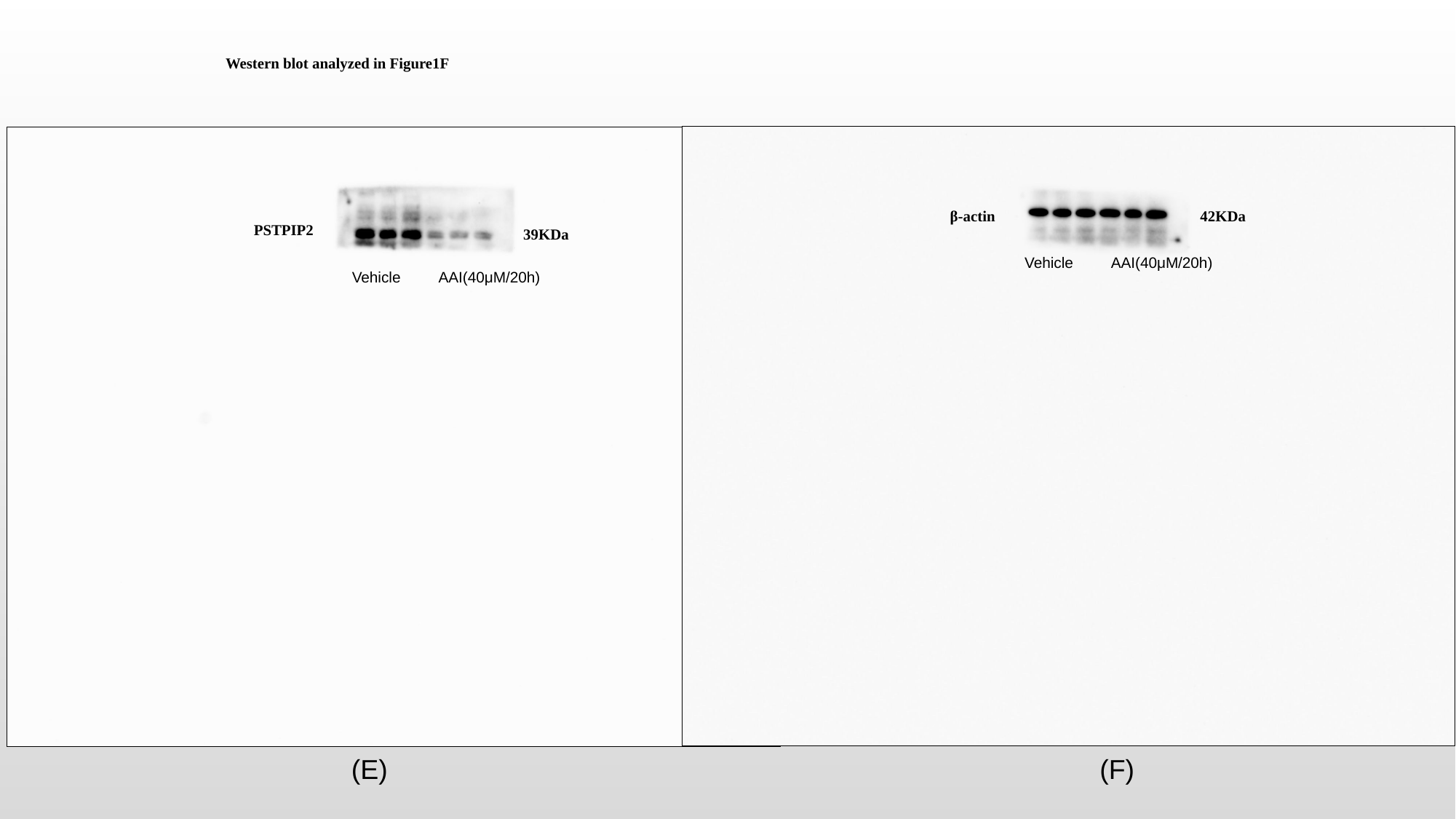

Western blot analyzed in Figure1F
42KDa
β-actin
PSTPIP2
39KDa
Vehicle
AAI(40μM/20h)
Vehicle
AAI(40μM/20h)
(E)
(F)
