# Supplementary figures and images for: PSTPIP2 ameliorates aristolochic acid nephropathy by suppressing interleukin-19-mediated neutrophil extracellular trap formation
Source: eLife. 2024 Feb 5;13:e89740. doi: 10.7554/eLife.89740 (PMC10906995; doi:10.7554/eLife.89740)

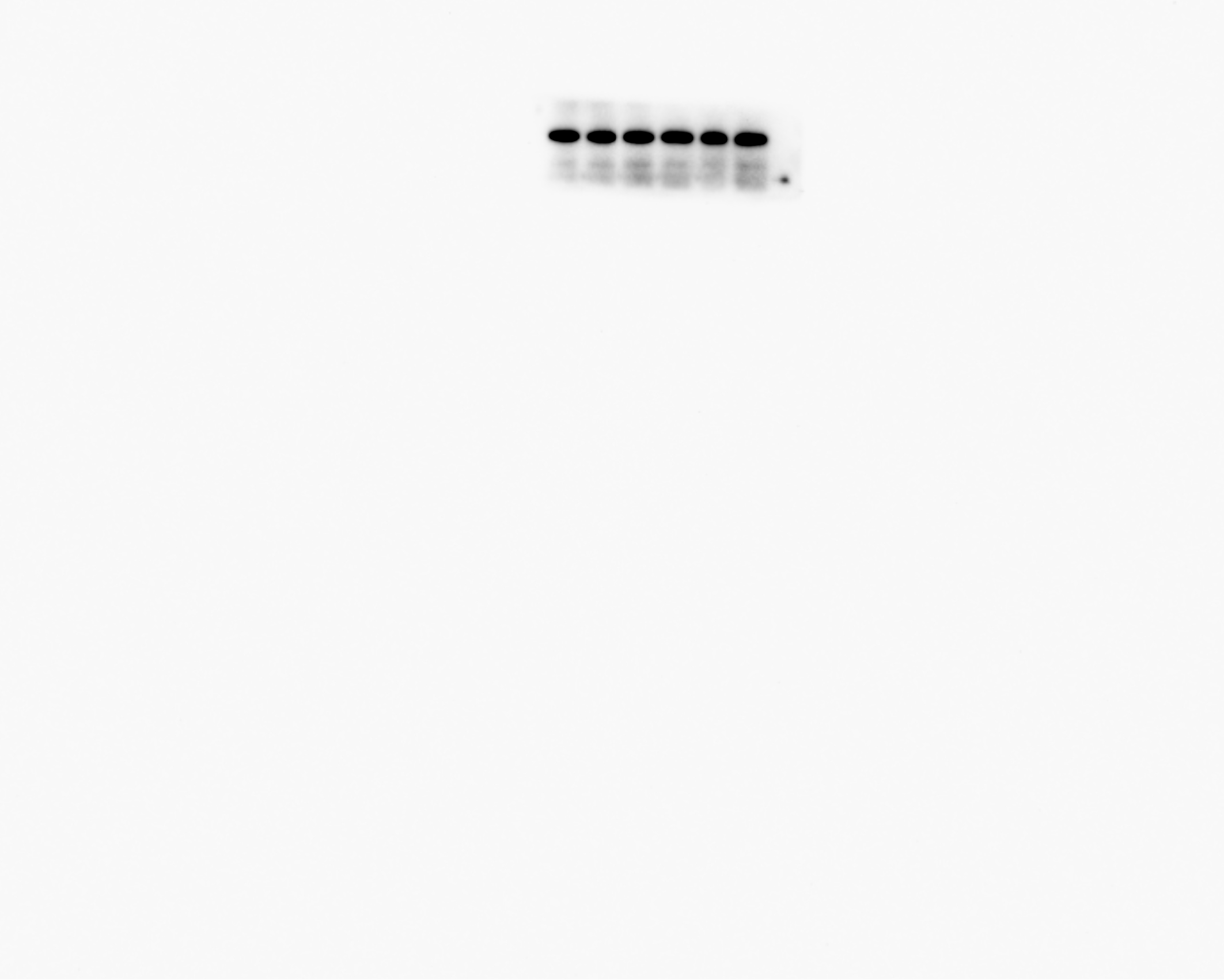

Supplement: Figure 1—source data 2. [file elife-89740-fig1-data2.zip › Figure 1-data 2/Figure 1— data 2-(F).jpg]

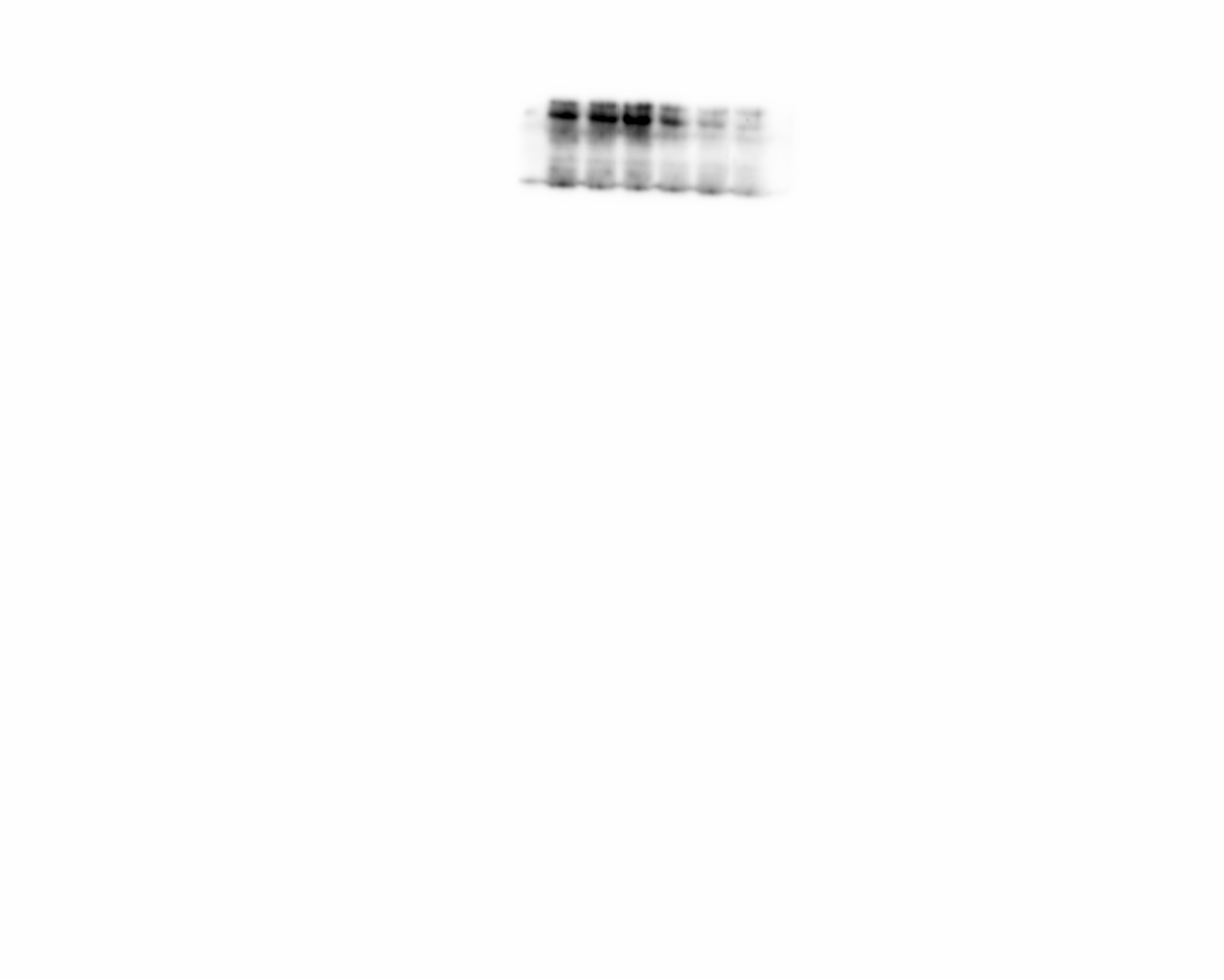

Supplement: Figure 1—source data 2. [file elife-89740-fig1-data2.zip › Figure 1-data 2/Figure 1—data 2-(A).jpg]

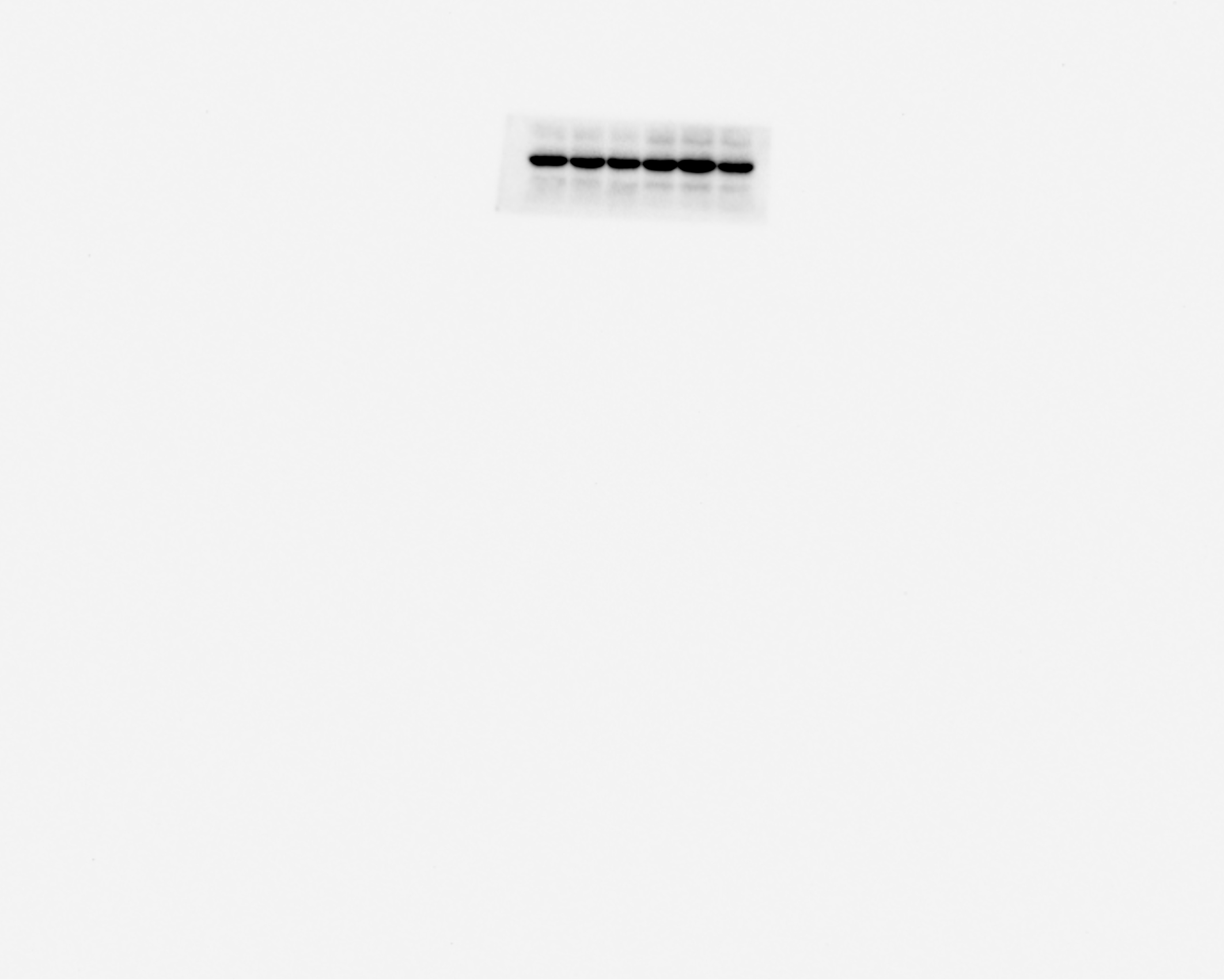

Supplement: Figure 1—source data 2. [file elife-89740-fig1-data2.zip › Figure 1-data 2/Figure 1—data 2-(B).jpg]

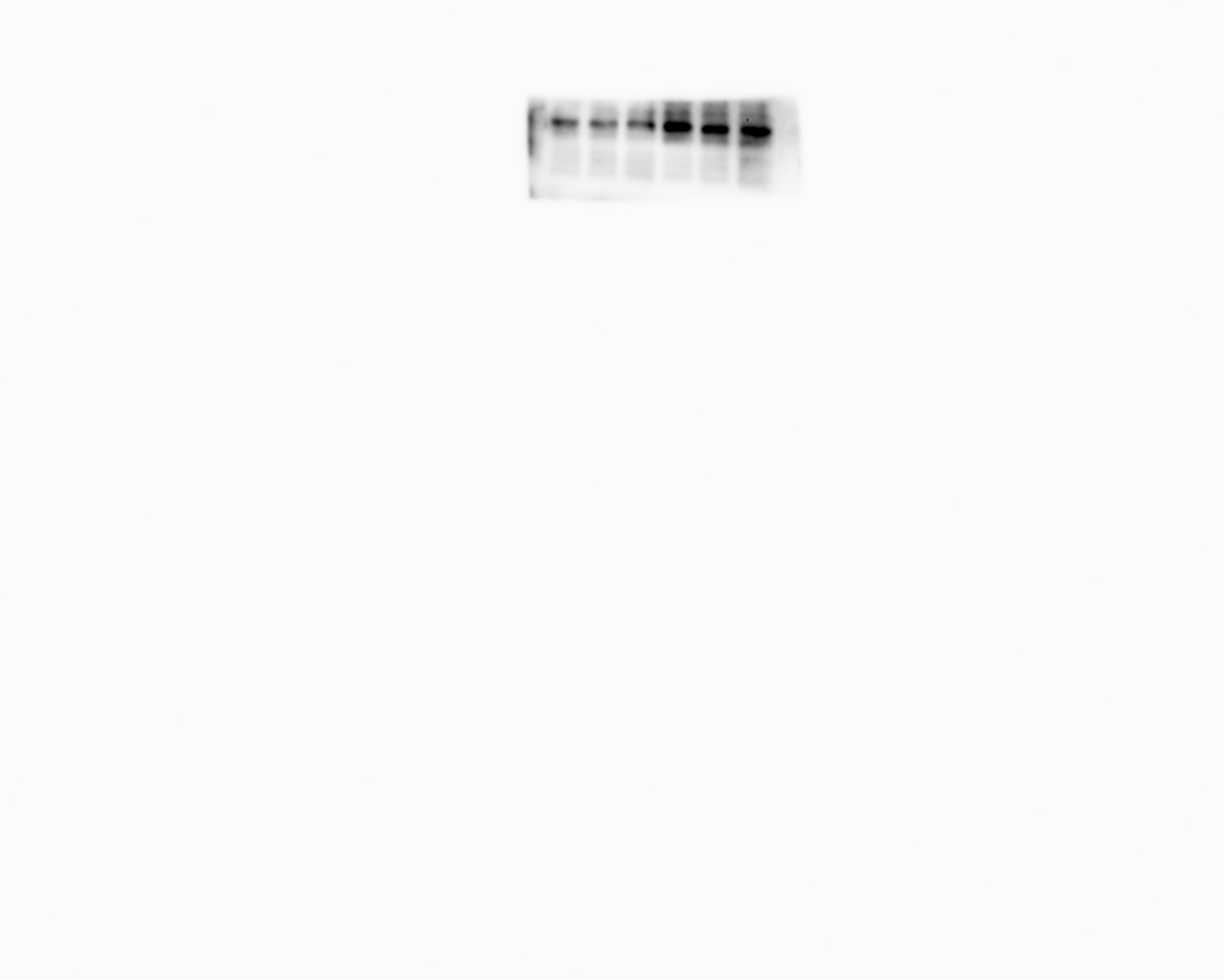

Supplement: Figure 1—source data 2. [file elife-89740-fig1-data2.zip › Figure 1-data 2/Figure 1—data 2-(C).jpg]

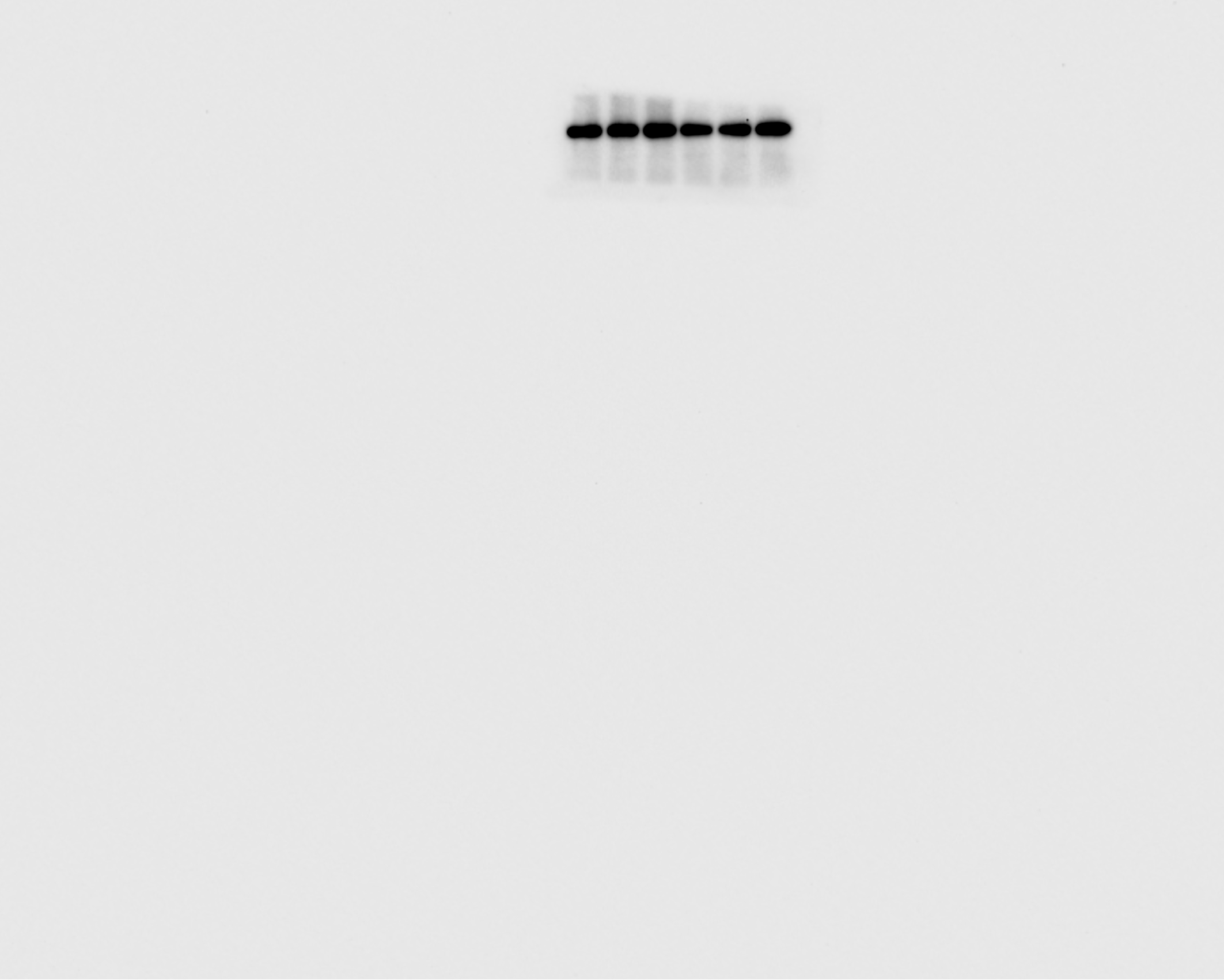

Supplement: Figure 1—source data 2. [file elife-89740-fig1-data2.zip › Figure 1-data 2/Figure 1—data 2-(D).jpg]

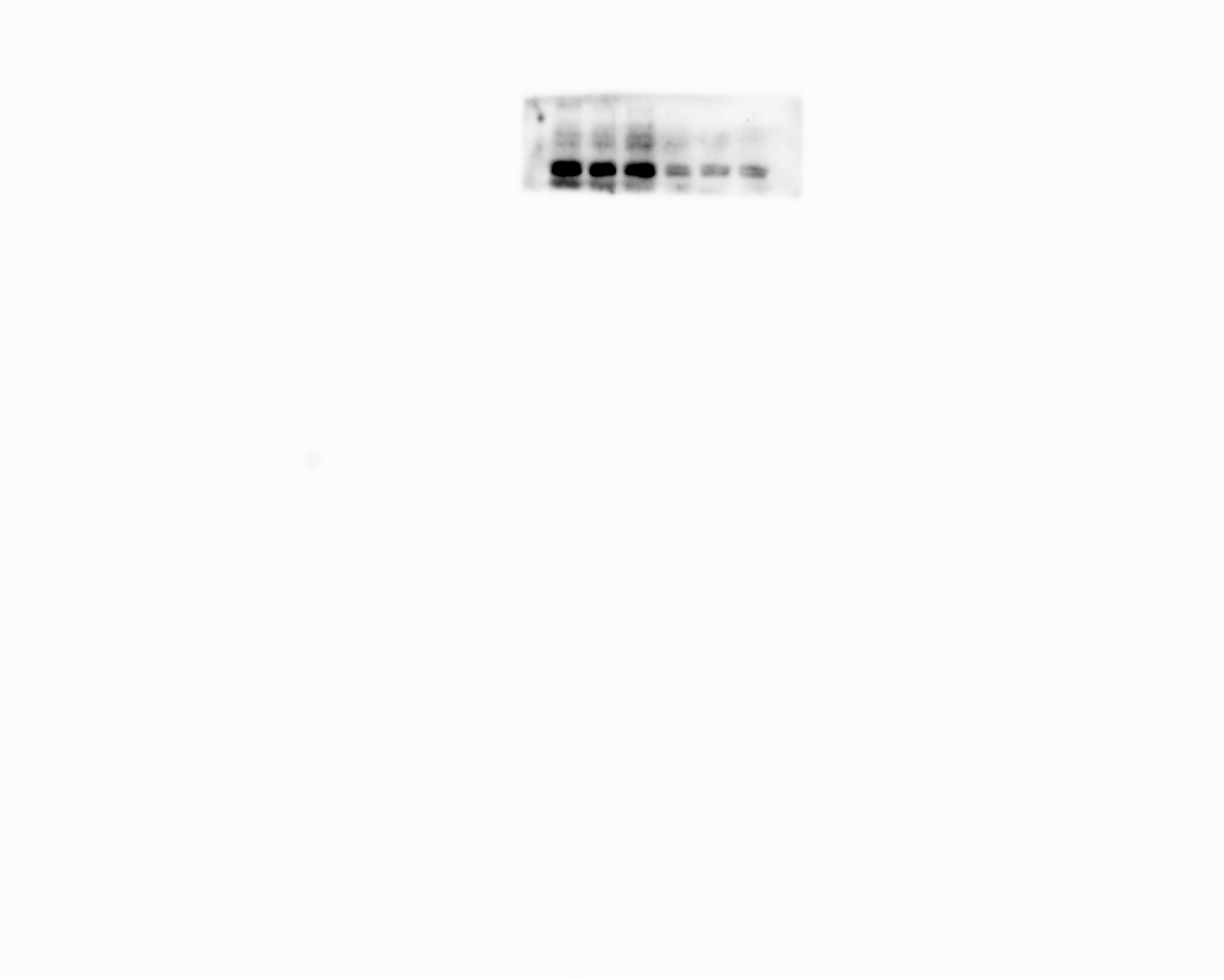

Supplement: Figure 1—source data 2. [file elife-89740-fig1-data2.zip › Figure 1-data 2/Figure 1—data 2-(E).jpg]

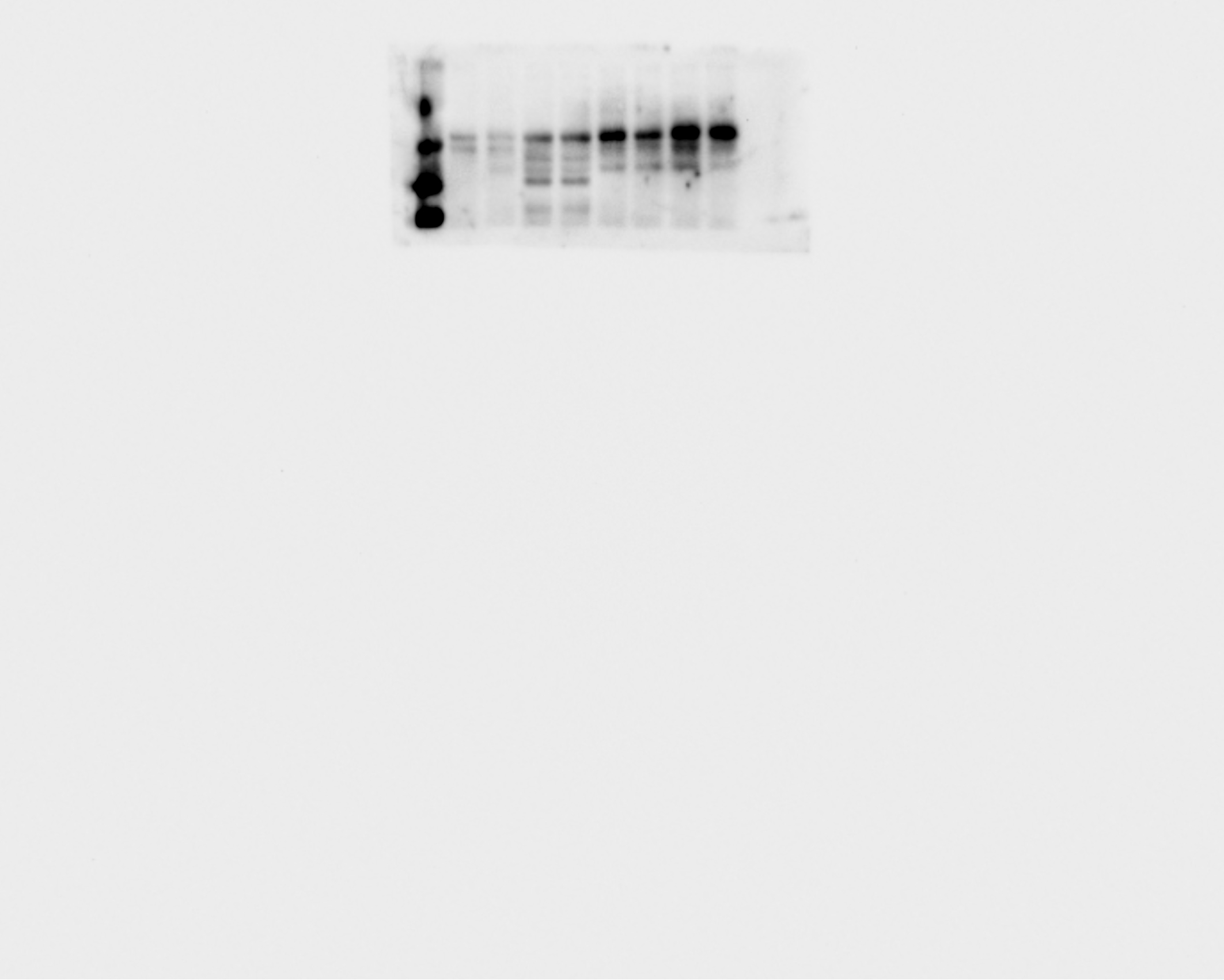

Supplement: Figure 1—figure supplement 1—source data 2. [file elife-89740-fig1-figsupp1-data2.zip › Figure 1-figure supplement 1-data 2/Figure 1-figure supplement 1—data 2-(A).jpg]

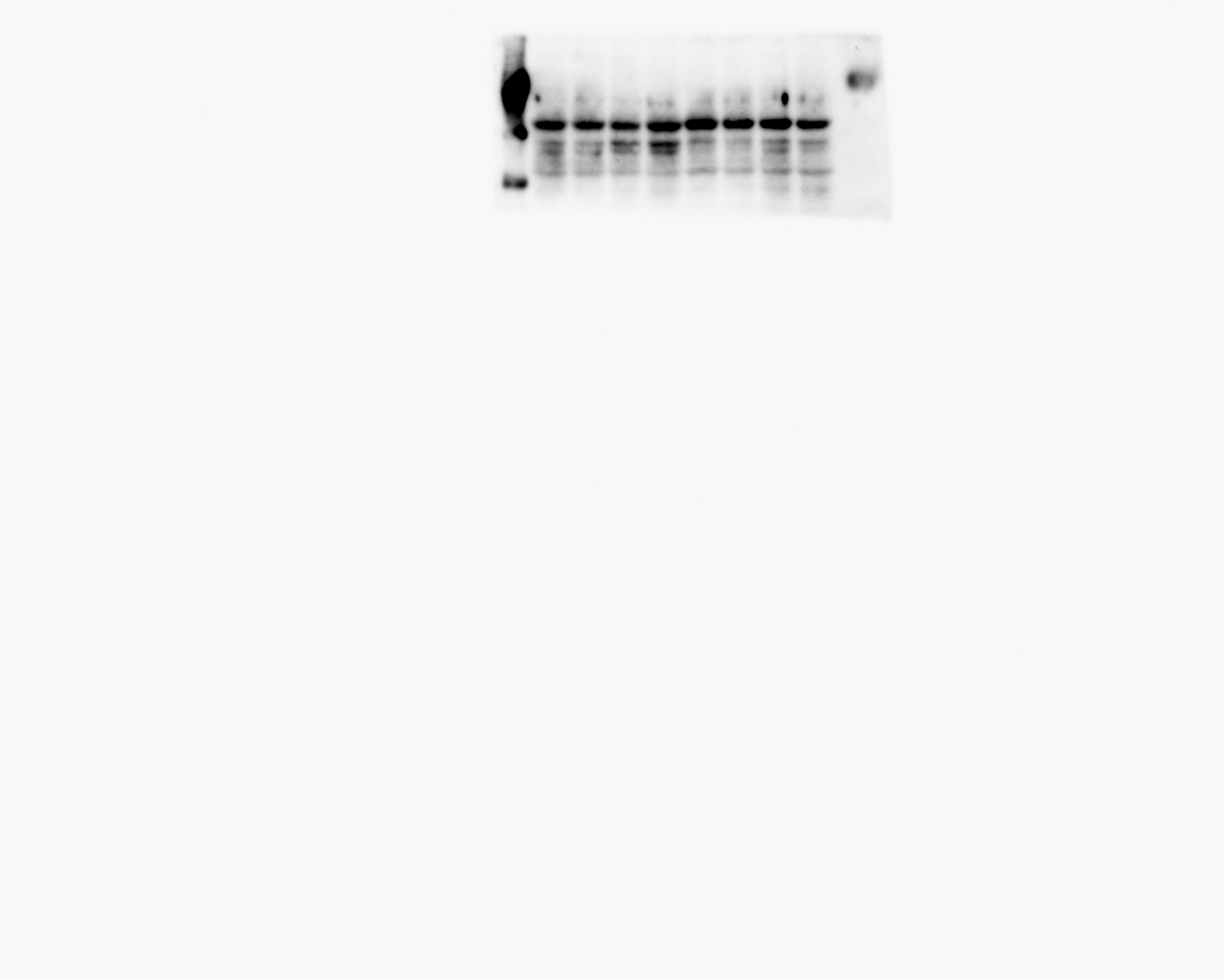

Supplement: Figure 1—figure supplement 1—source data 2. [file elife-89740-fig1-figsupp1-data2.zip › Figure 1-figure supplement 1-data 2/Figure 1-figure supplement 1—data 2-(B).jpg]

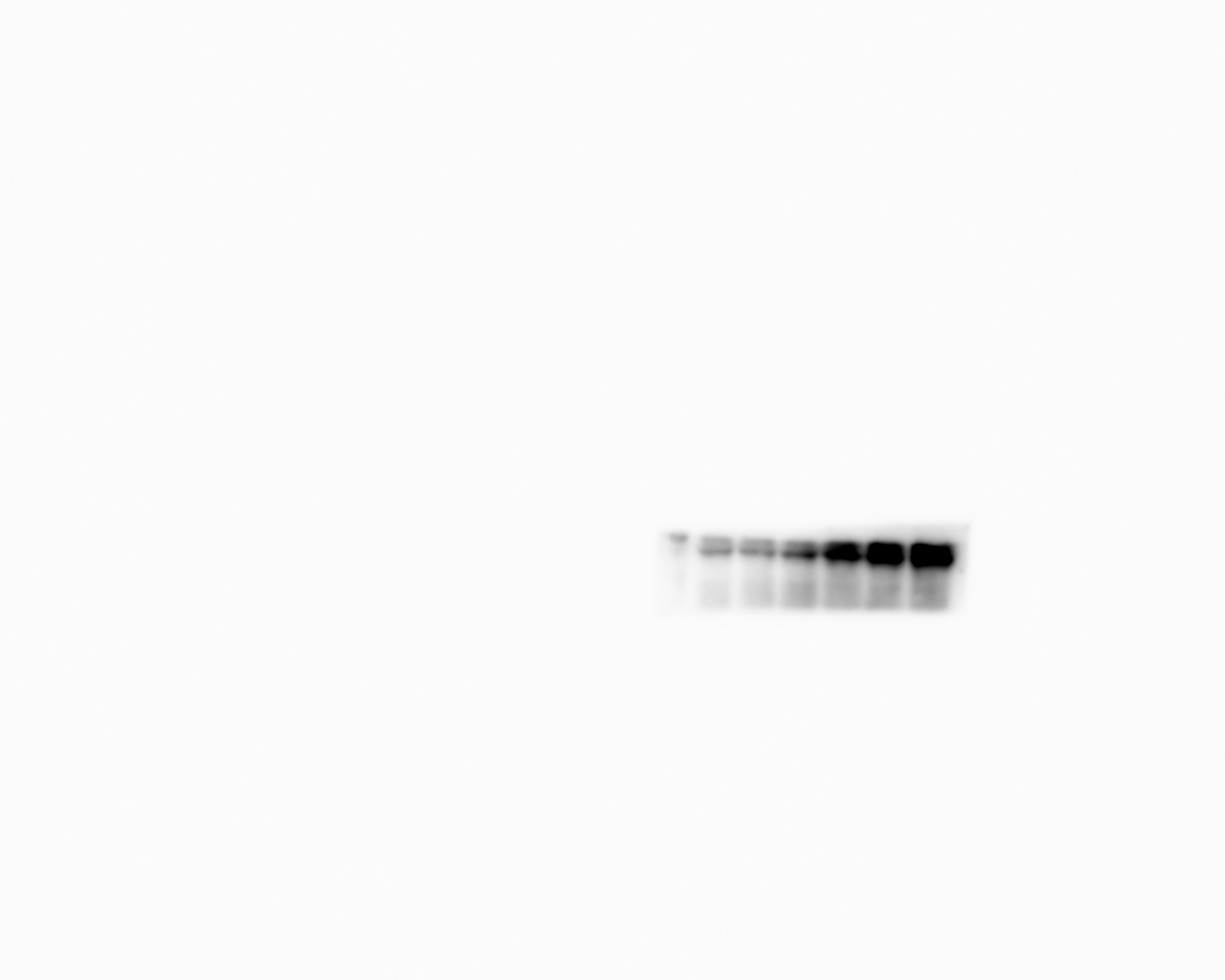

Supplement: Figure 1—figure supplement 1—source data 2. [file elife-89740-fig1-figsupp1-data2.zip › Figure 1-figure supplement 1-data 2/Figure 1-figure supplement 1—data 2-(C).jpg]

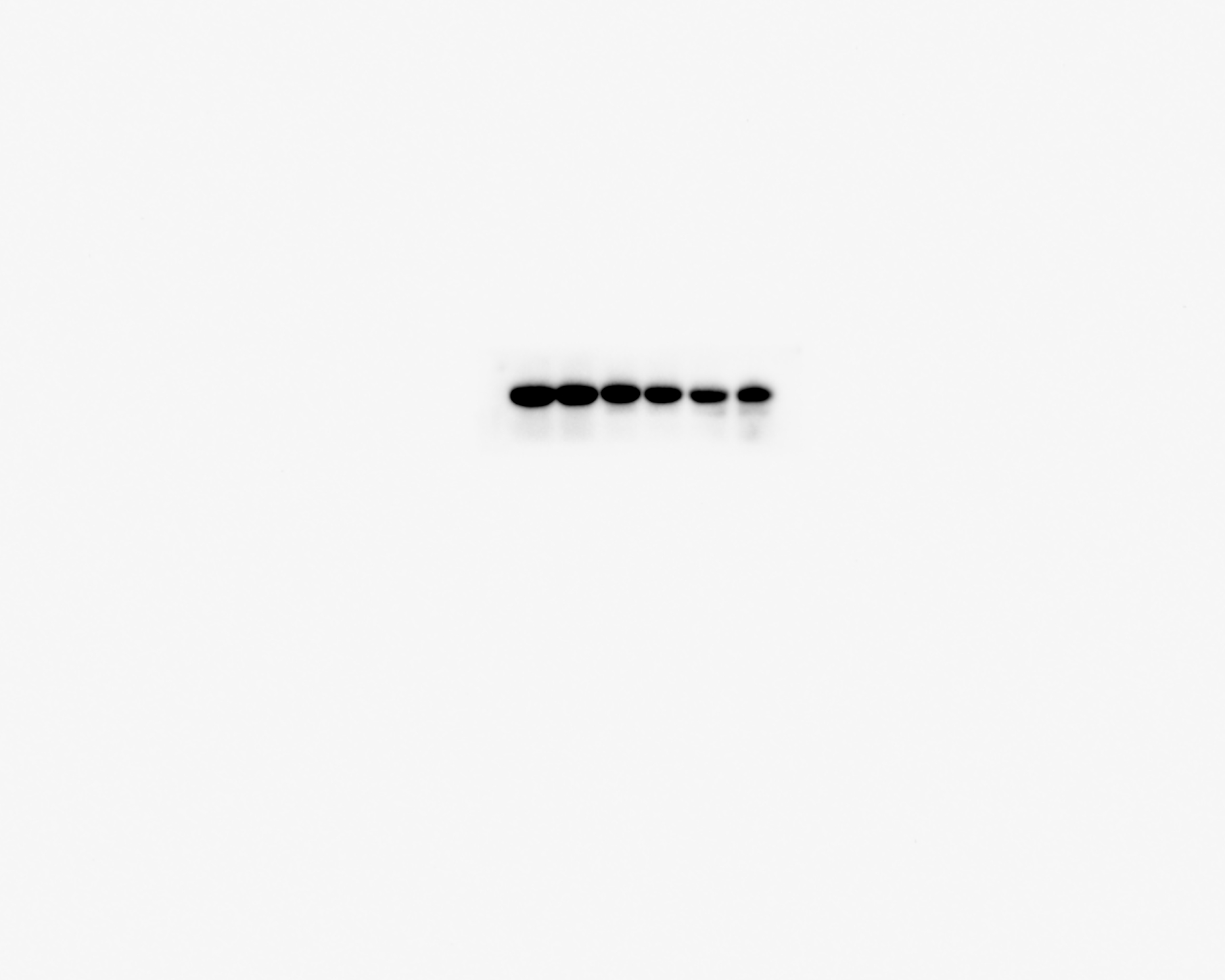

Supplement: Figure 1—figure supplement 1—source data 2. [file elife-89740-fig1-figsupp1-data2.zip › Figure 1-figure supplement 1-data 2/Figure 1-figure supplement 1—data 2-(D).jpg]

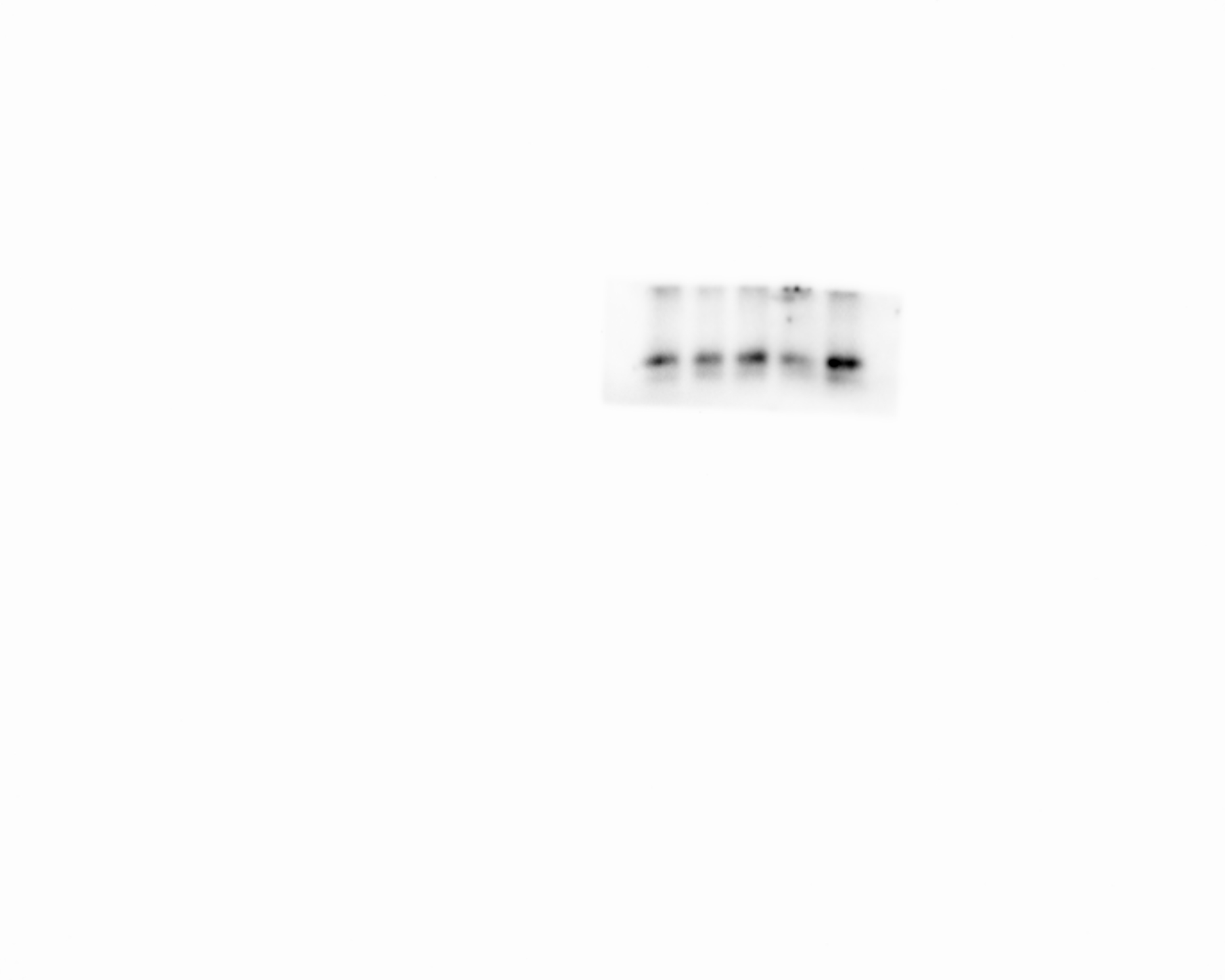

Supplement: Figure 1—figure supplement 1—source data 2. [file elife-89740-fig1-figsupp1-data2.zip › Figure 1-figure supplement 1-data 2/Figure 1-figure supplement 1—data 2-(E).jpg]

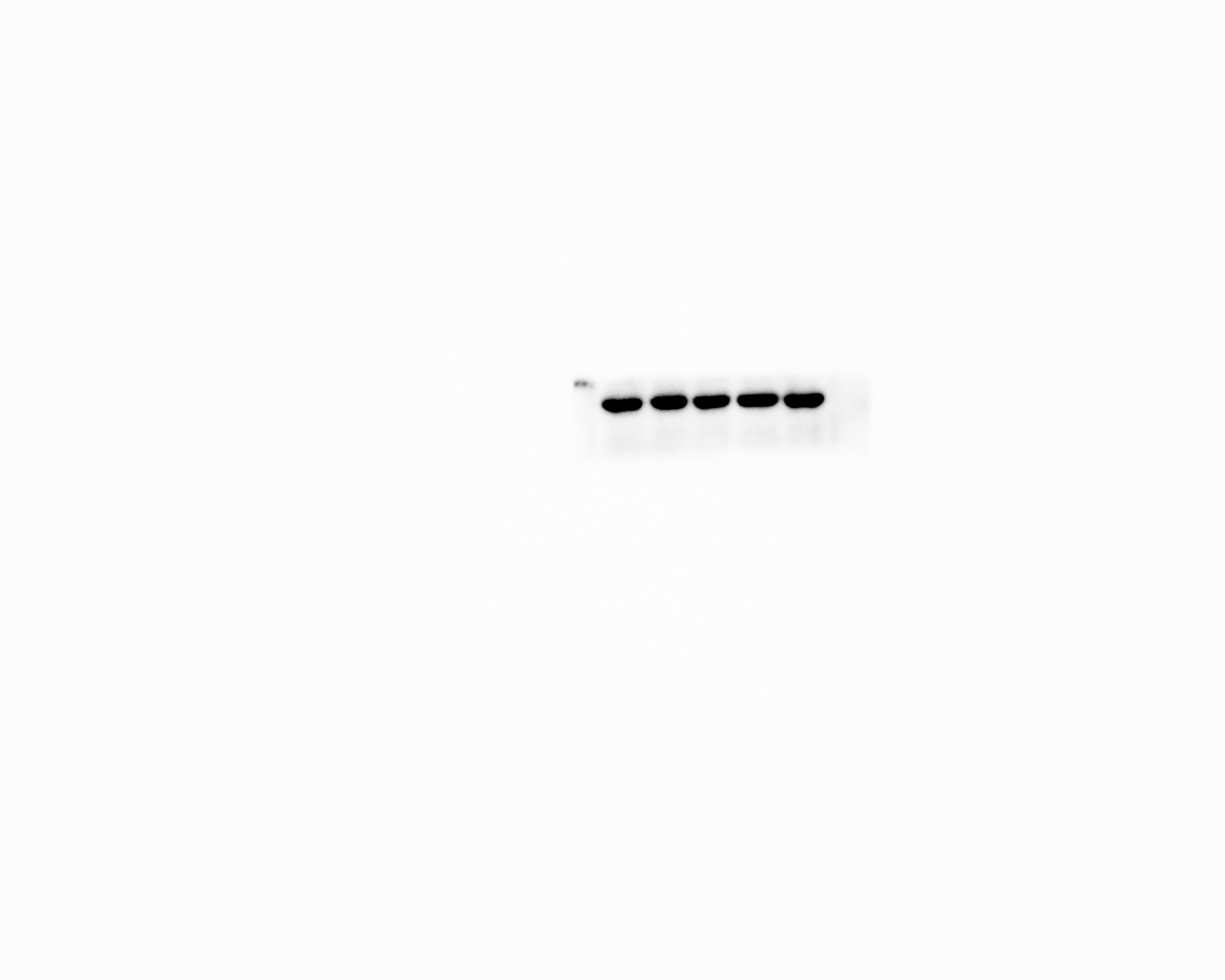

Supplement: Figure 1—figure supplement 1—source data 2. [file elife-89740-fig1-figsupp1-data2.zip › Figure 1-figure supplement 1-data 2/Figure 1-figure supplement 1—data 2-(F).jpg]

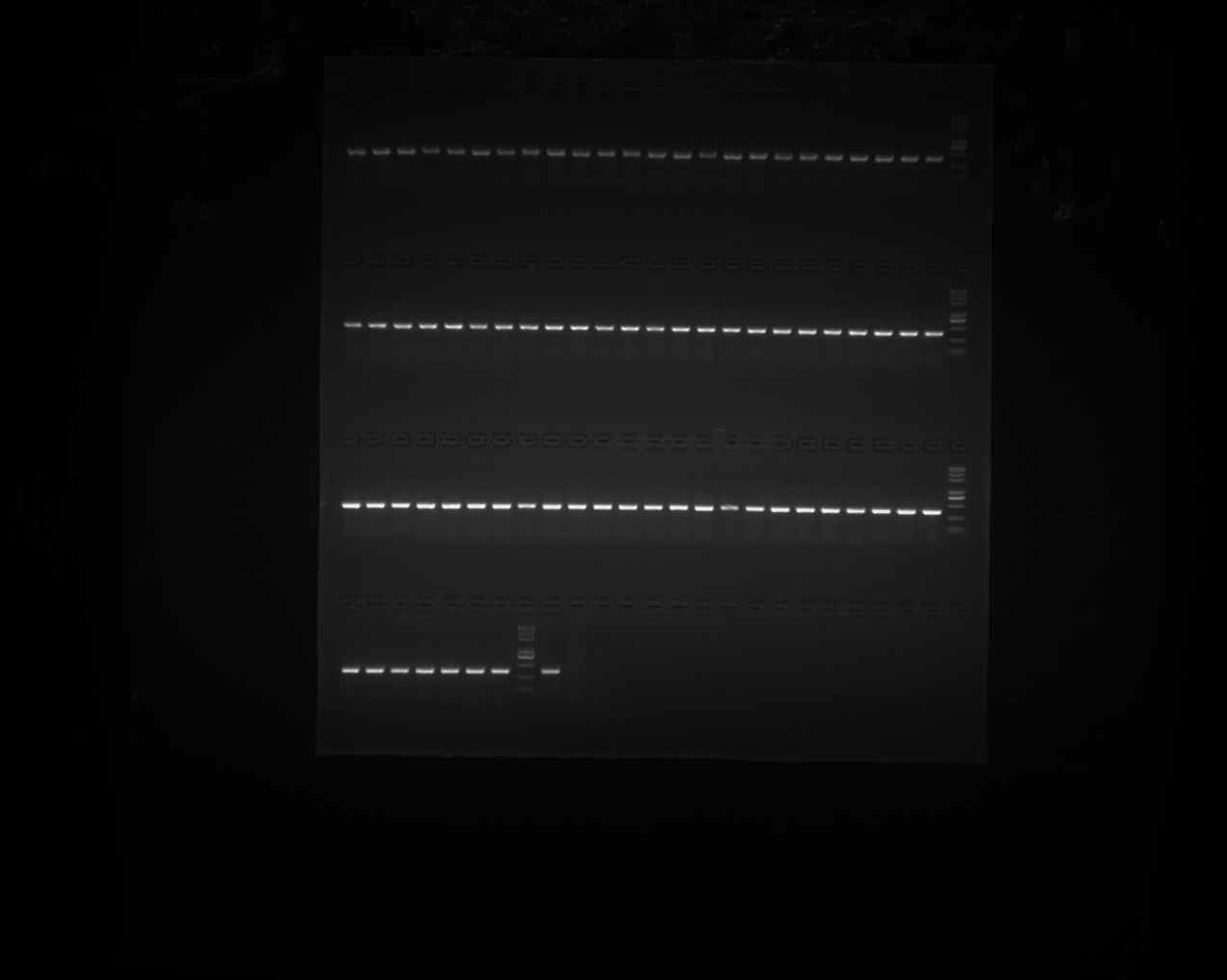

Supplement: Figure 2—source data 2. [file elife-89740-fig2-data2.zip › Figure 2-data 2/Figure 2—data 2-(A).jpg]

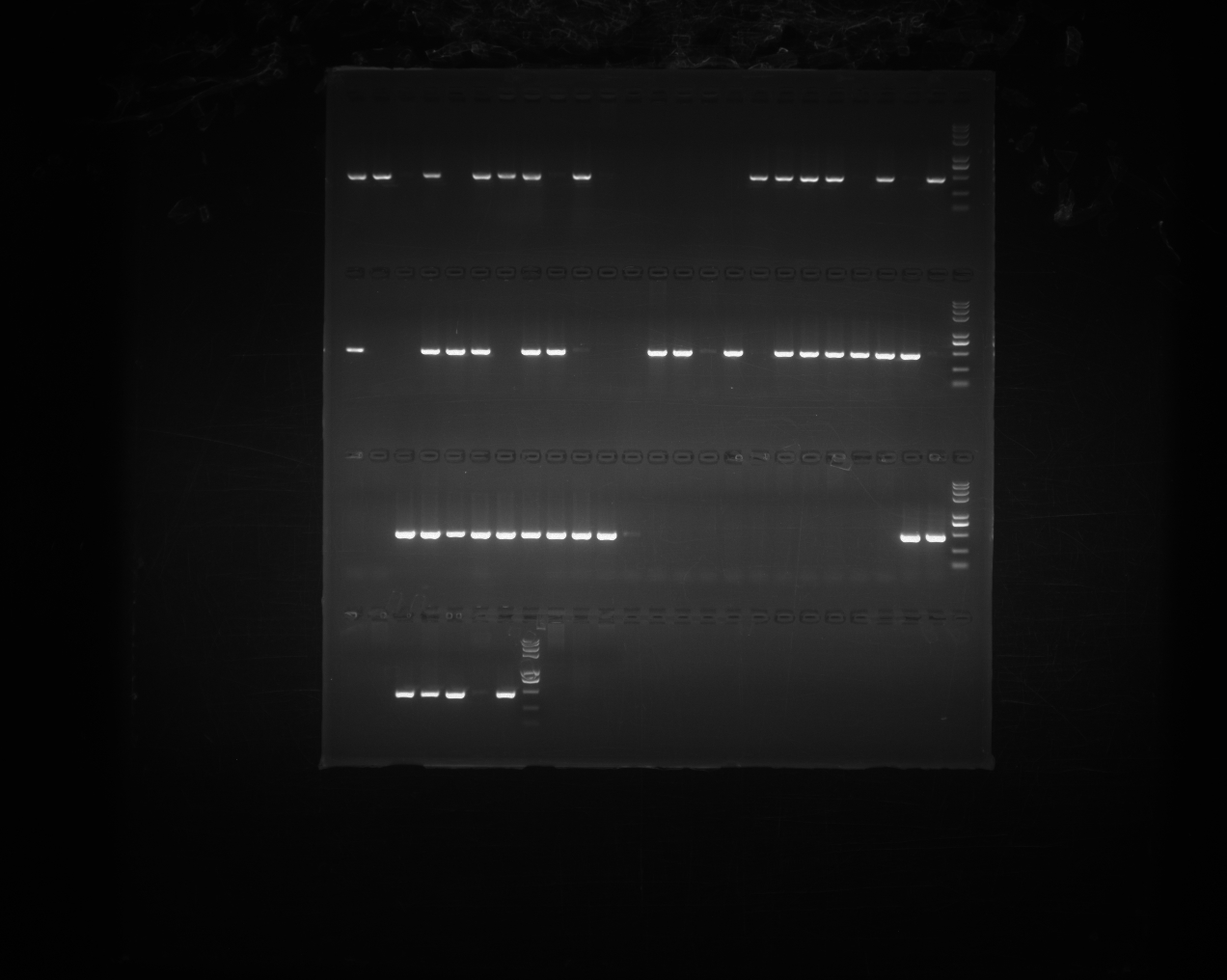

Supplement: Figure 2—source data 2. [file elife-89740-fig2-data2.zip › Figure 2-data 2/Figure 2—data 2-(B).jpg]

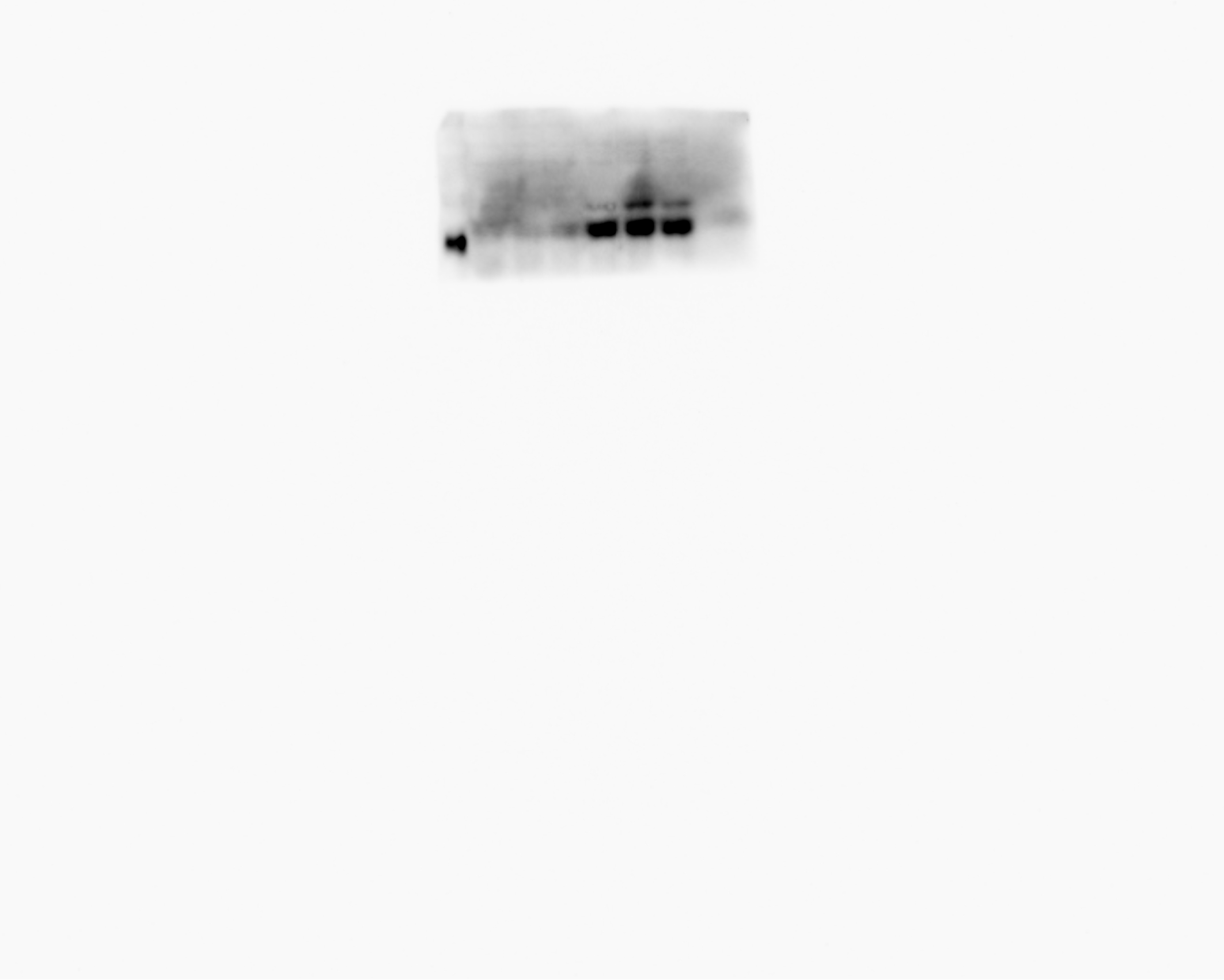

Supplement: Figure 2—source data 2. [file elife-89740-fig2-data2.zip › Figure 2-data 2/Figure 2—data 2-(C).jpg]

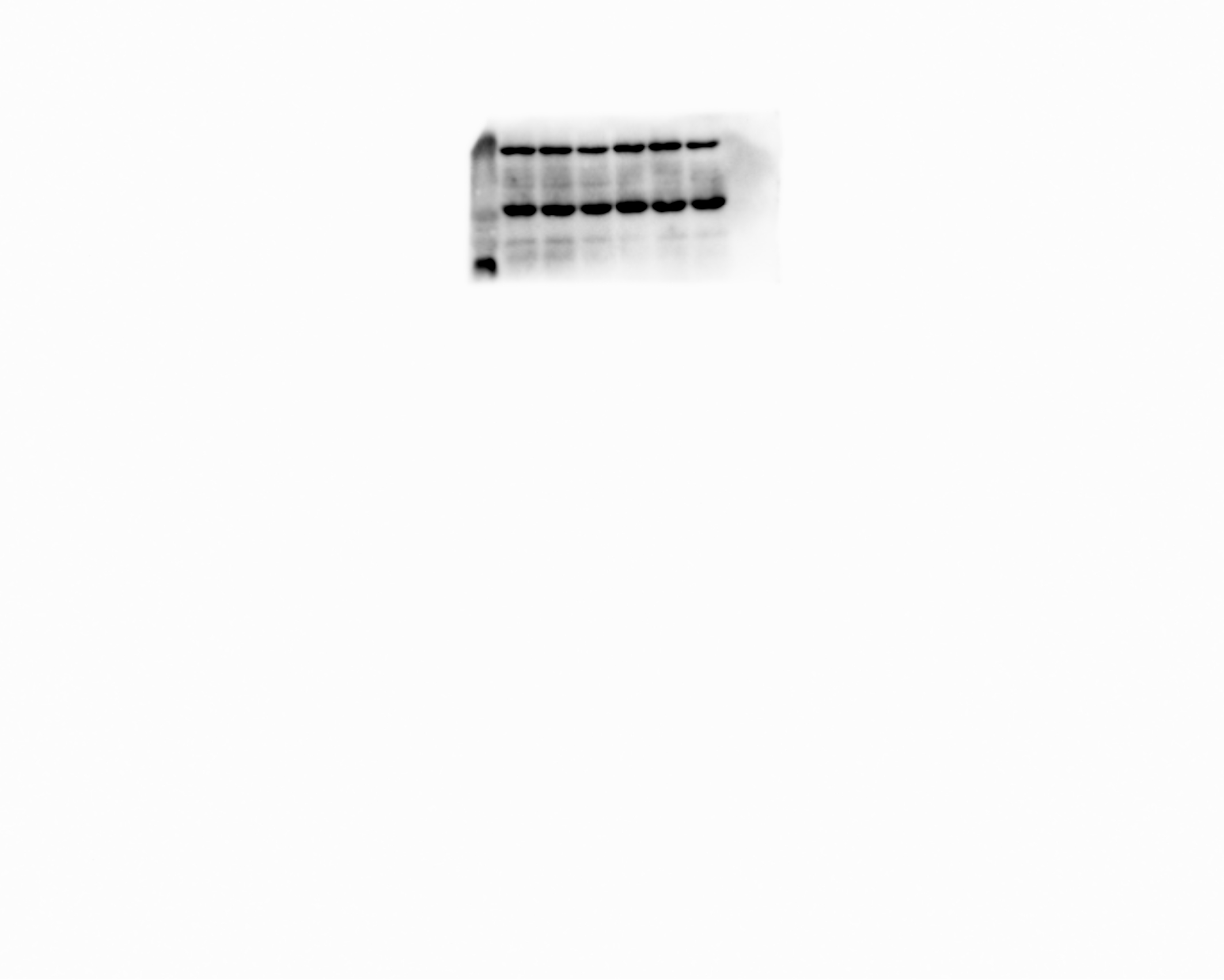

Supplement: Figure 2—source data 2. [file elife-89740-fig2-data2.zip › Figure 2-data 2/Figure 2—data 2-(D).jpg]

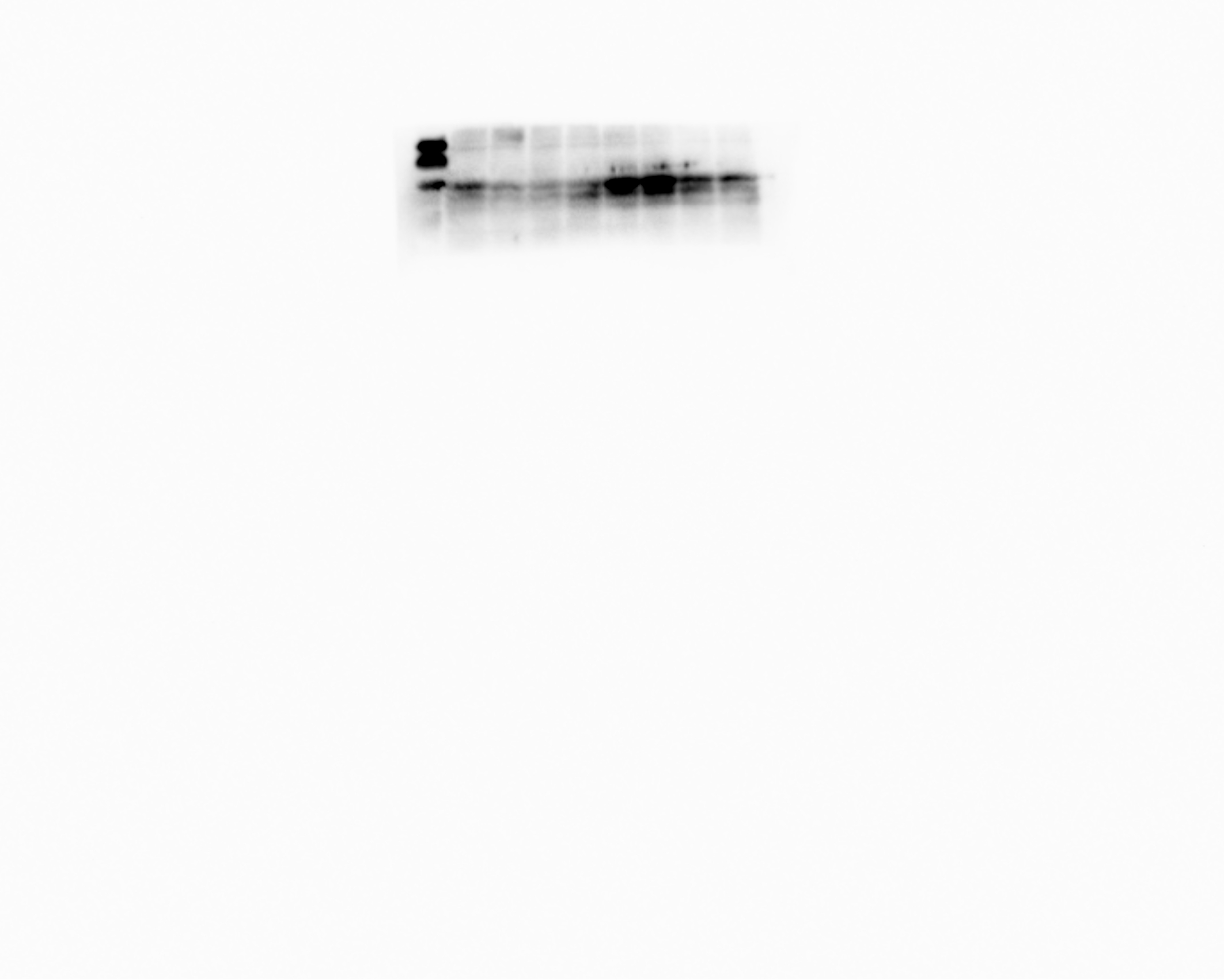

Supplement: Figure 2—source data 2. [file elife-89740-fig2-data2.zip › Figure 2-data 2/Figure 2—data 2-(E).jpg]

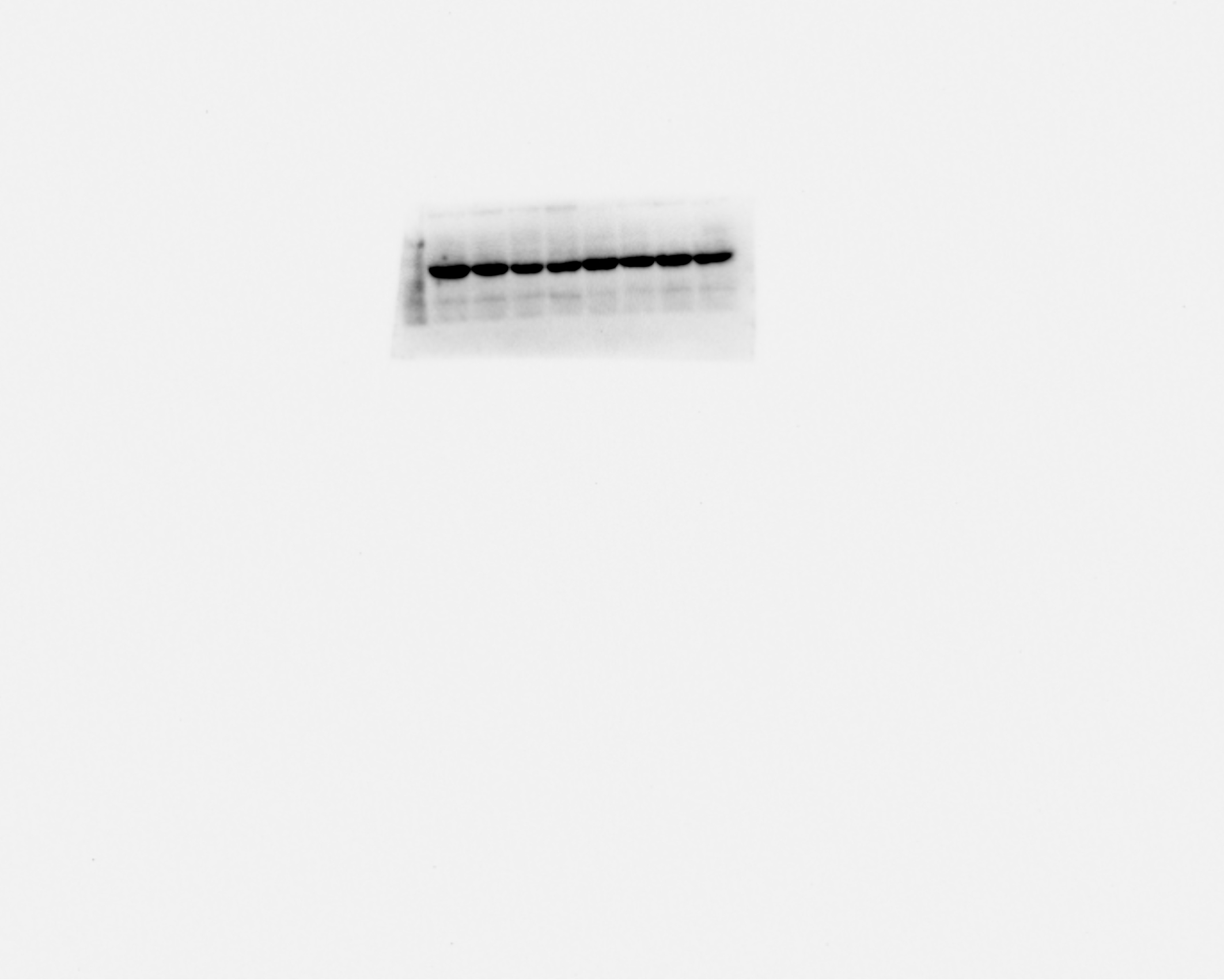

Supplement: Figure 2—source data 2. [file elife-89740-fig2-data2.zip › Figure 2-data 2/Figure 2—data 2-(F).jpg]

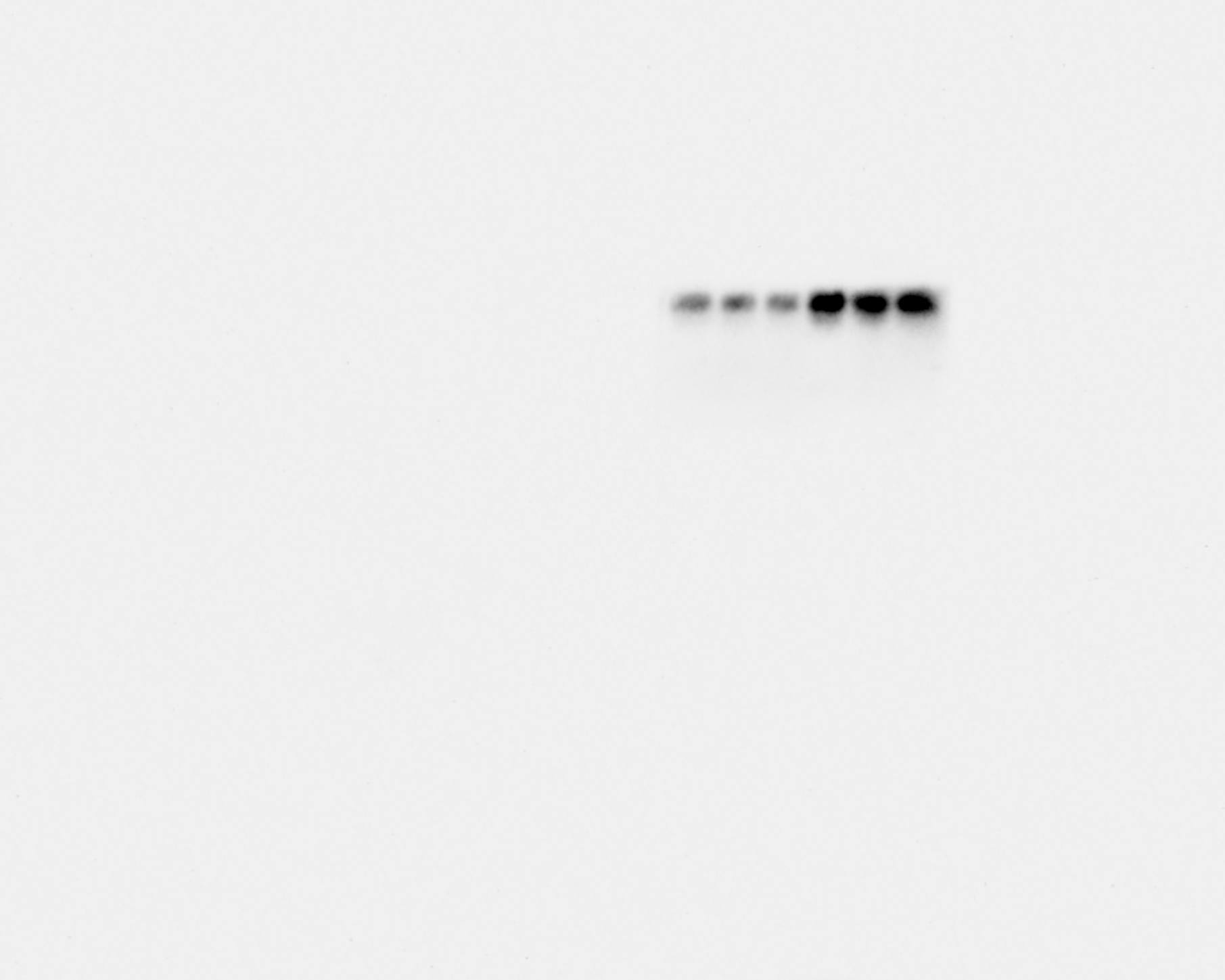

Supplement: Figure 2—figure supplement 1—source data 2. [file elife-89740-fig2-figsupp1-data2.zip › Figure 2-figure supplement 1-data 2/Figure 2-figure supplement 1—data 2-(A).jpg]

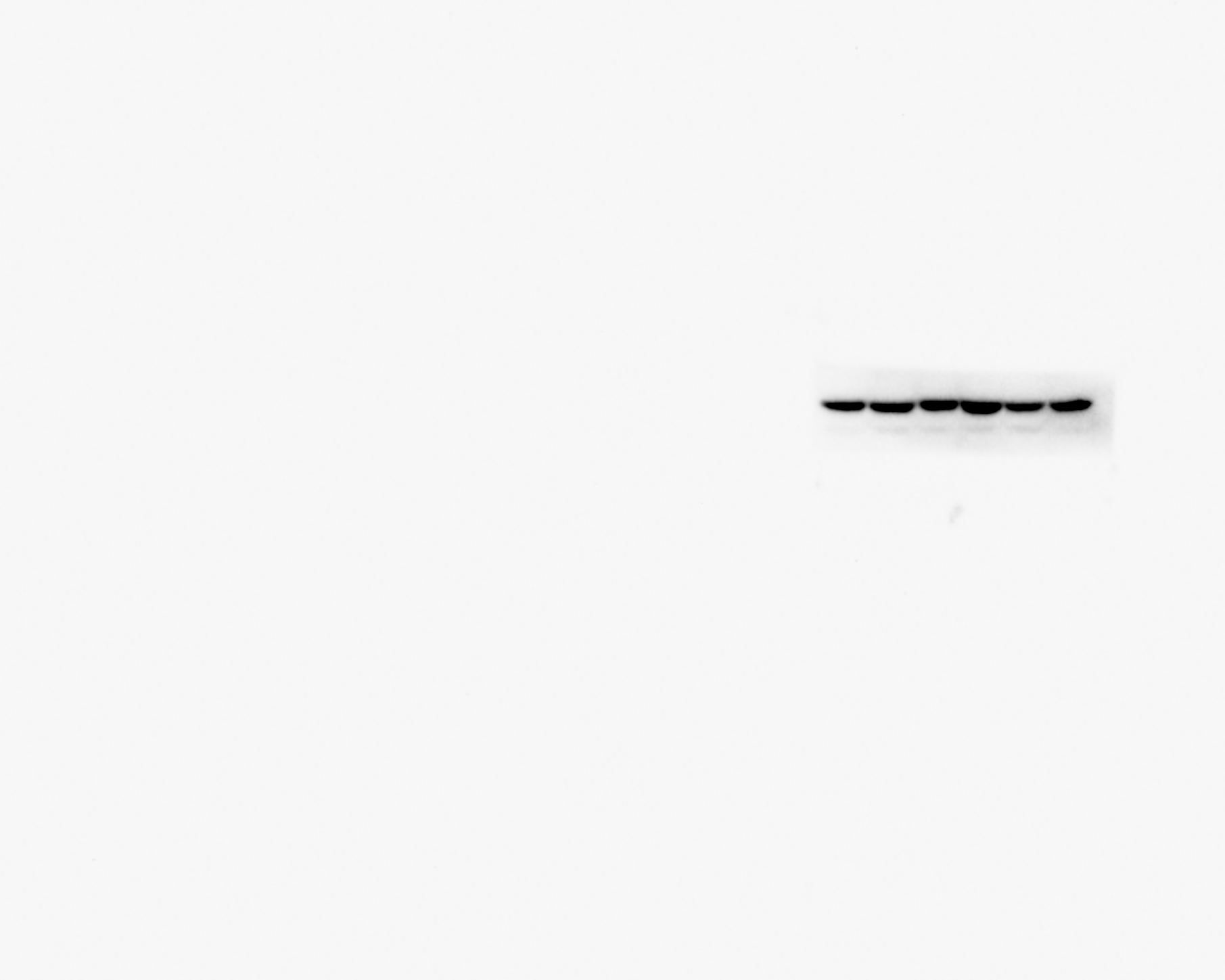

Supplement: Figure 2—figure supplement 1—source data 2. [file elife-89740-fig2-figsupp1-data2.zip › Figure 2-figure supplement 1-data 2/Figure 2-figure supplement 1—data 2-(B).jpg]

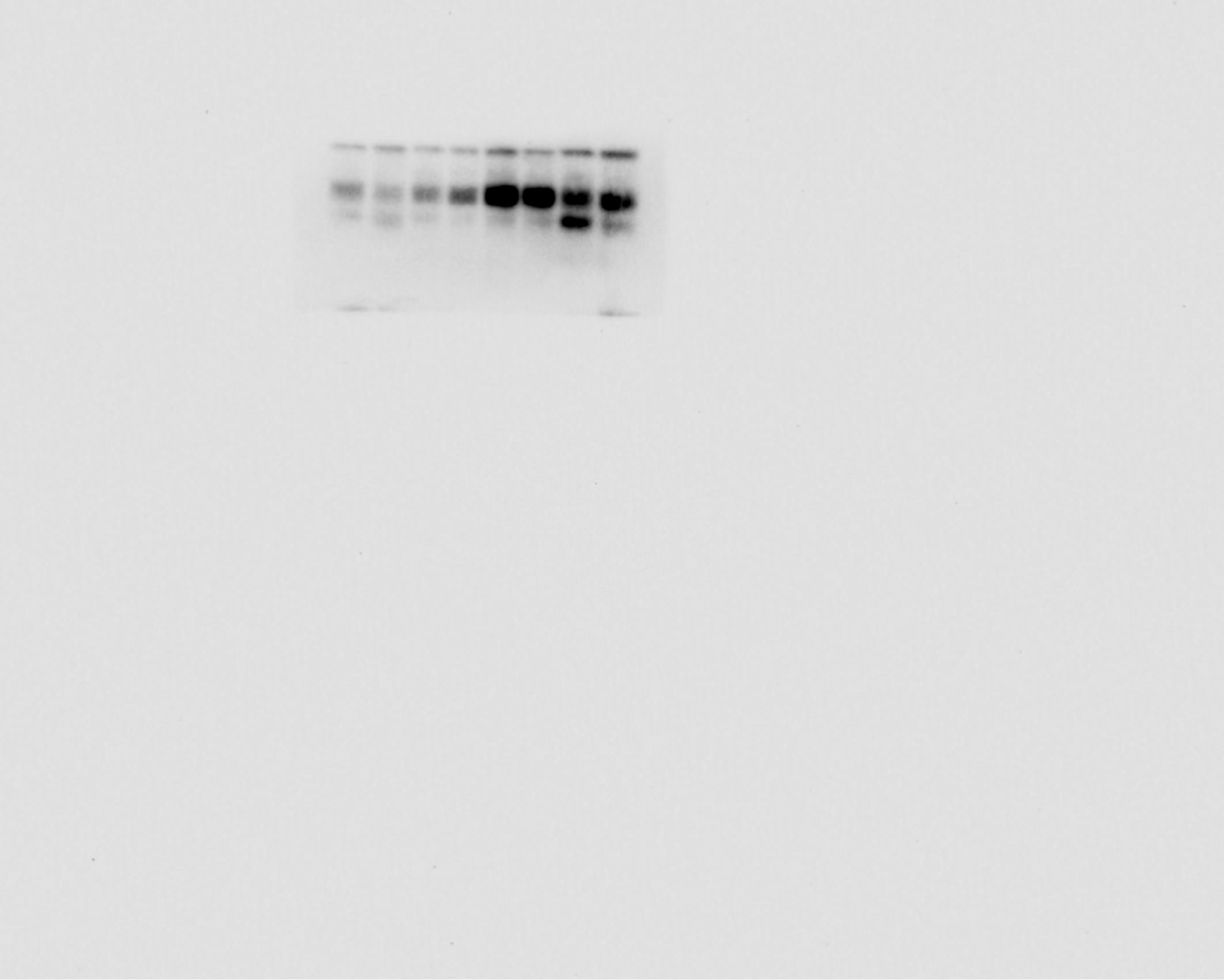

Supplement: Figure 2—figure supplement 1—source data 2. [file elife-89740-fig2-figsupp1-data2.zip › Figure 2-figure supplement 1-data 2/Figure 2-figure supplement 1—data 2-(C).jpg]

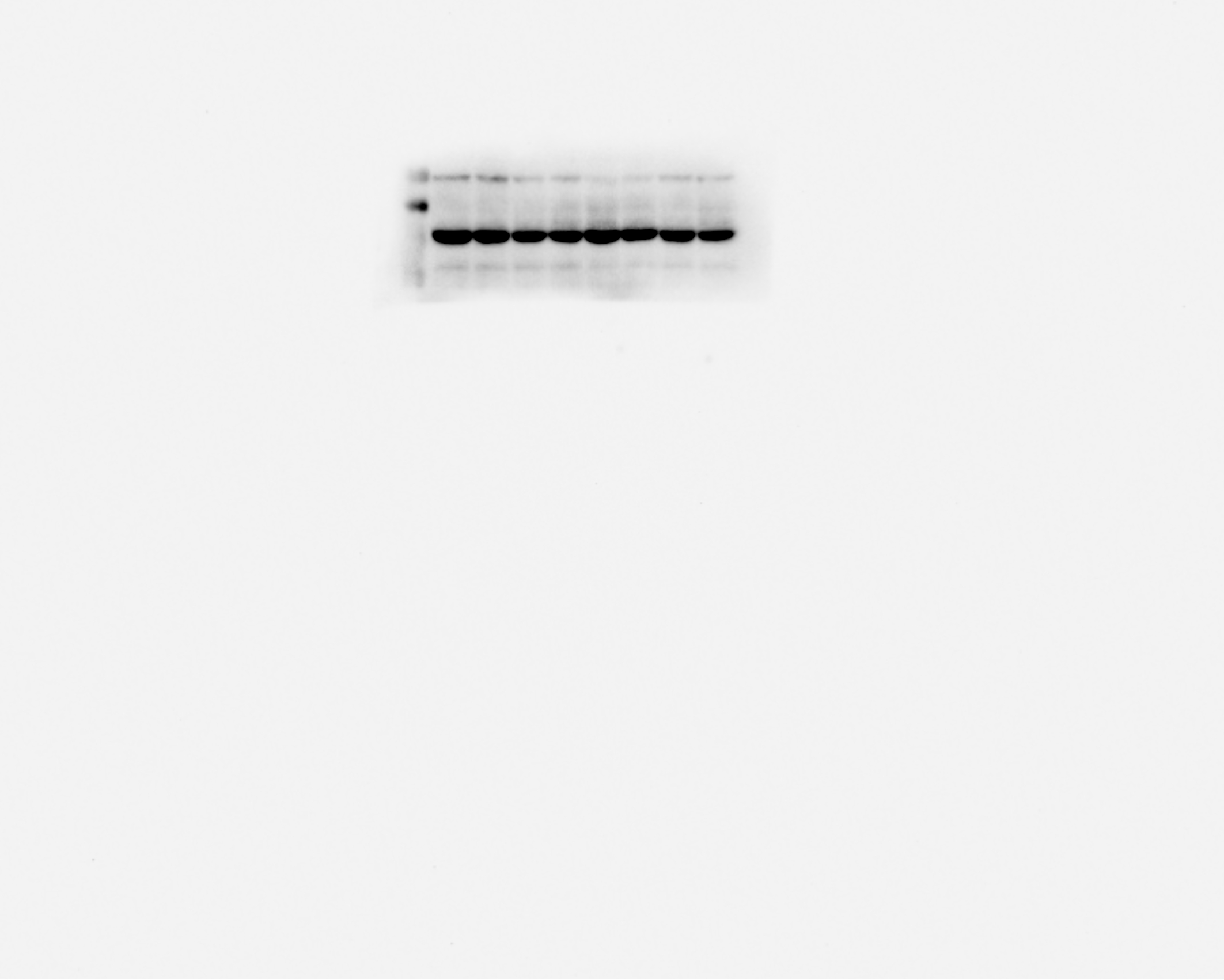

Supplement: Figure 2—figure supplement 1—source data 2. [file elife-89740-fig2-figsupp1-data2.zip › Figure 2-figure supplement 1-data 2/Figure 2-figure supplement 1—data 2-(D).jpg]

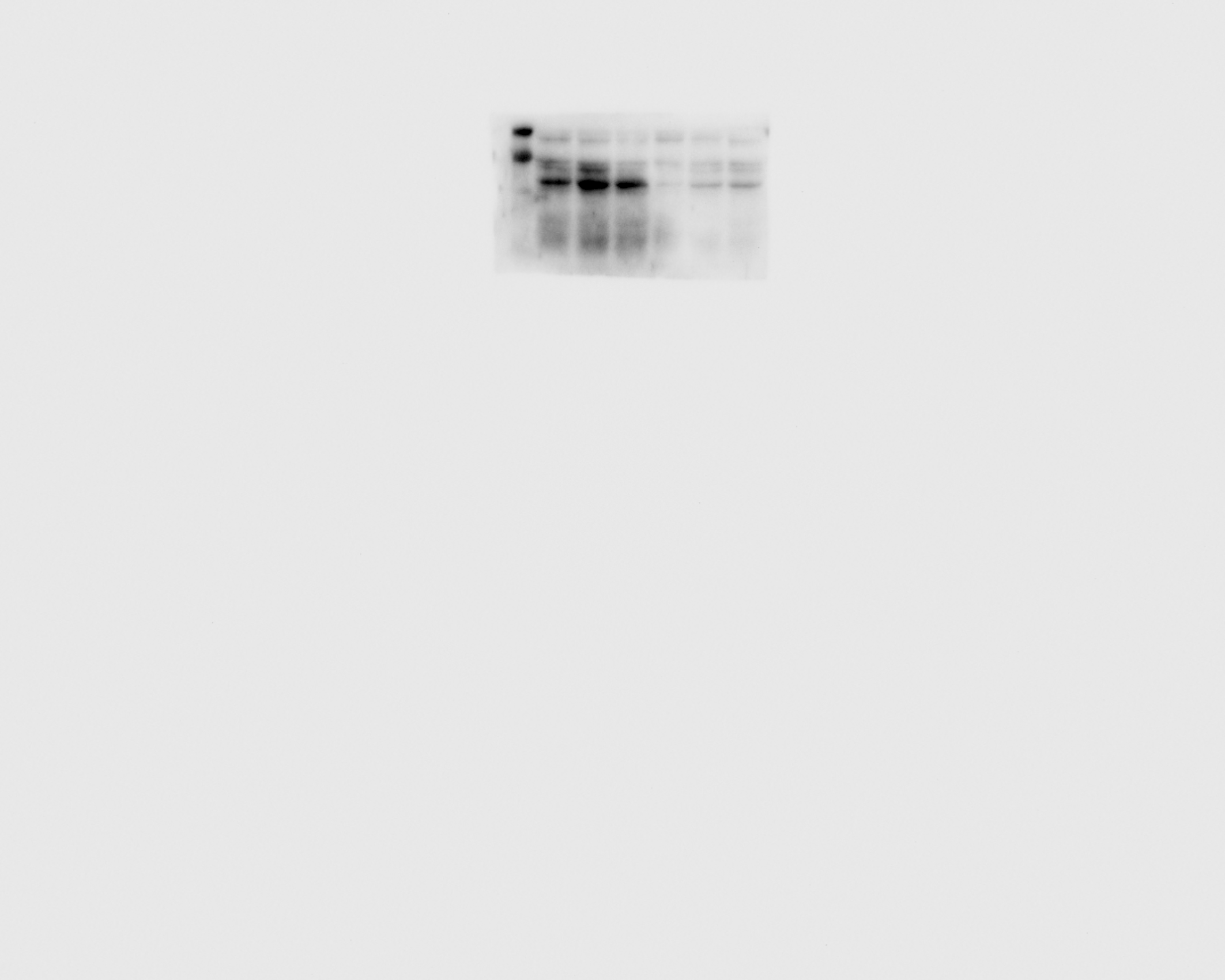

Supplement: Figure 3—source data 2. [file elife-89740-fig3-data2.zip › Figure 3-data 2/Figure 3—data 2-(A).tif]

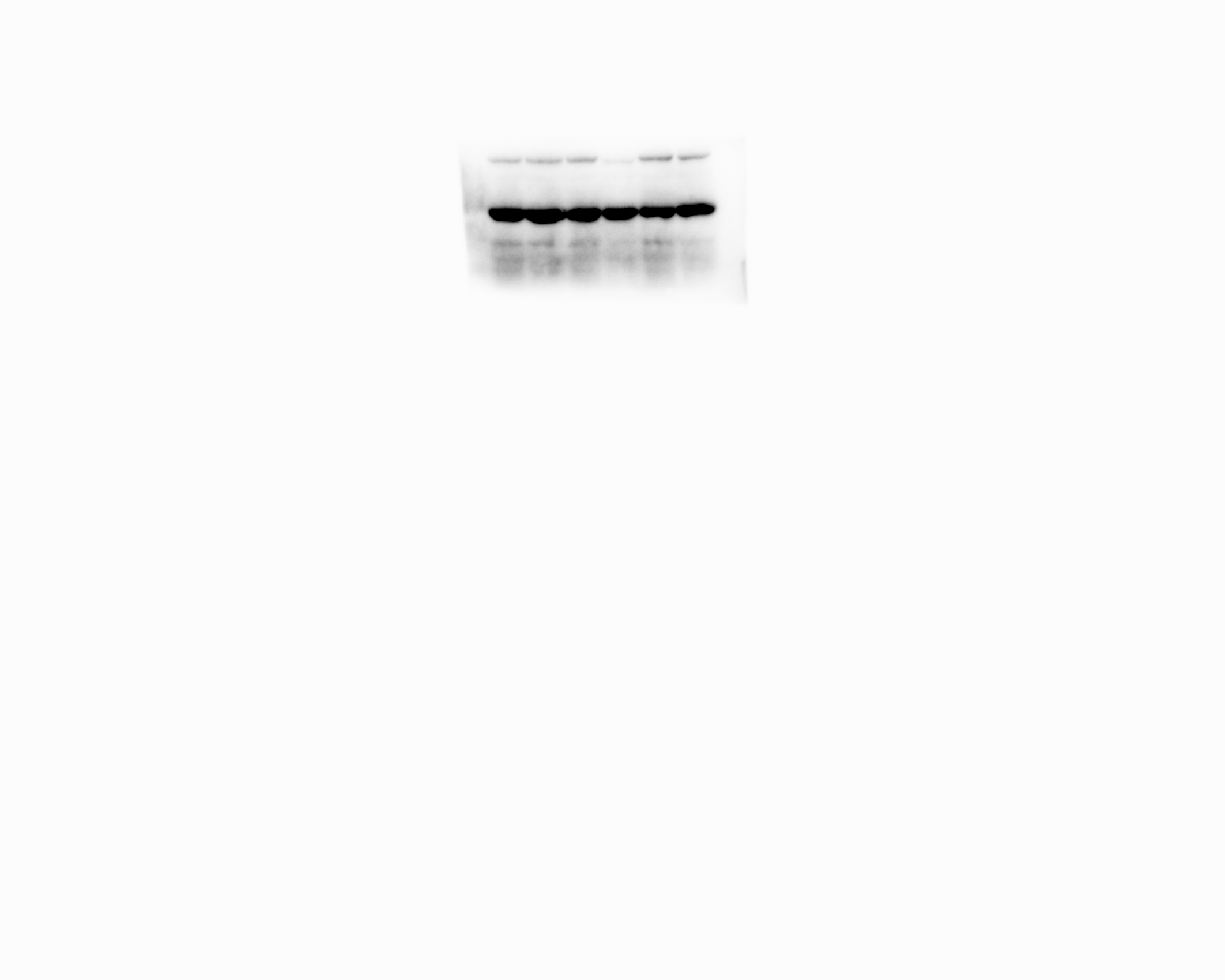

Supplement: Figure 3—source data 2. [file elife-89740-fig3-data2.zip › Figure 3-data 2/Figure 3—data 2-(B).tif]

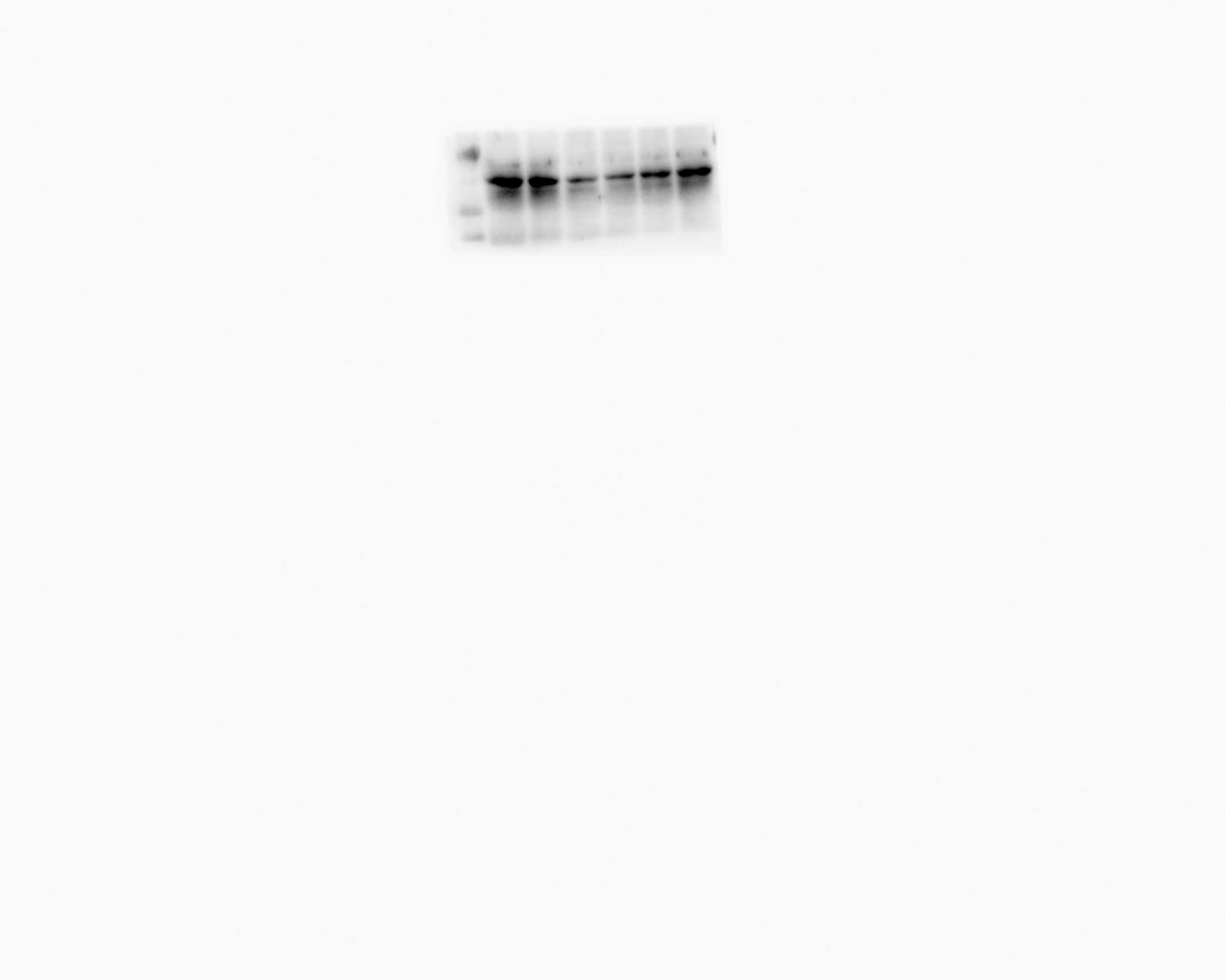

Supplement: Figure 3—source data 2. [file elife-89740-fig3-data2.zip › Figure 3-data 2/Figure 3—data 2-(C).tif]

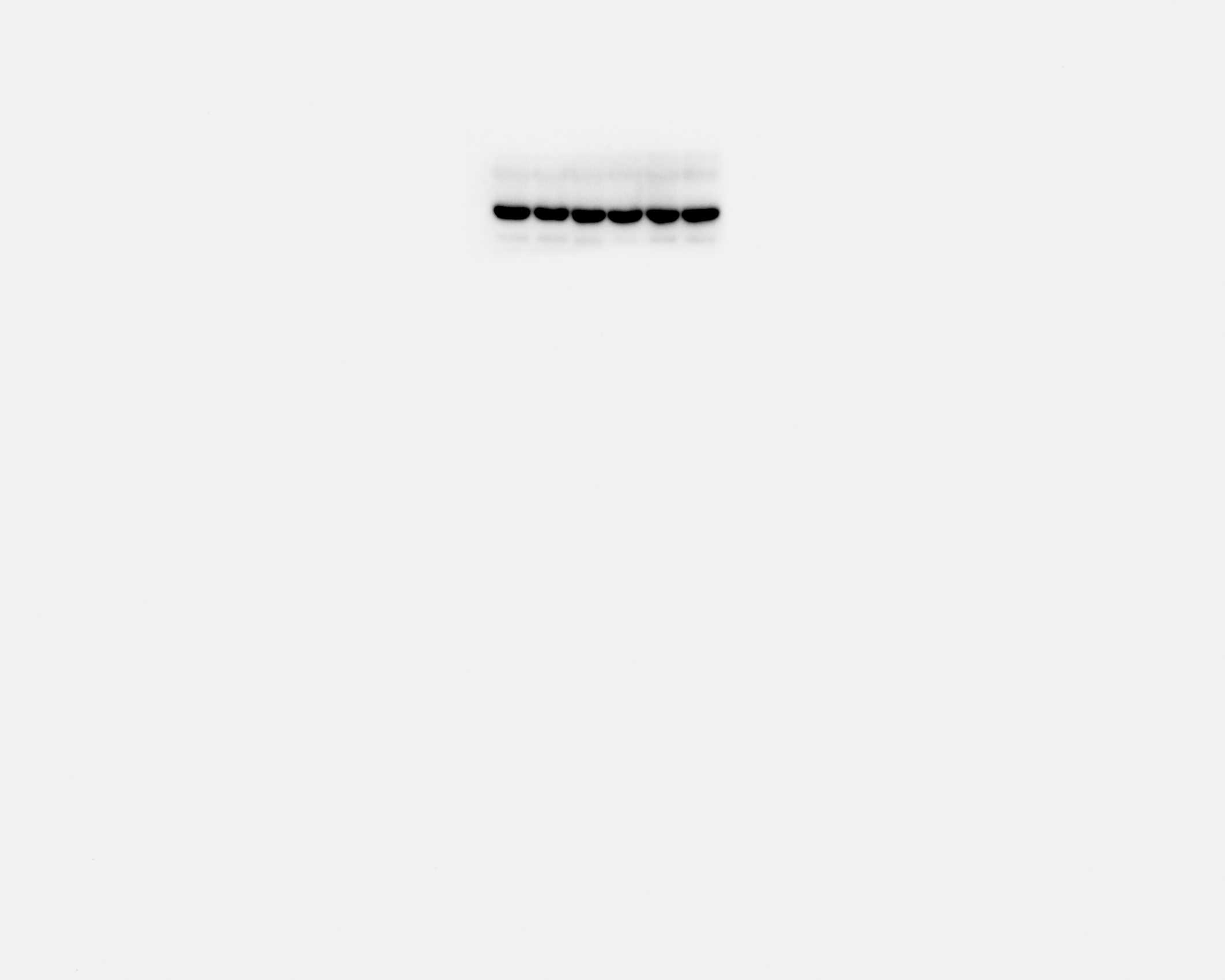

Supplement: Figure 3—source data 2. [file elife-89740-fig3-data2.zip › Figure 3-data 2/Figure 3—data 2-(D).tif]

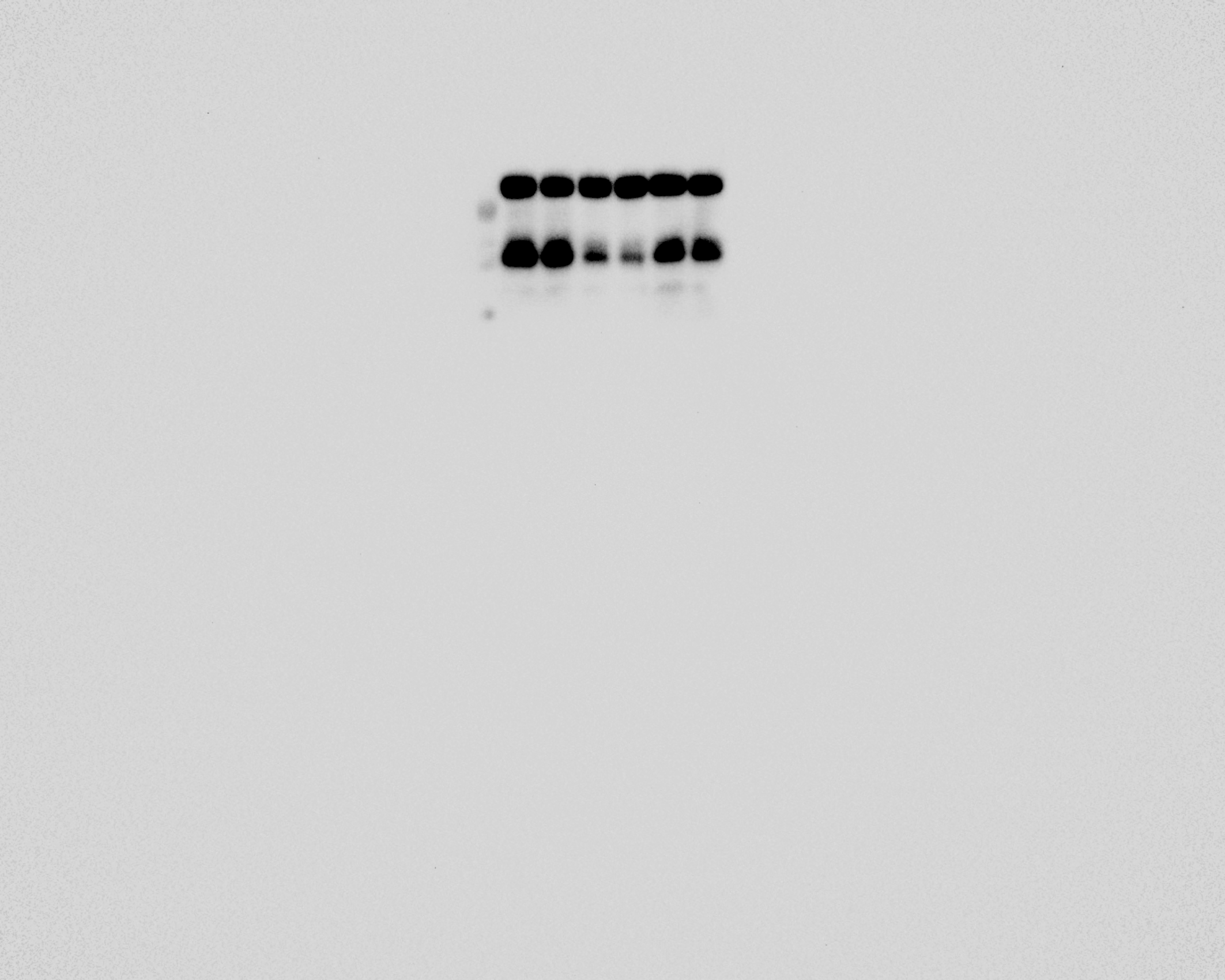

Supplement: Figure 3—source data 2. [file elife-89740-fig3-data2.zip › Figure 3-data 2/Figure 3—data 2-(E).tif]

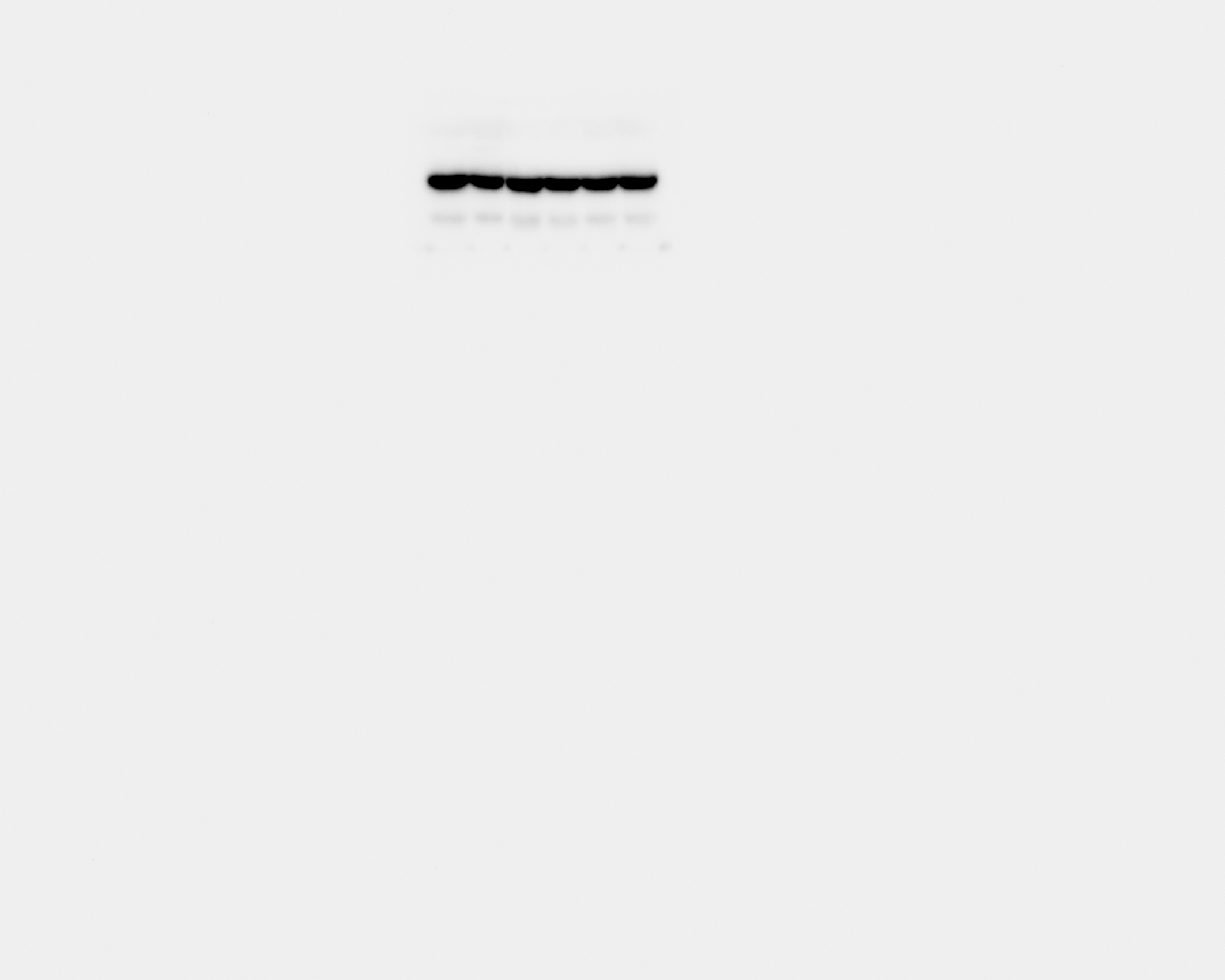

Supplement: Figure 3—source data 2. [file elife-89740-fig3-data2.zip › Figure 3-data 2/Figure 3—data 2-(F).tif]

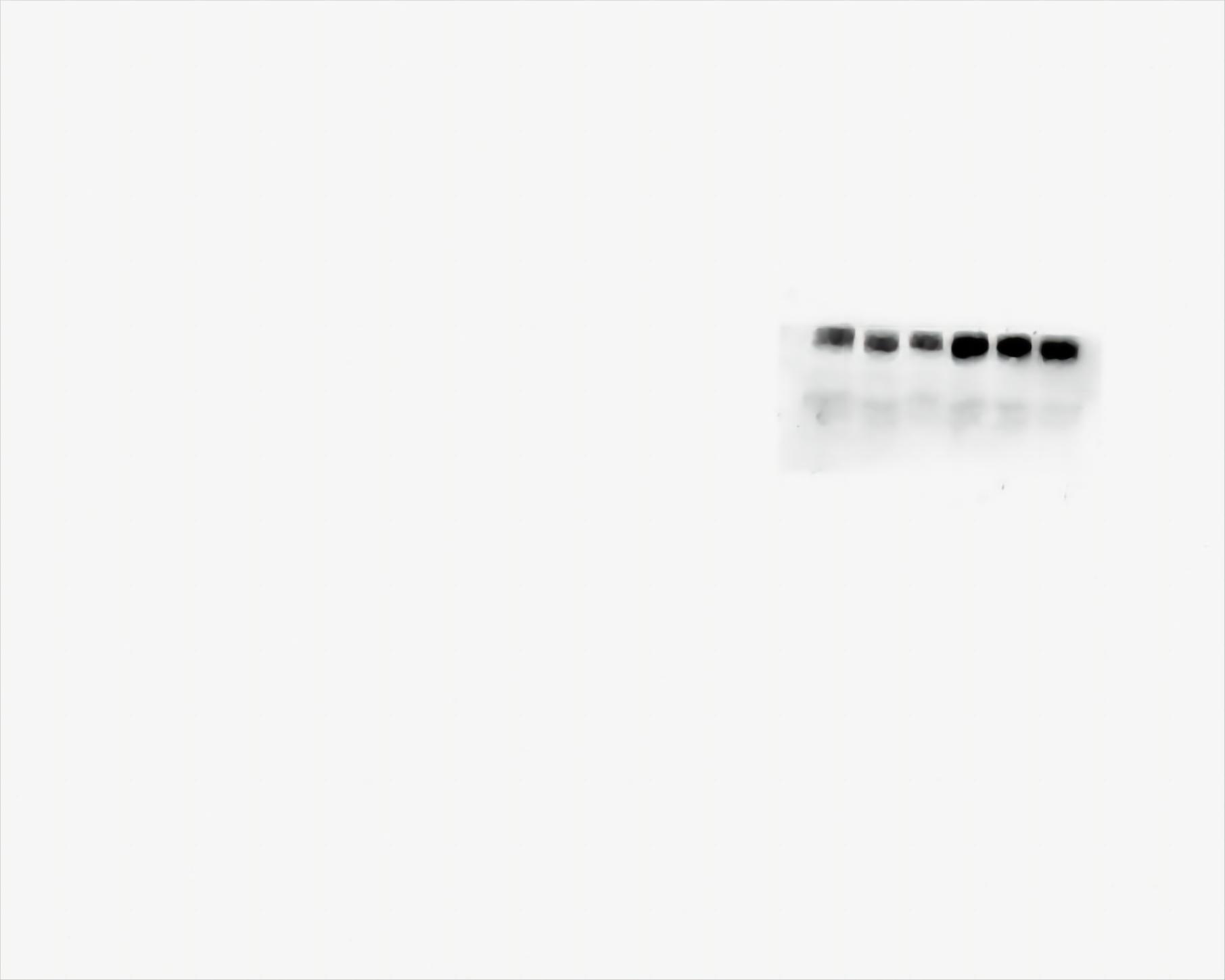

Supplement: Figure 5—source data 2. [file elife-89740-fig5-data2.zip › Figure 5-data 2/Figure 5—data 2-(A).jpg]

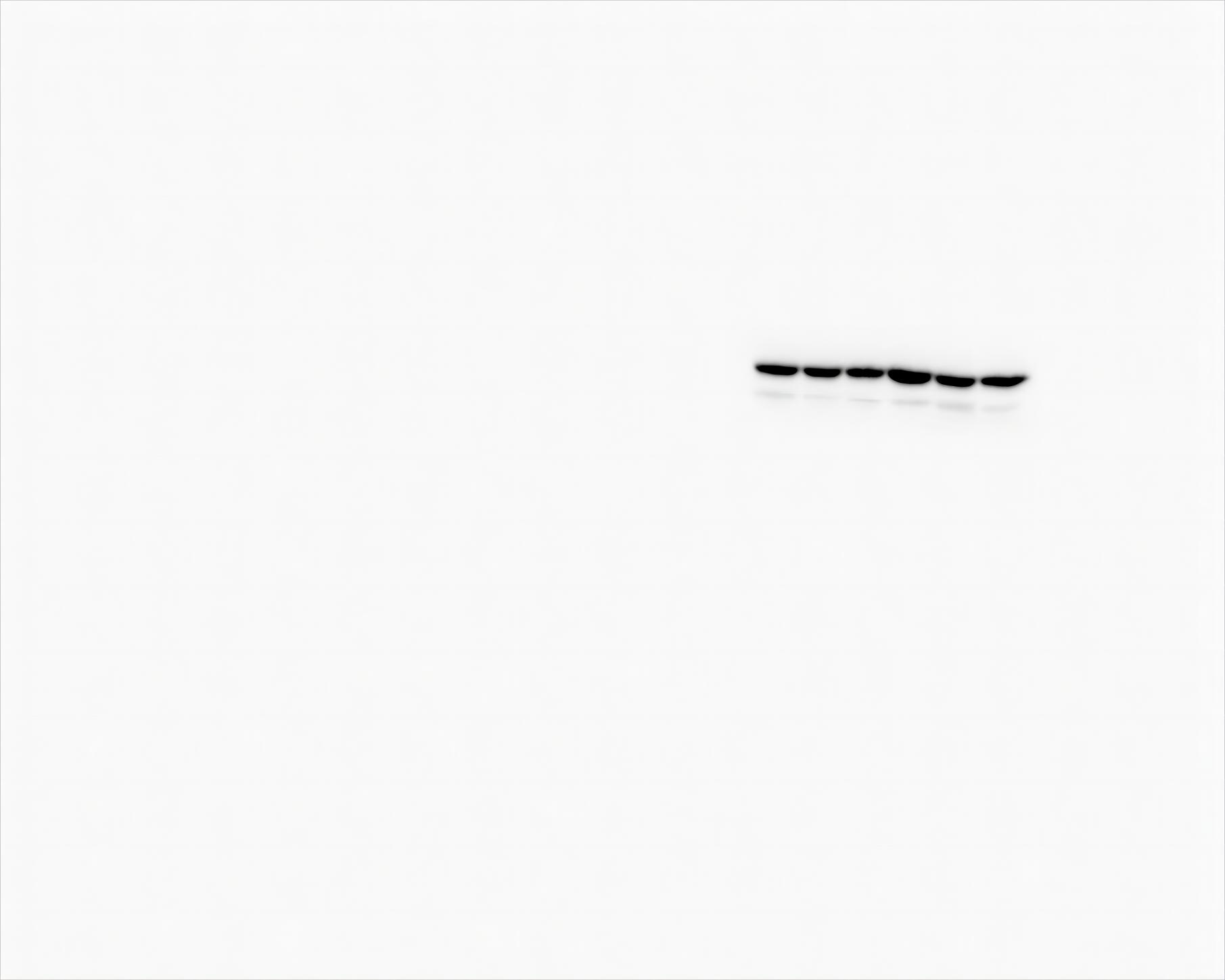

Supplement: Figure 5—source data 2. [file elife-89740-fig5-data2.zip › Figure 5-data 2/Figure 5—data 2-(B).jpg]

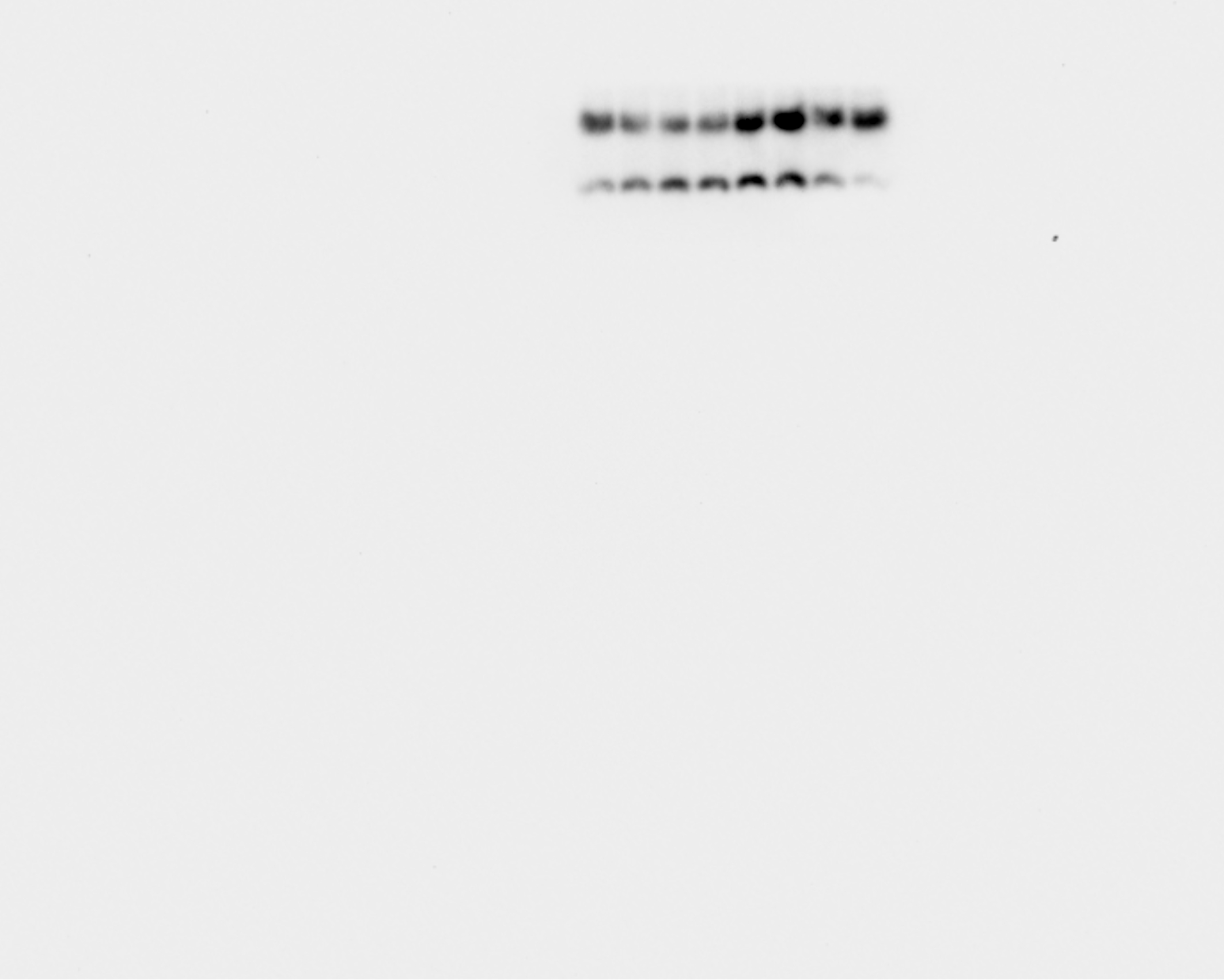

Supplement: Figure 5—source data 2. [file elife-89740-fig5-data2.zip › Figure 5-data 2/Figure 5—data 2-(C).jpg]

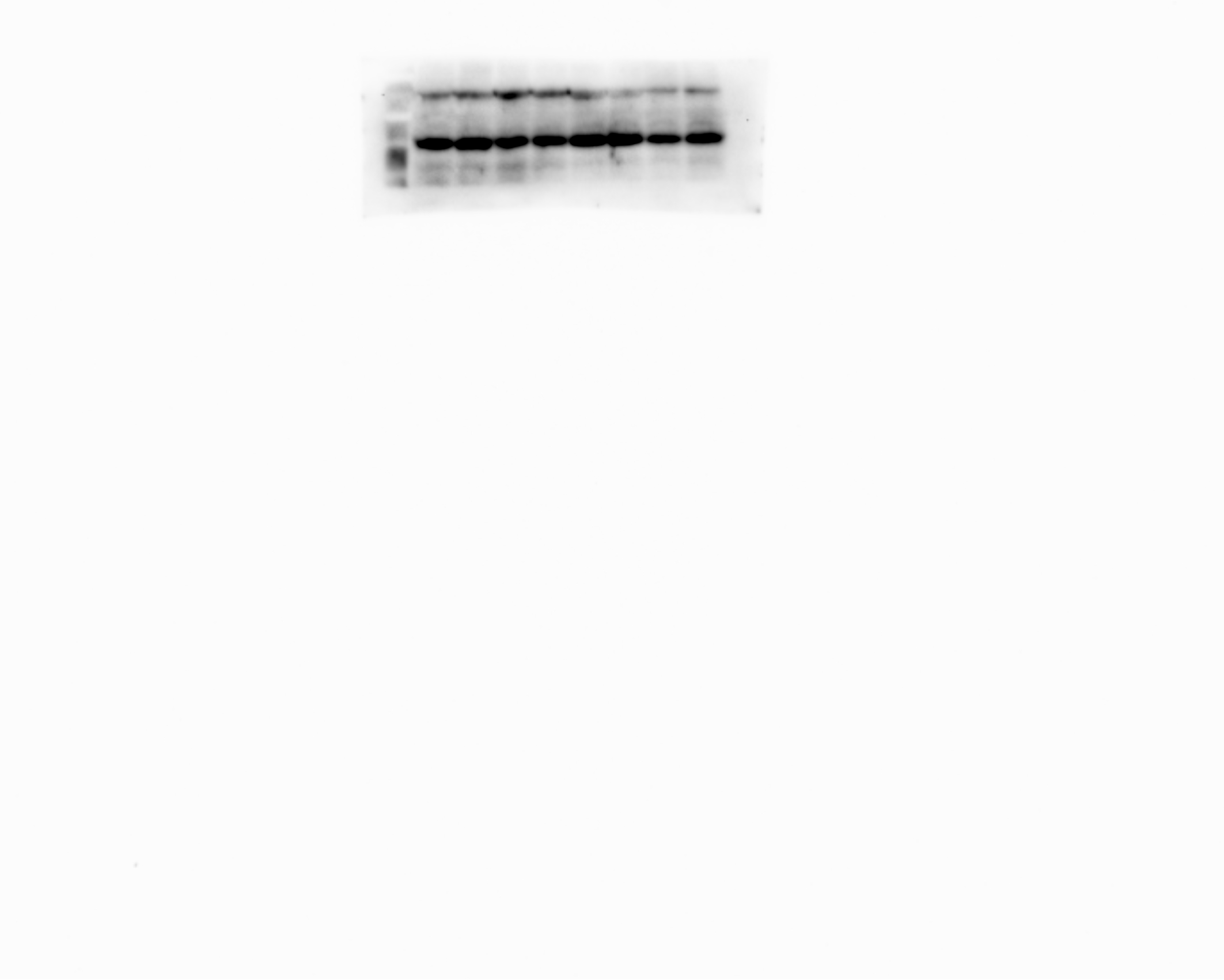

Supplement: Figure 5—source data 2. [file elife-89740-fig5-data2.zip › Figure 5-data 2/Figure 5—data 2-(D).jpg]

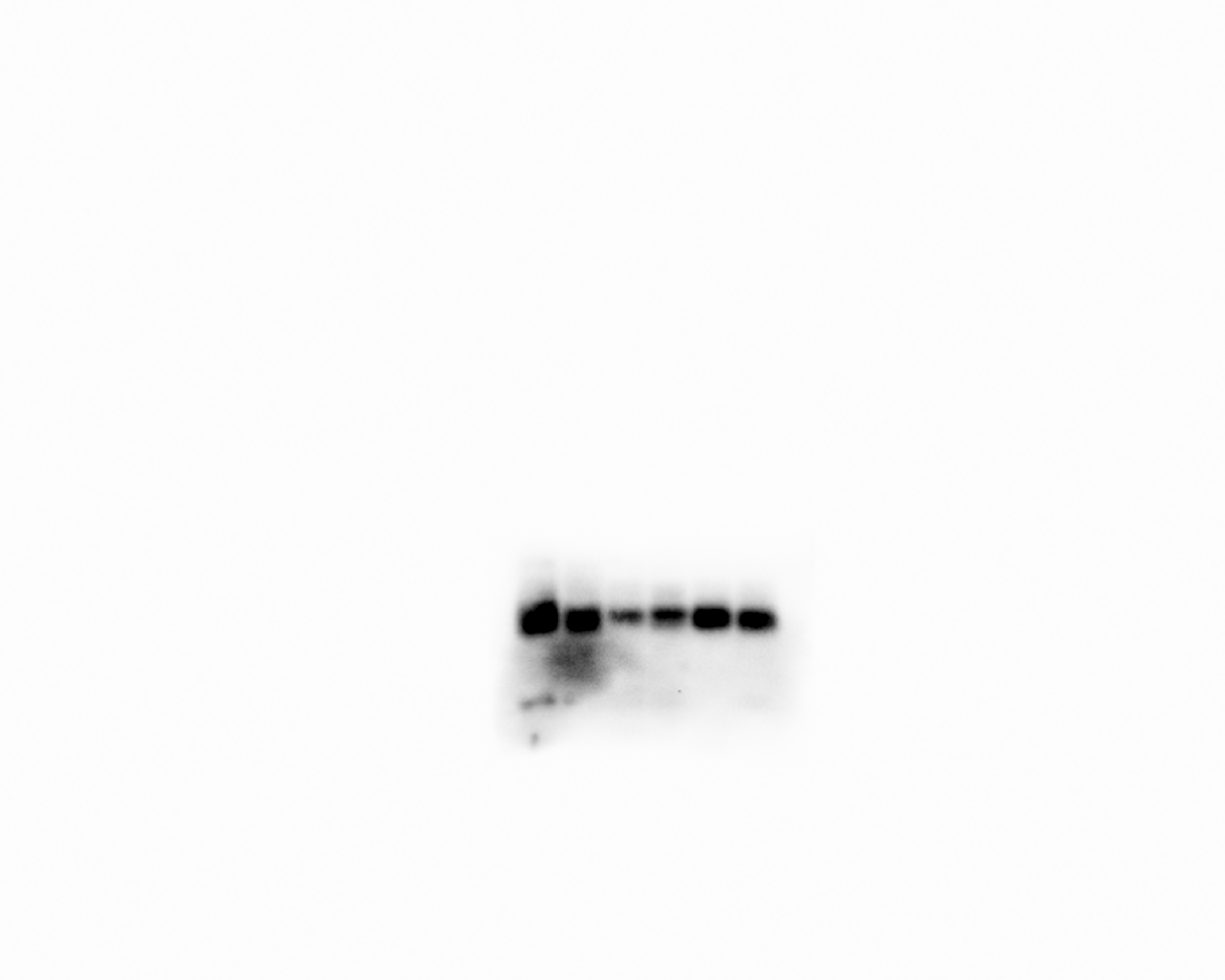

Supplement: Figure 5—figure supplement 1—source data 2. [file elife-89740-fig5-figsupp1-data2.zip › Figure 5-figure supplement 1-data 2/Figure 5-figure supplement 1—data 2-(A).tif]

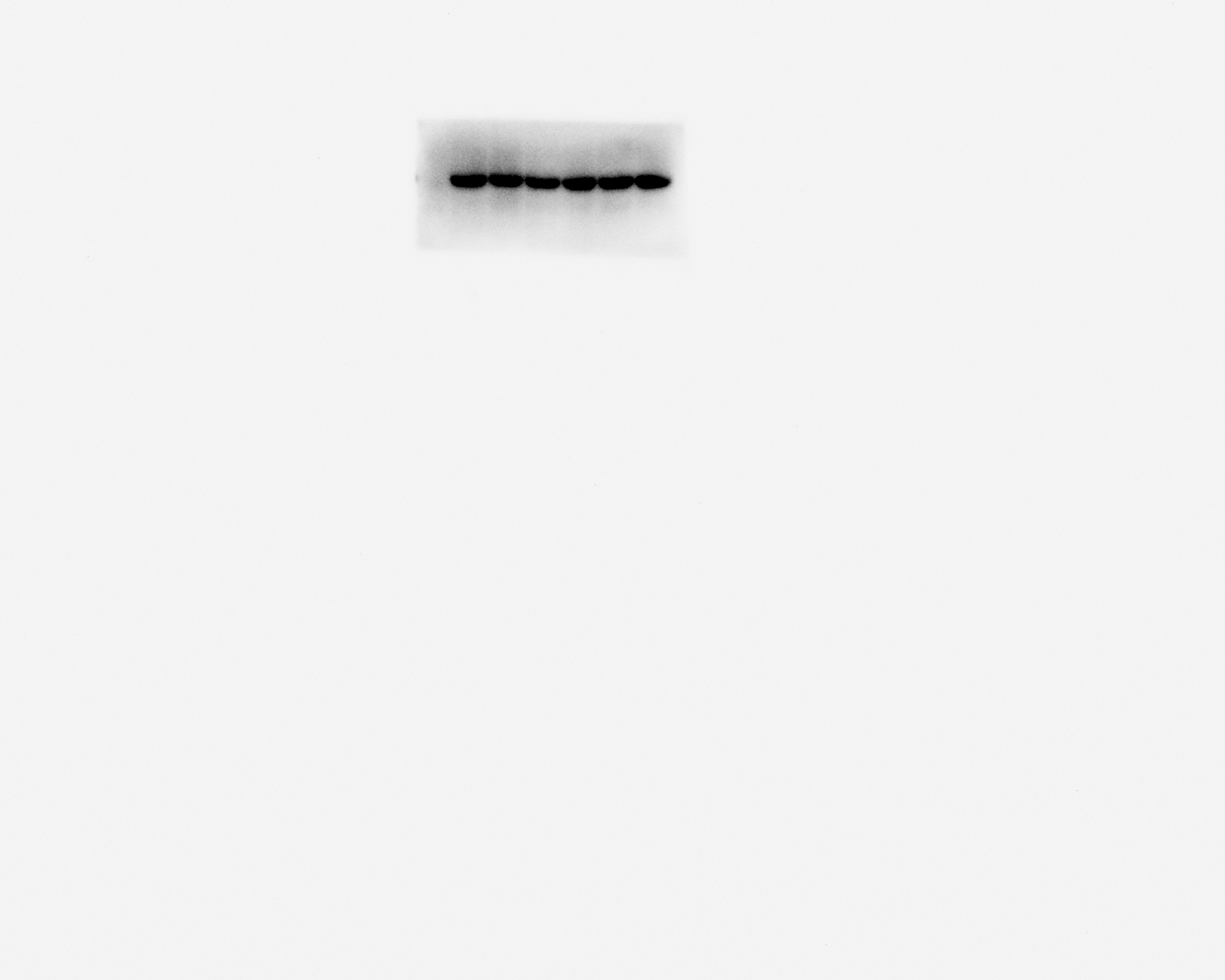

Supplement: Figure 5—figure supplement 1—source data 2. [file elife-89740-fig5-figsupp1-data2.zip › Figure 5-figure supplement 1-data 2/Figure 5-figure supplement 1—data 2-(B).tif]

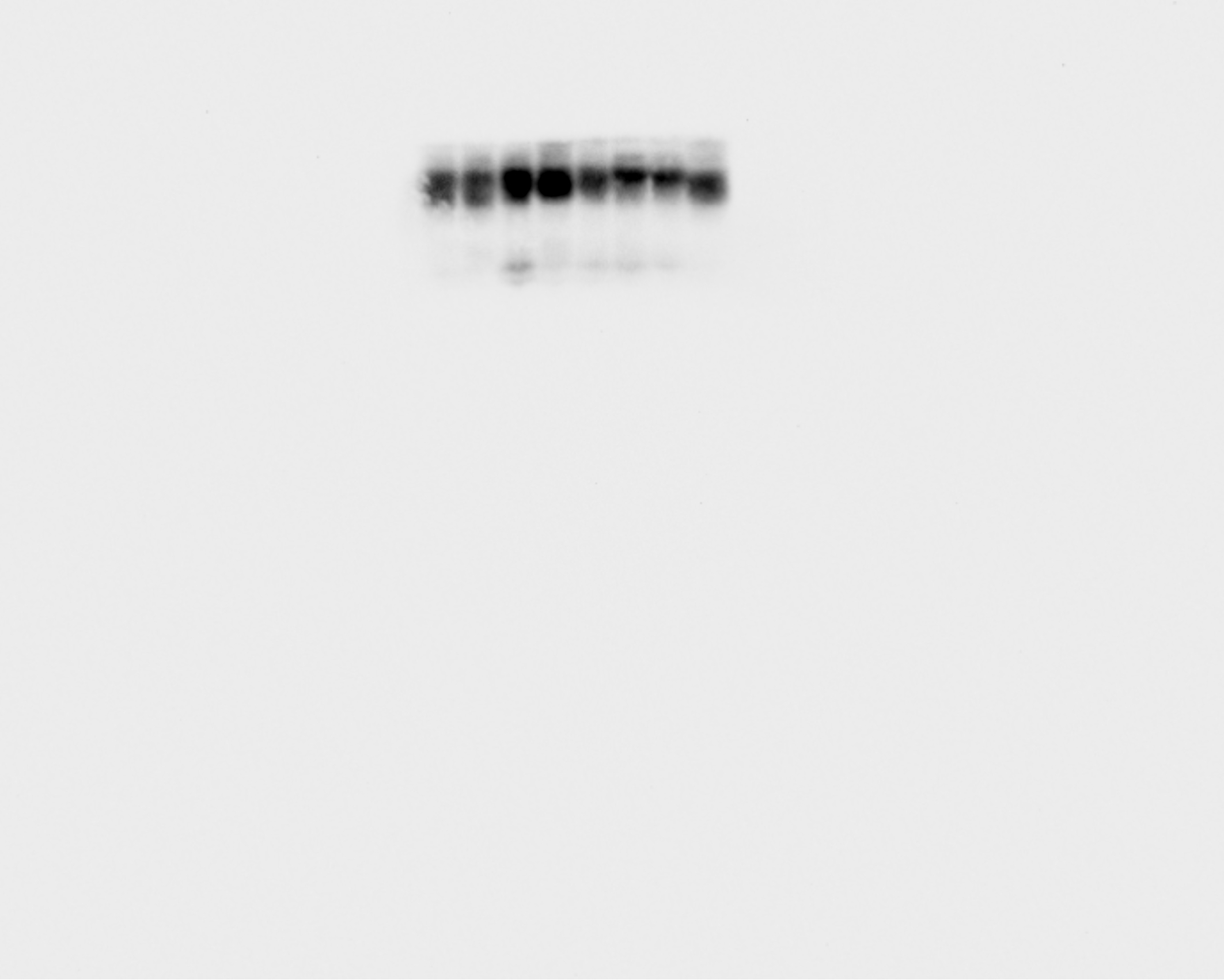

Supplement: Figure 5—figure supplement 2—source data 2. [file elife-89740-fig5-figsupp2-data2.zip › Figure 5-figure supplement 2-data 2/Figure 5-figure supplement 2—data 2-(A).jpg]

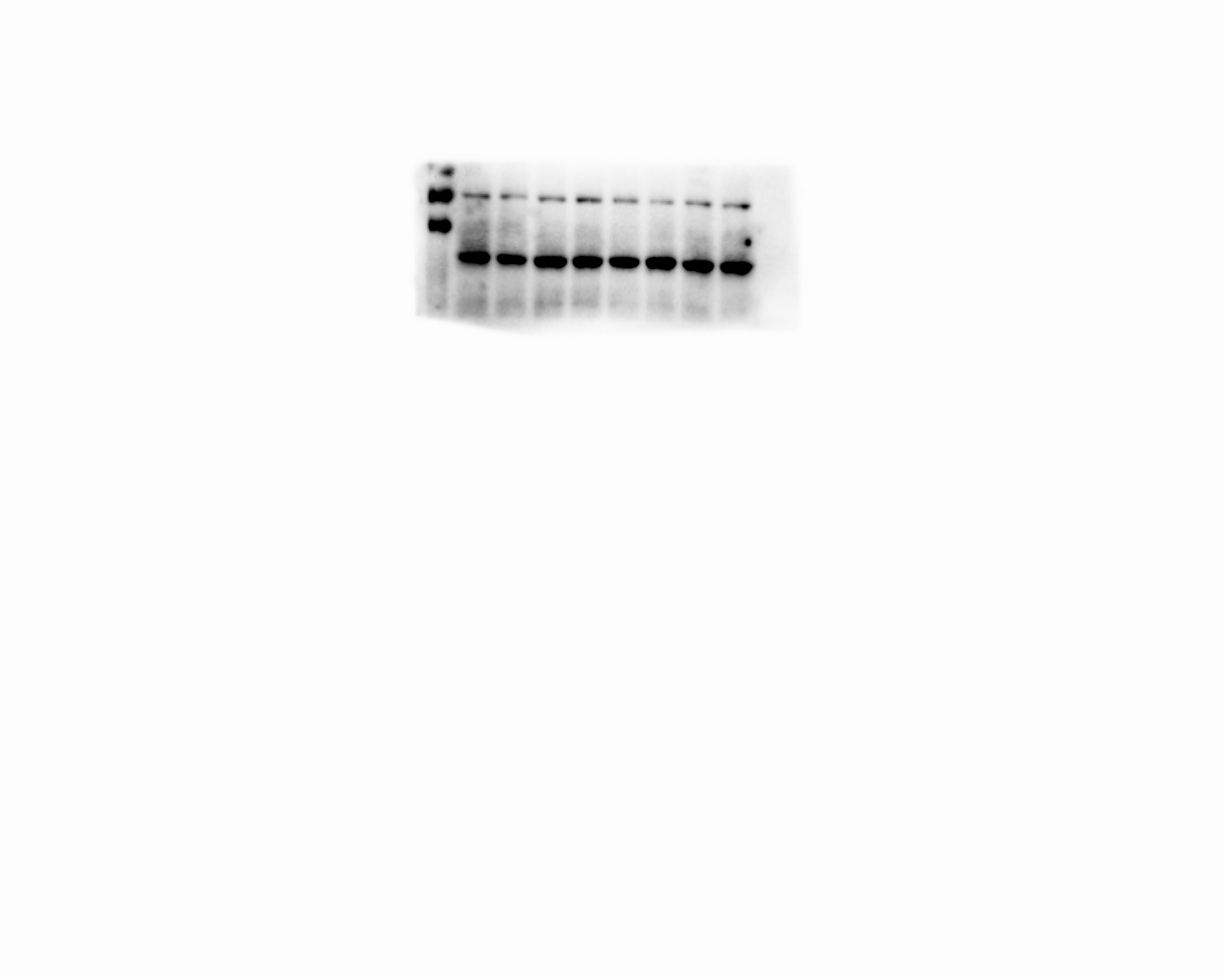

Supplement: Figure 5—figure supplement 2—source data 2. [file elife-89740-fig5-figsupp2-data2.zip › Figure 5-figure supplement 2-data 2/Figure 5-figure supplement 2—data 2-(B).tif]

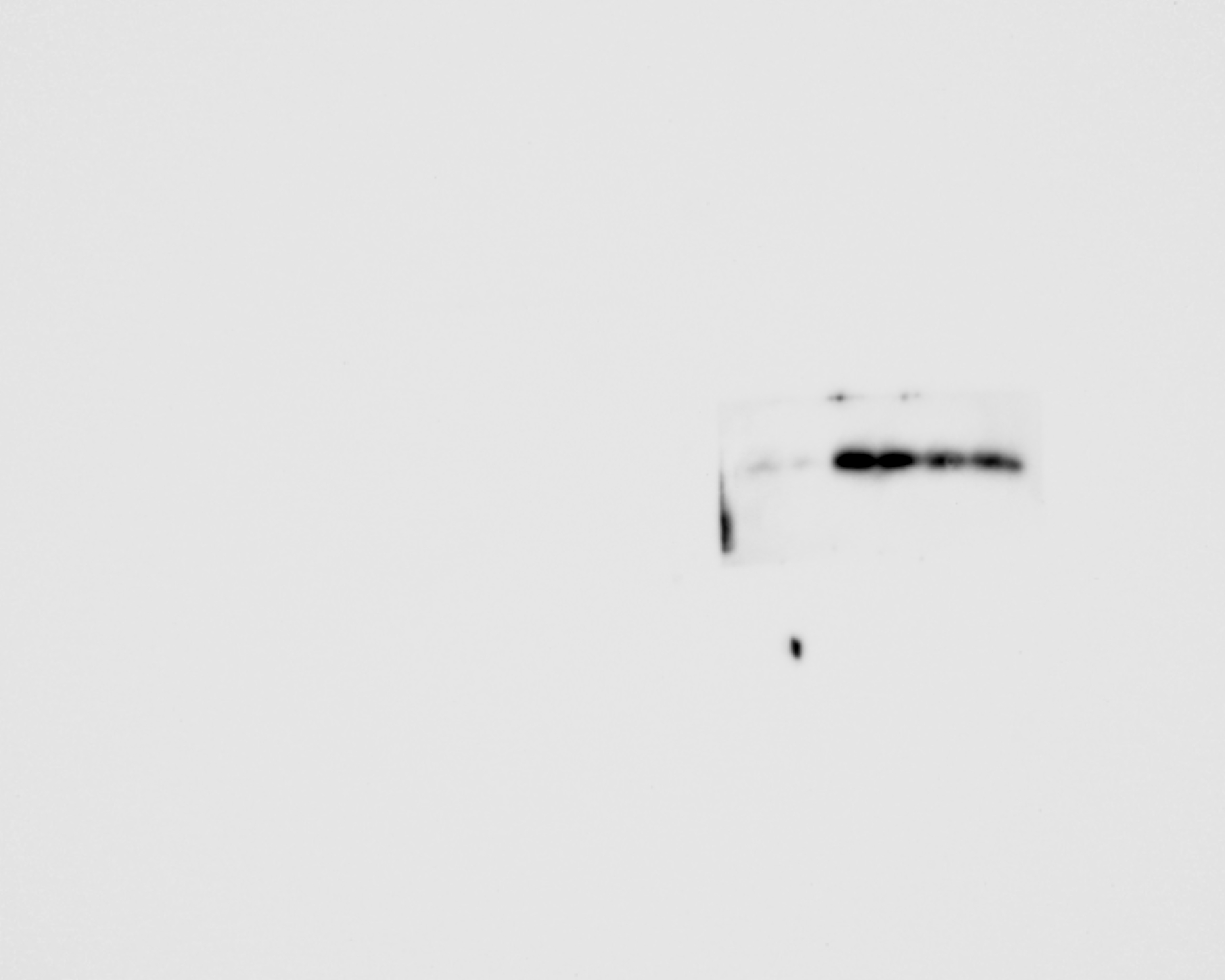

Supplement: Figure 5—figure supplement 4—source data 2. [file elife-89740-fig5-figsupp4-data2.zip › Figure 5-figure supplement 4- data 2/Figure 5-figure supplement 4—data 2-(A).jpg]

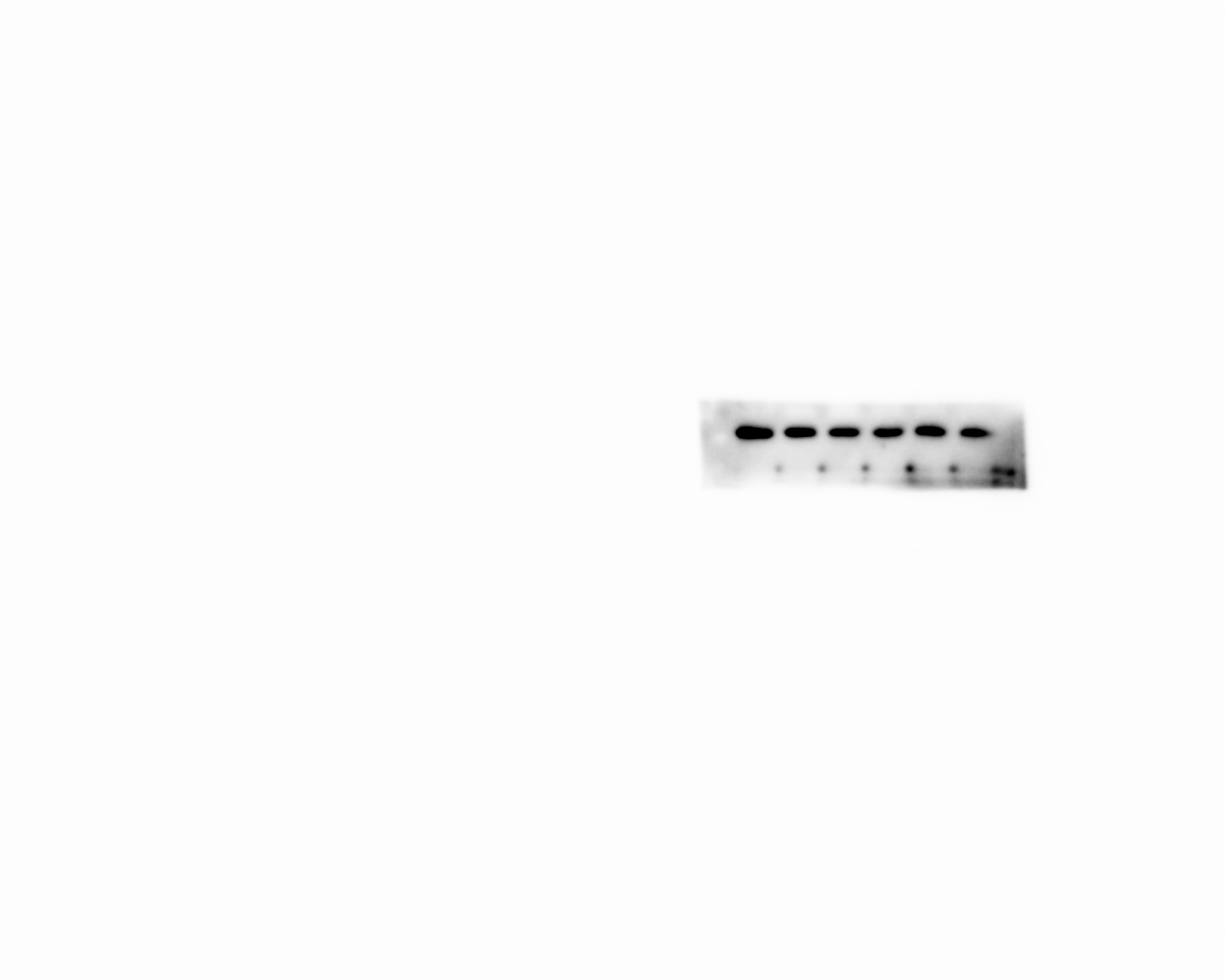

Supplement: Figure 5—figure supplement 4—source data 2. [file elife-89740-fig5-figsupp4-data2.zip › Figure 5-figure supplement 4- data 2/Figure 5-figure supplement 4—data 2-(B).jpg]

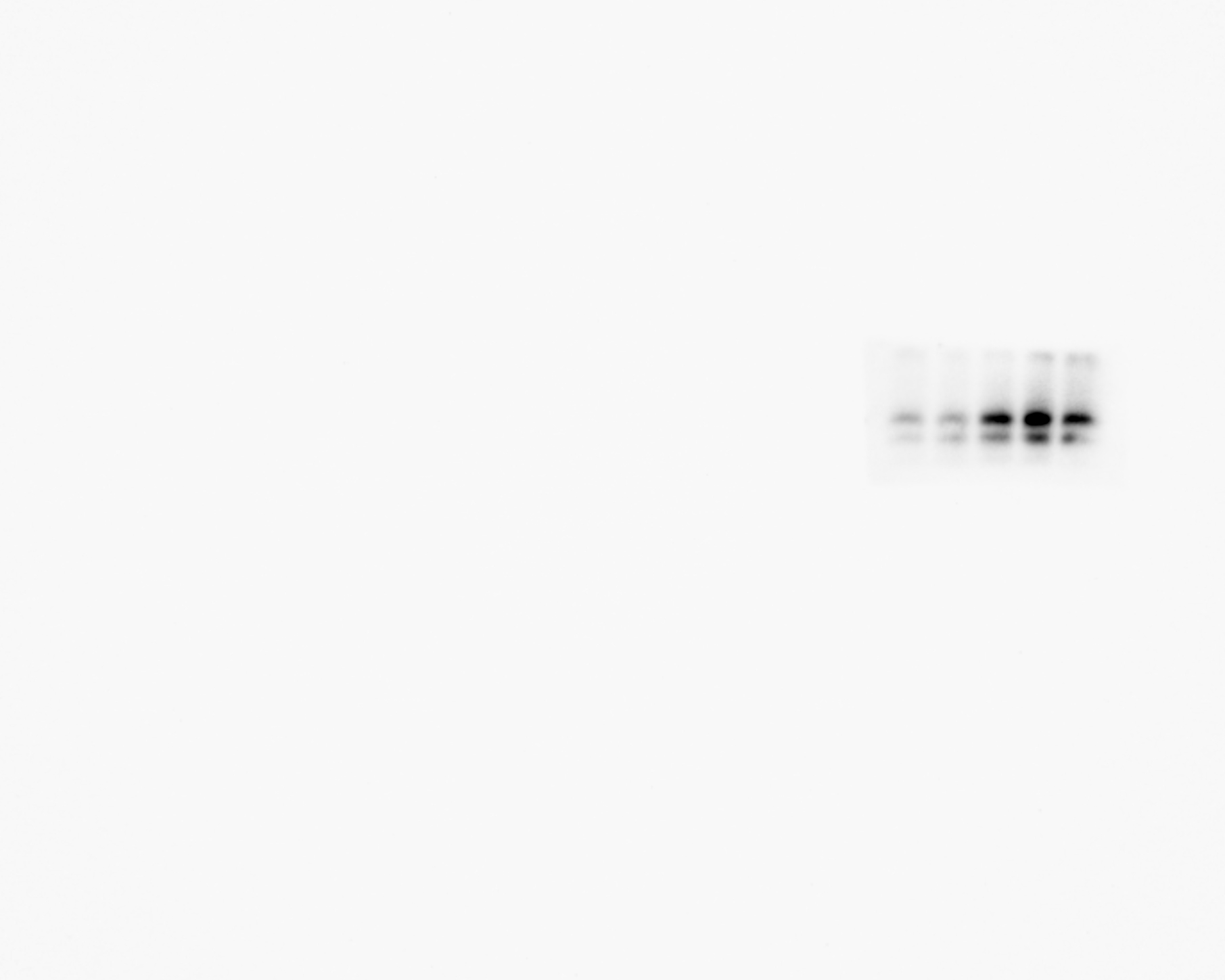

Supplement: Figure 5—figure supplement 4—source data 2. [file elife-89740-fig5-figsupp4-data2.zip › Figure 5-figure supplement 4- data 2/Figure 5-figure supplement 4—data 2-(C).jpg]

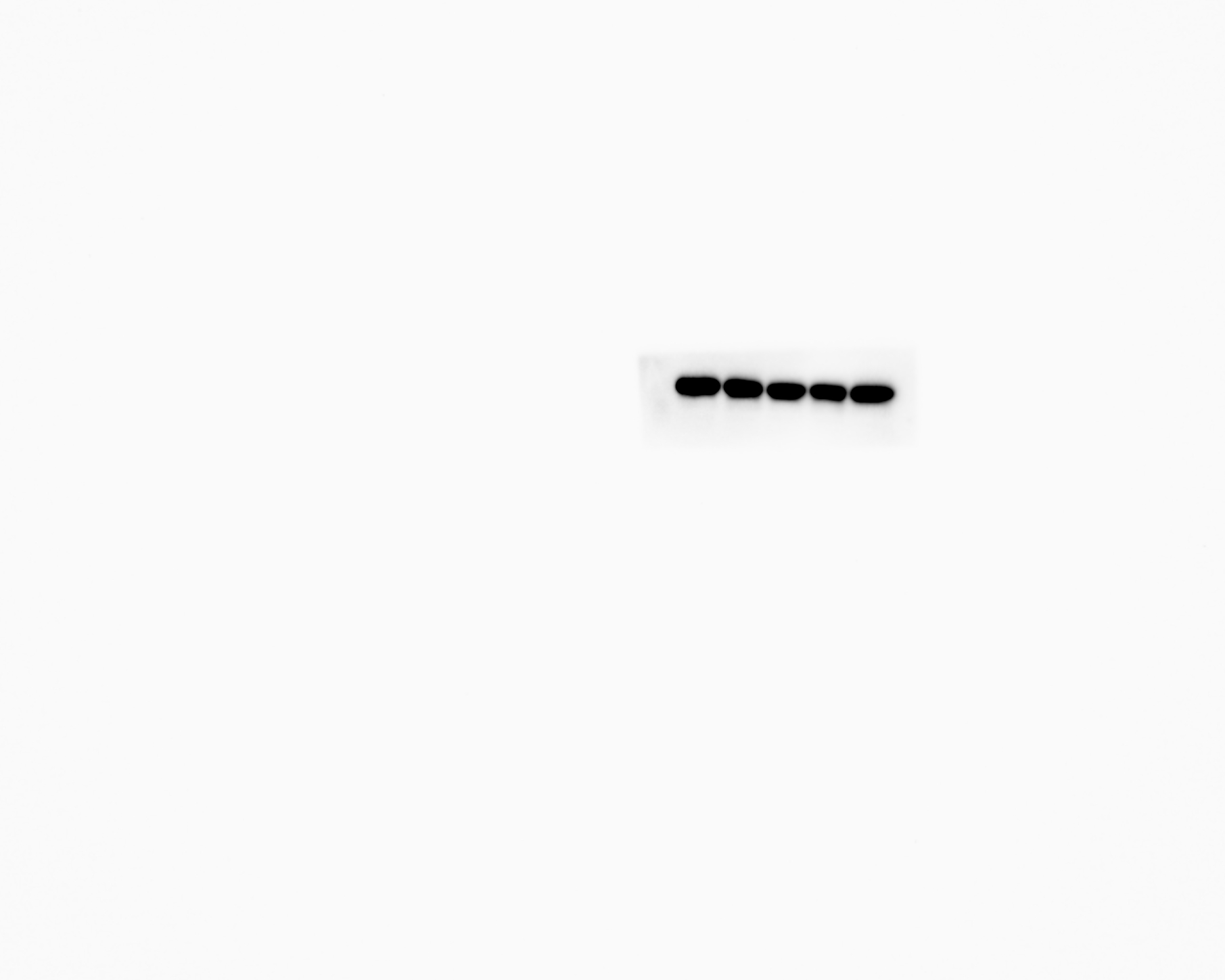

Supplement: Figure 5—figure supplement 4—source data 2. [file elife-89740-fig5-figsupp4-data2.zip › Figure 5-figure supplement 4- data 2/Figure 5-figure supplement 4—data 2-(D).jpg]

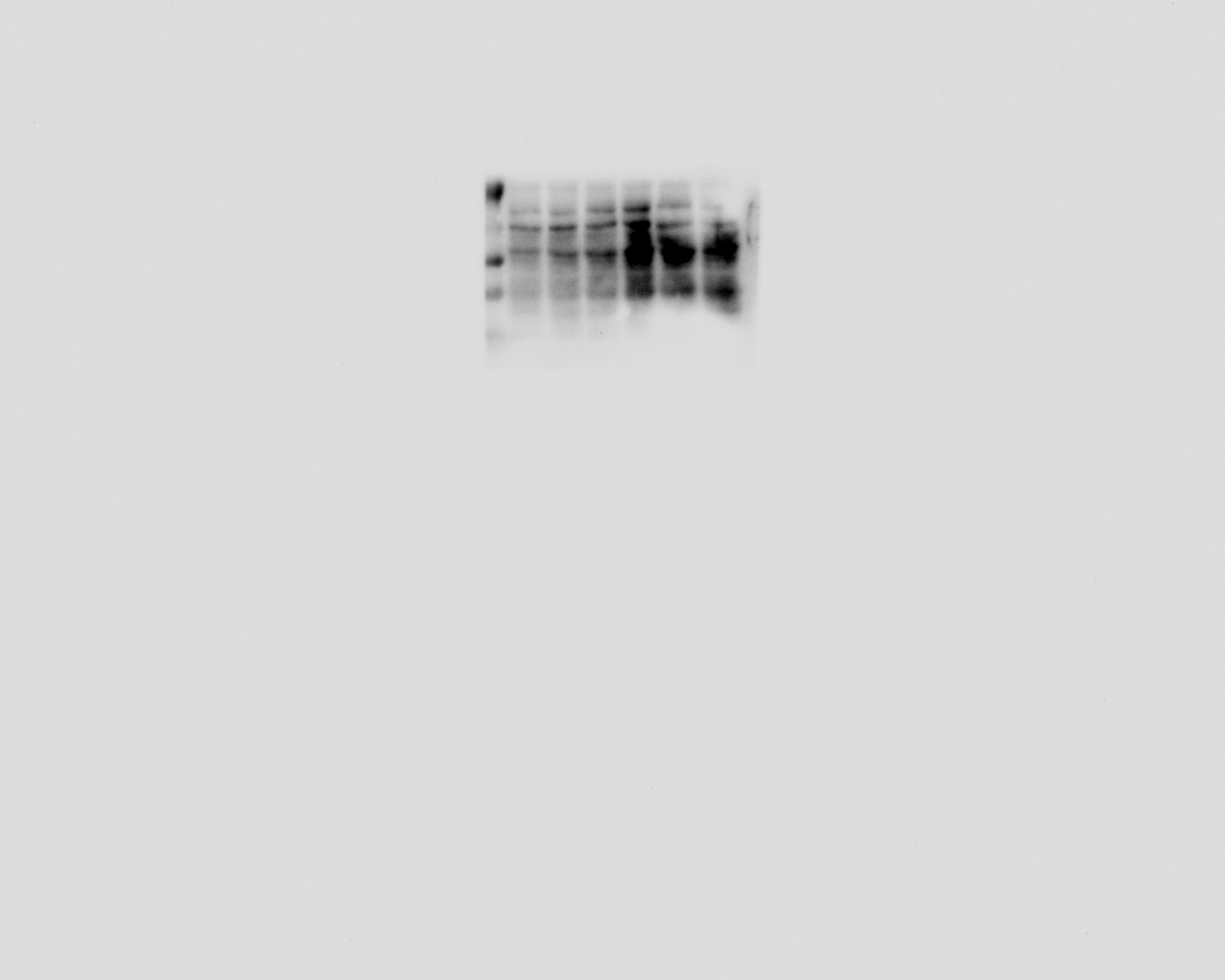

Supplement: Figure 6—figure supplement 1—source data 2. [file elife-89740-fig6-figsupp1-data2.zip › Figure 6-figure supplement 1-data 2/Figure 6-figure supplement 1—data 2-(A).tif]

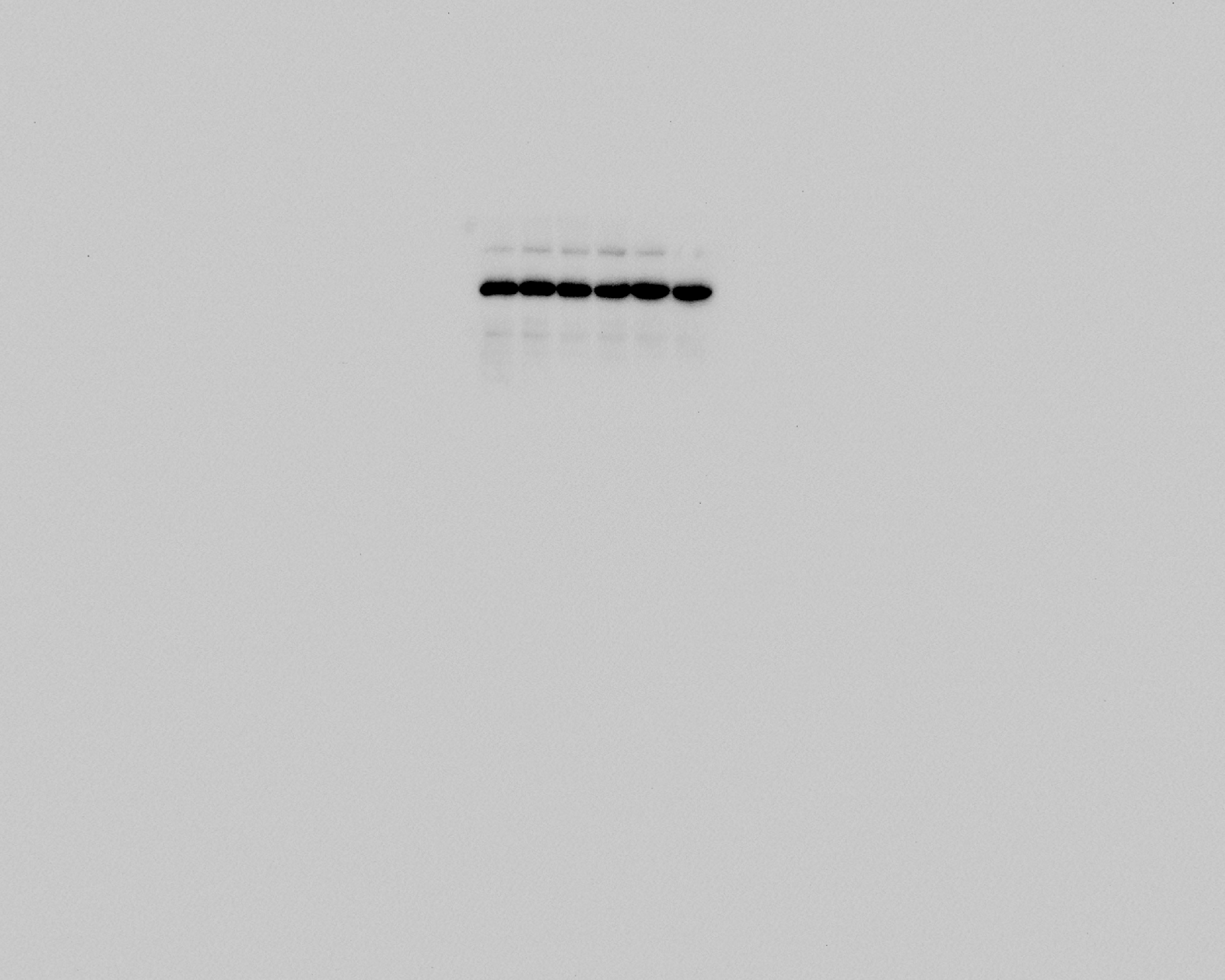

Supplement: Figure 6—figure supplement 1—source data 2. [file elife-89740-fig6-figsupp1-data2.zip › Figure 6-figure supplement 1-data 2/Figure 6-figure supplement 1—data 2-(B).tif]

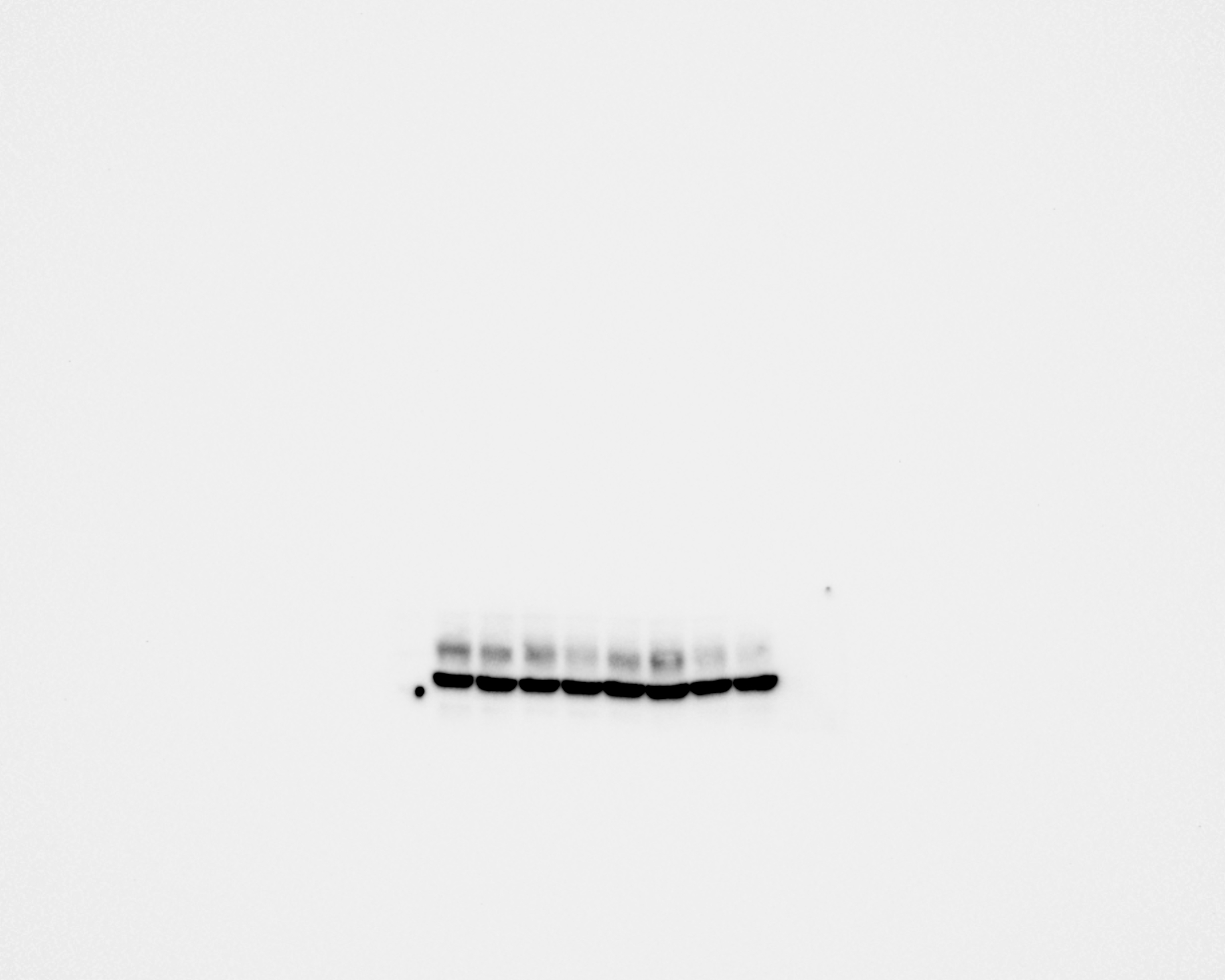

Supplement: Figure 7—source data 2. [file elife-89740-fig7-data2.zip › Figure 7-data 2/Figure 7—data 2-(A).jpg]

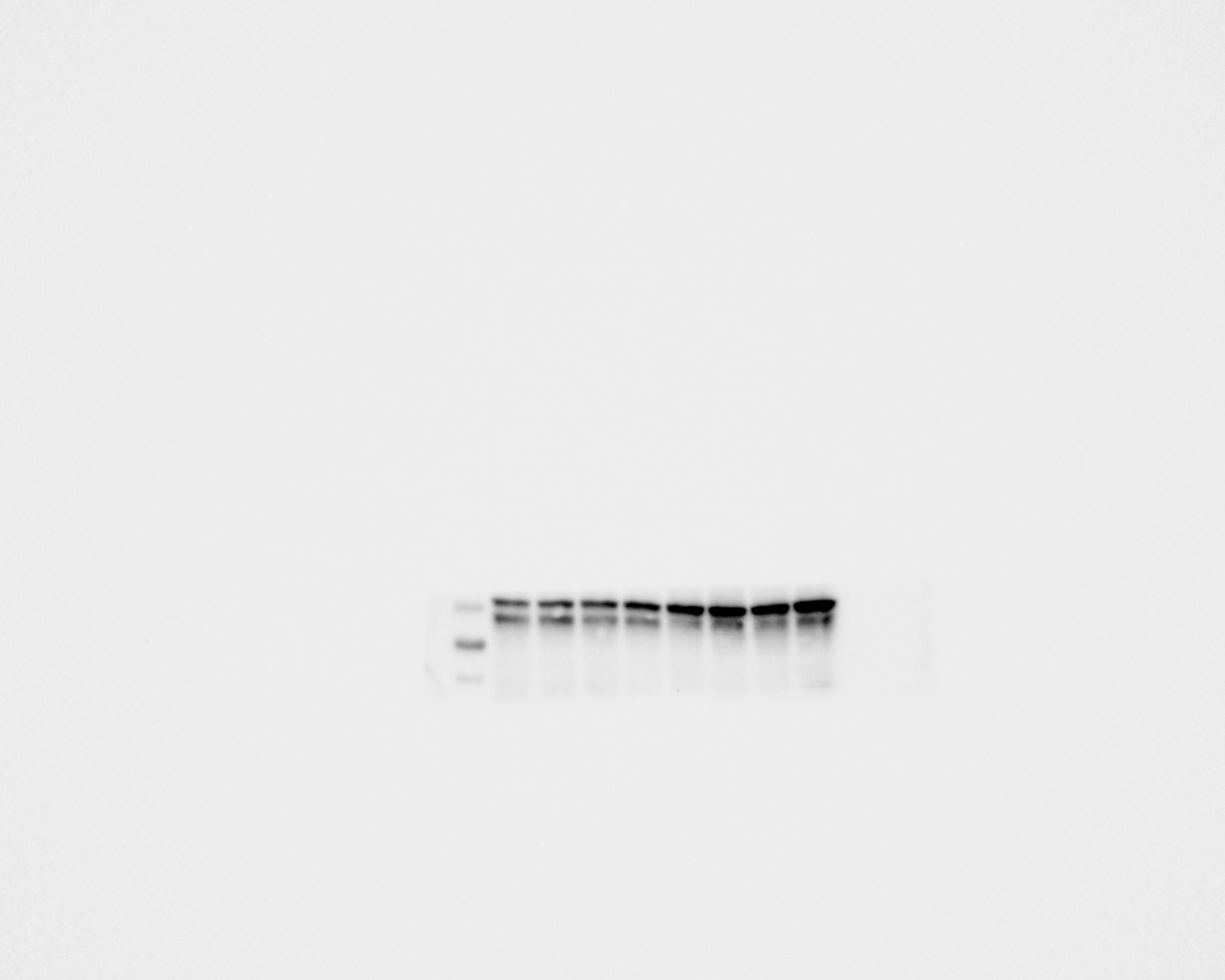

Supplement: Figure 7—source data 2. [file elife-89740-fig7-data2.zip › Figure 7-data 2/Figure 7—data 2-(B).jpg]

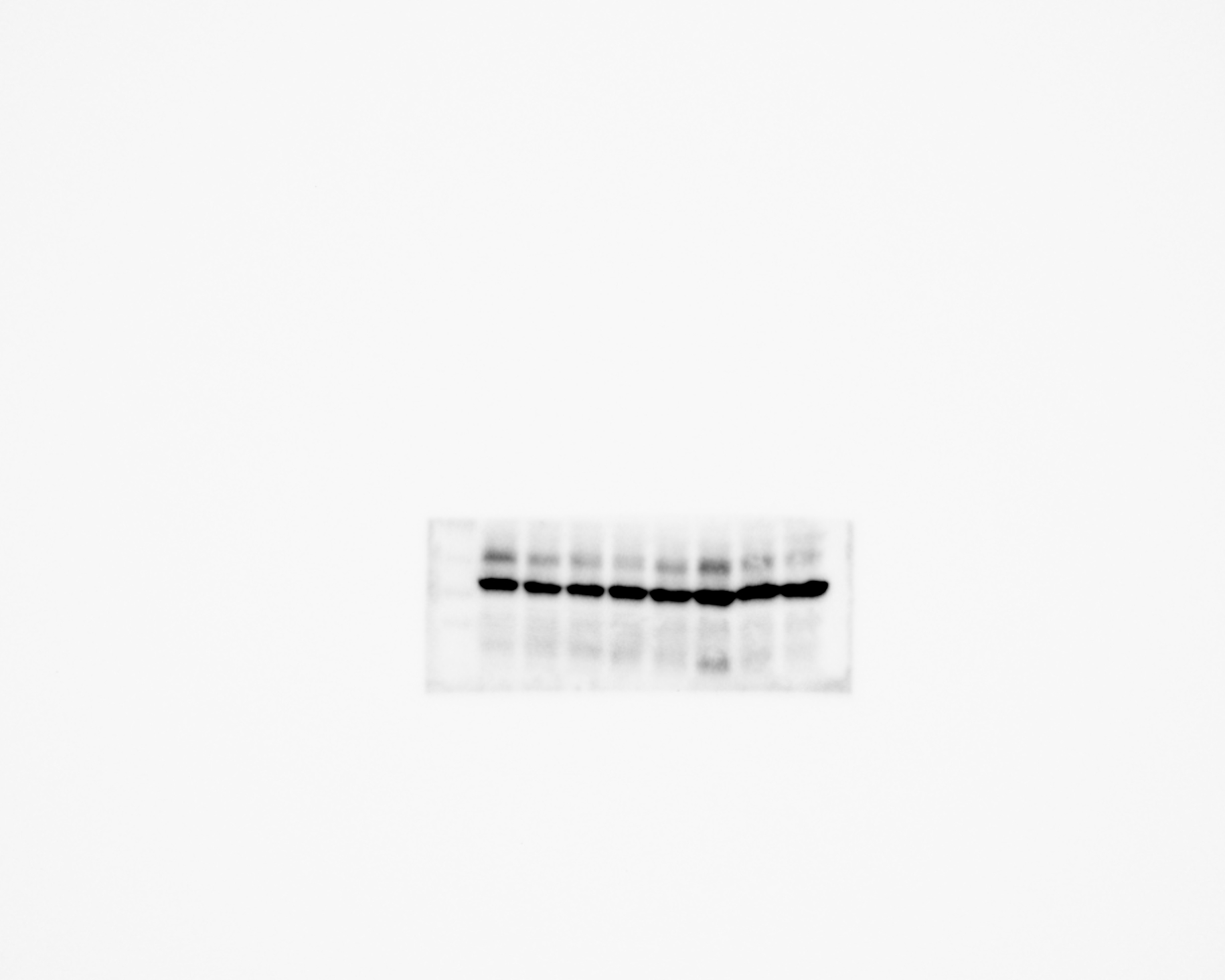

Supplement: Figure 8—source data 2. [file elife-89740-fig8-data2.zip › Figure 8-data 2/Figure 8—data 2-(A).jpg]

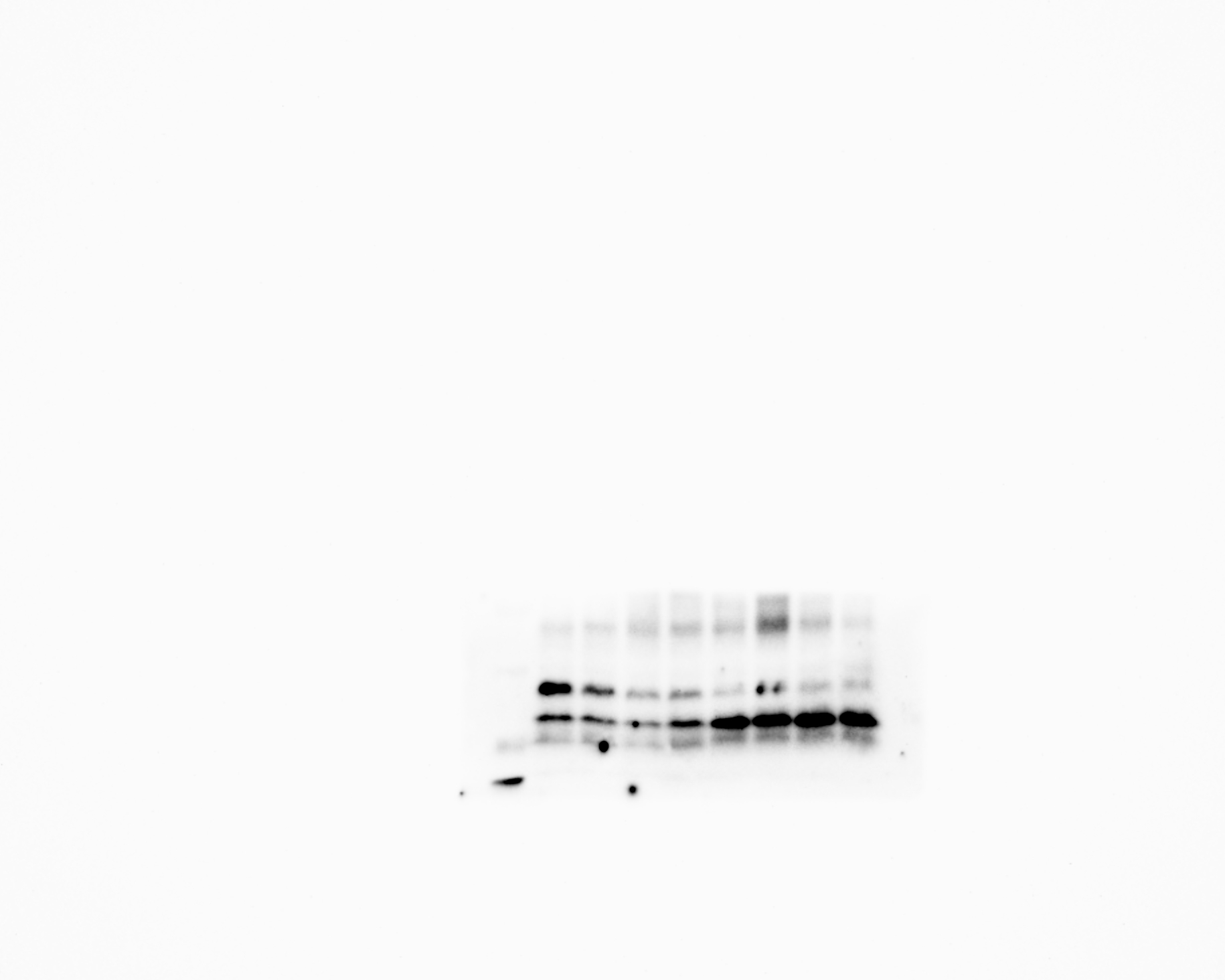

Supplement: Figure 8—source data 2. [file elife-89740-fig8-data2.zip › Figure 8-data 2/Figure 8—data 2-(B).jpg]

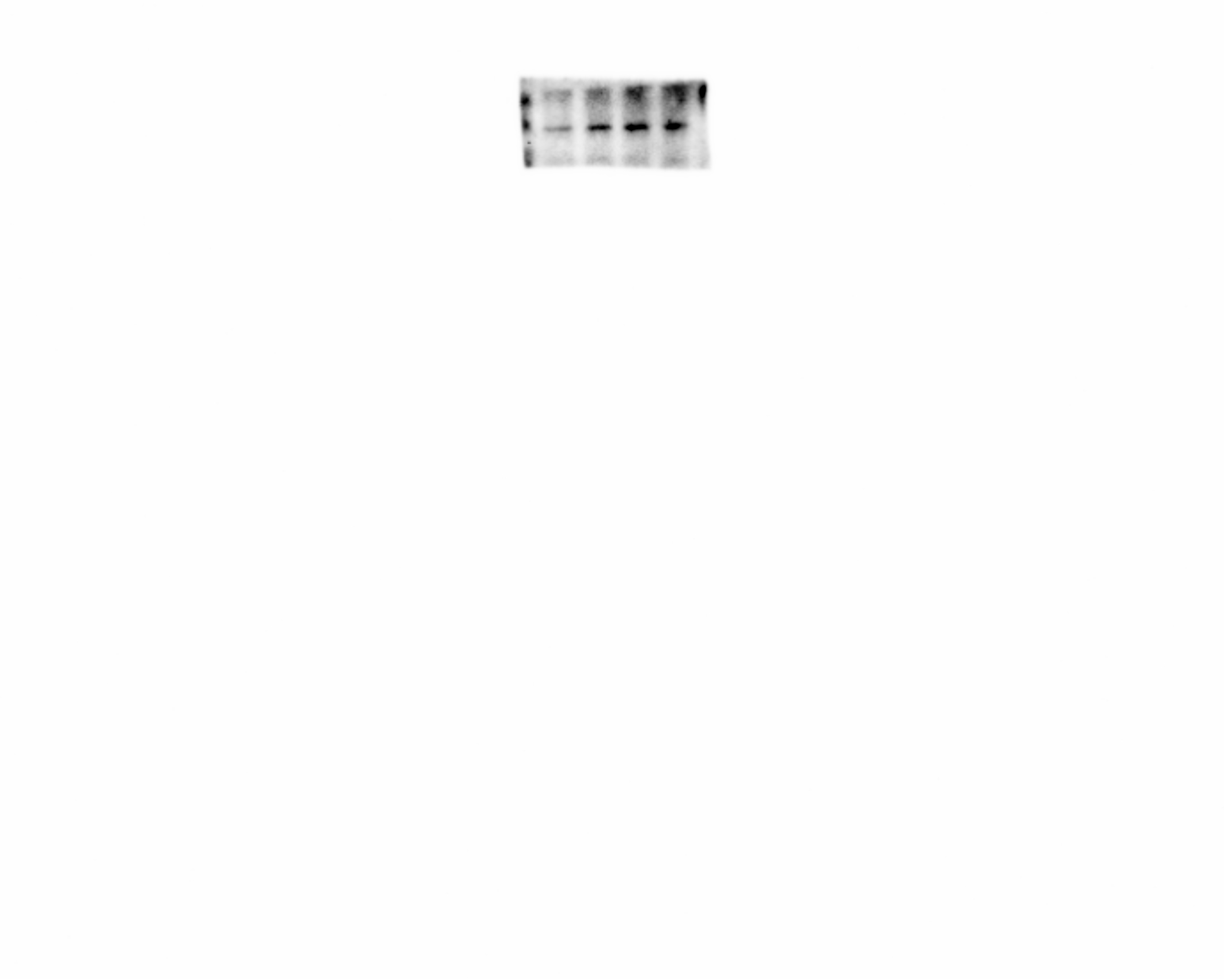

Supplement: Figure 9—source data 2. [file elife-89740-fig9-data2.zip › Figure 9-data 2/Figure 9—data 2-(A).jpg]

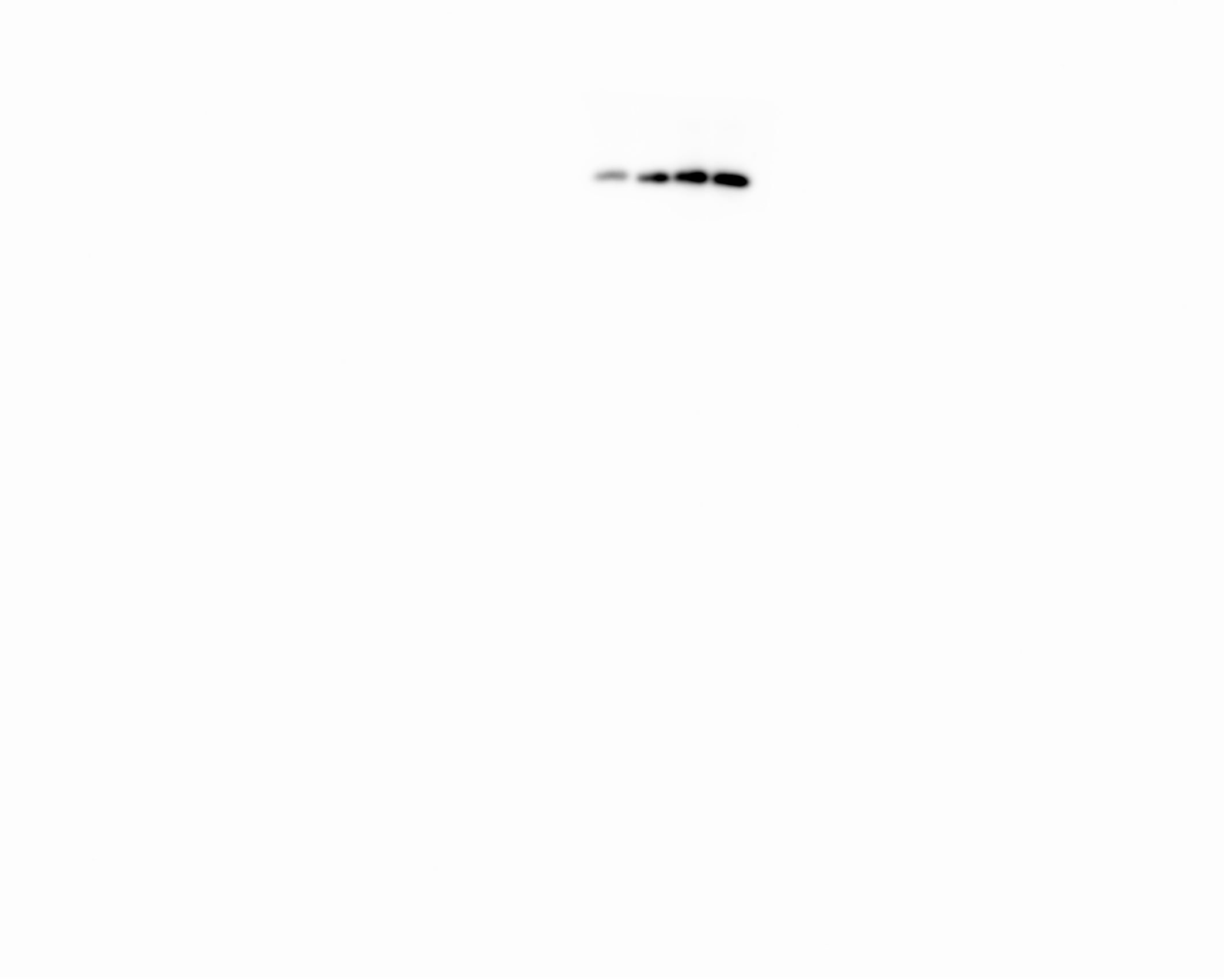

Supplement: Figure 9—source data 2. [file elife-89740-fig9-data2.zip › Figure 9-data 2/Figure 9—data 2-(B).jpg]

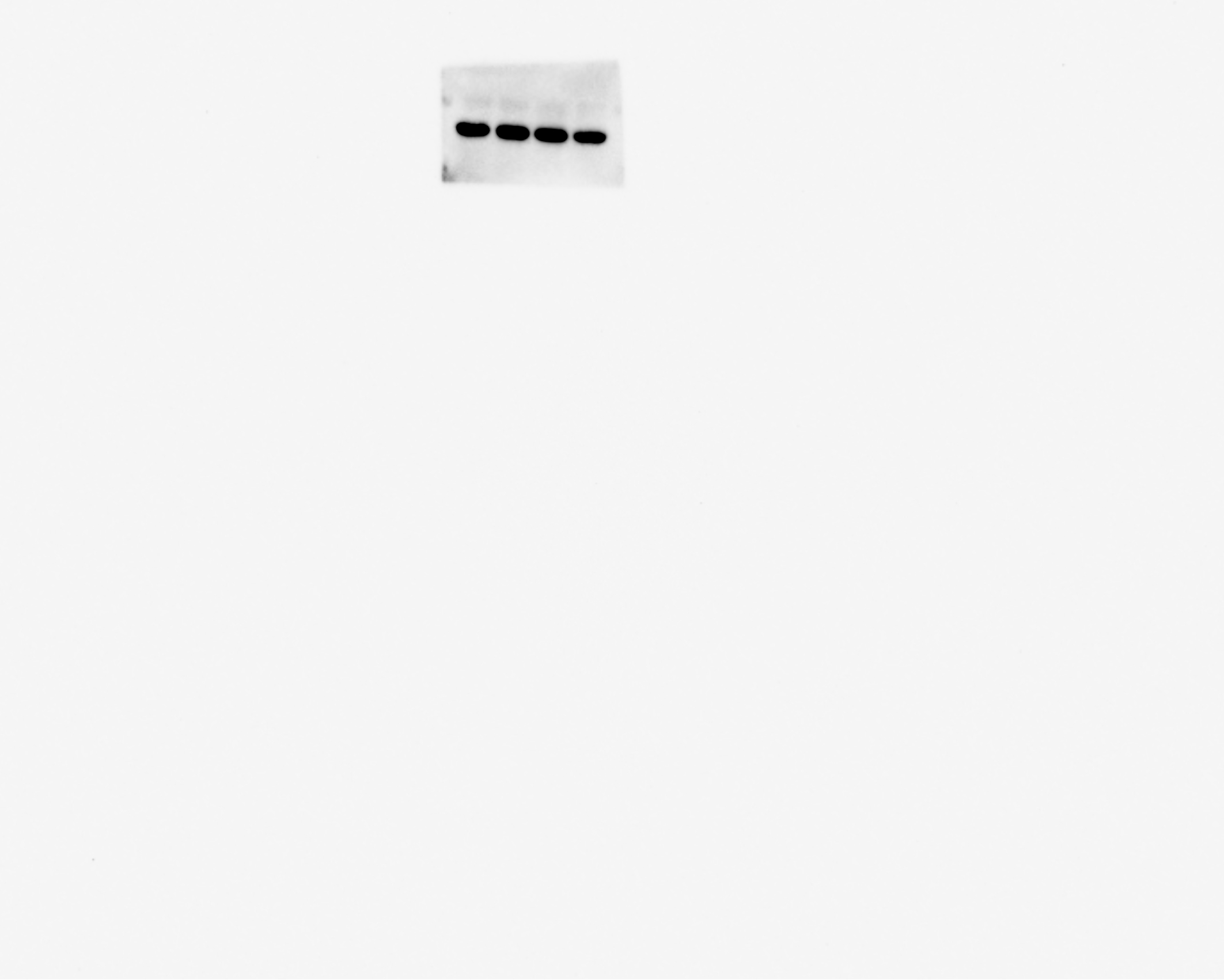

Supplement: Figure 9—source data 2. [file elife-89740-fig9-data2.zip › Figure 9-data 2/Figure 9—data 2-(C).jpg]

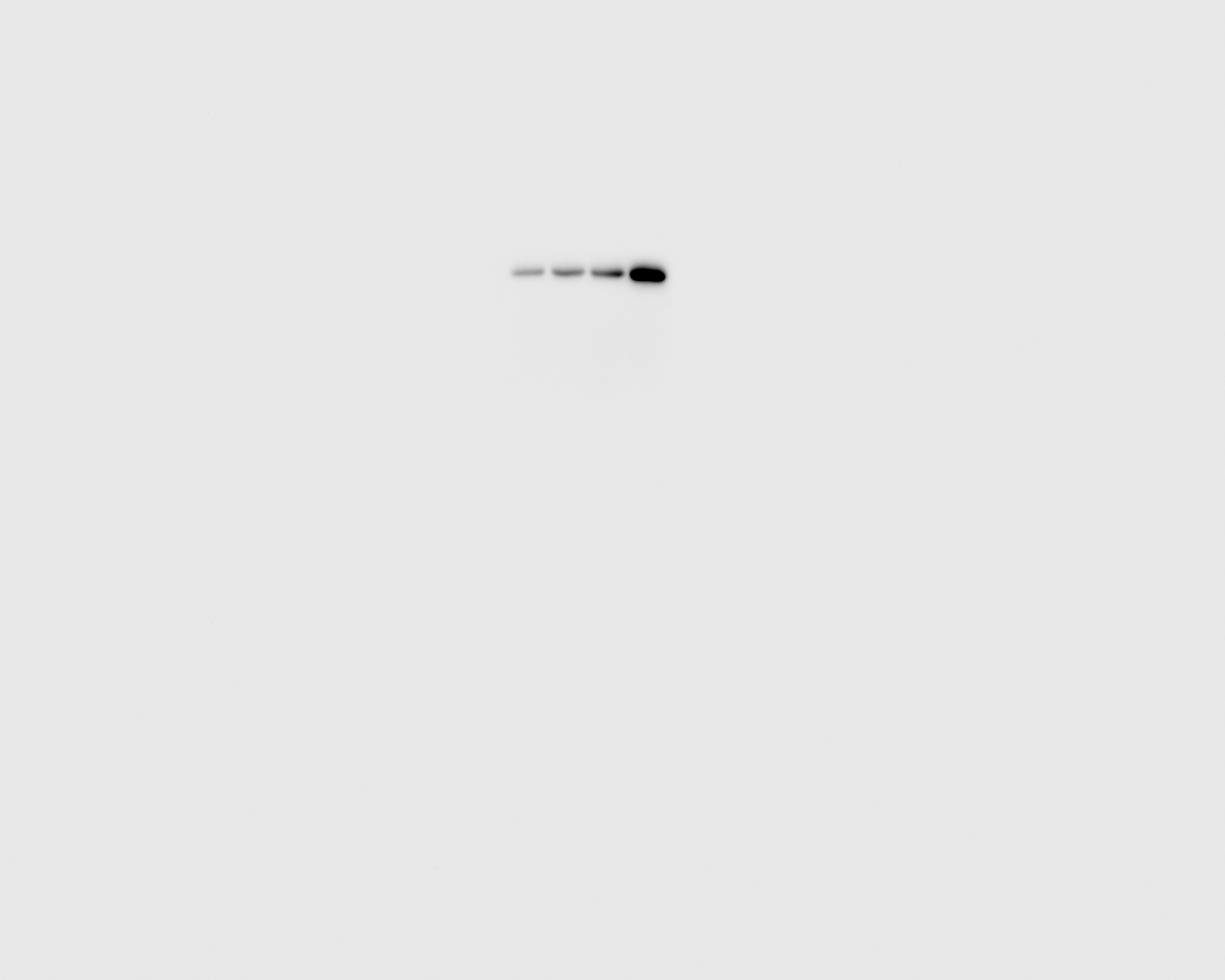

Supplement: Figure 9—figure supplement 1—source data 2. [file elife-89740-fig9-figsupp1-data2.zip › Figure 9-figure supplement 1-data 2/Figure 9-figure supplement 1—data 2-(A).tif]

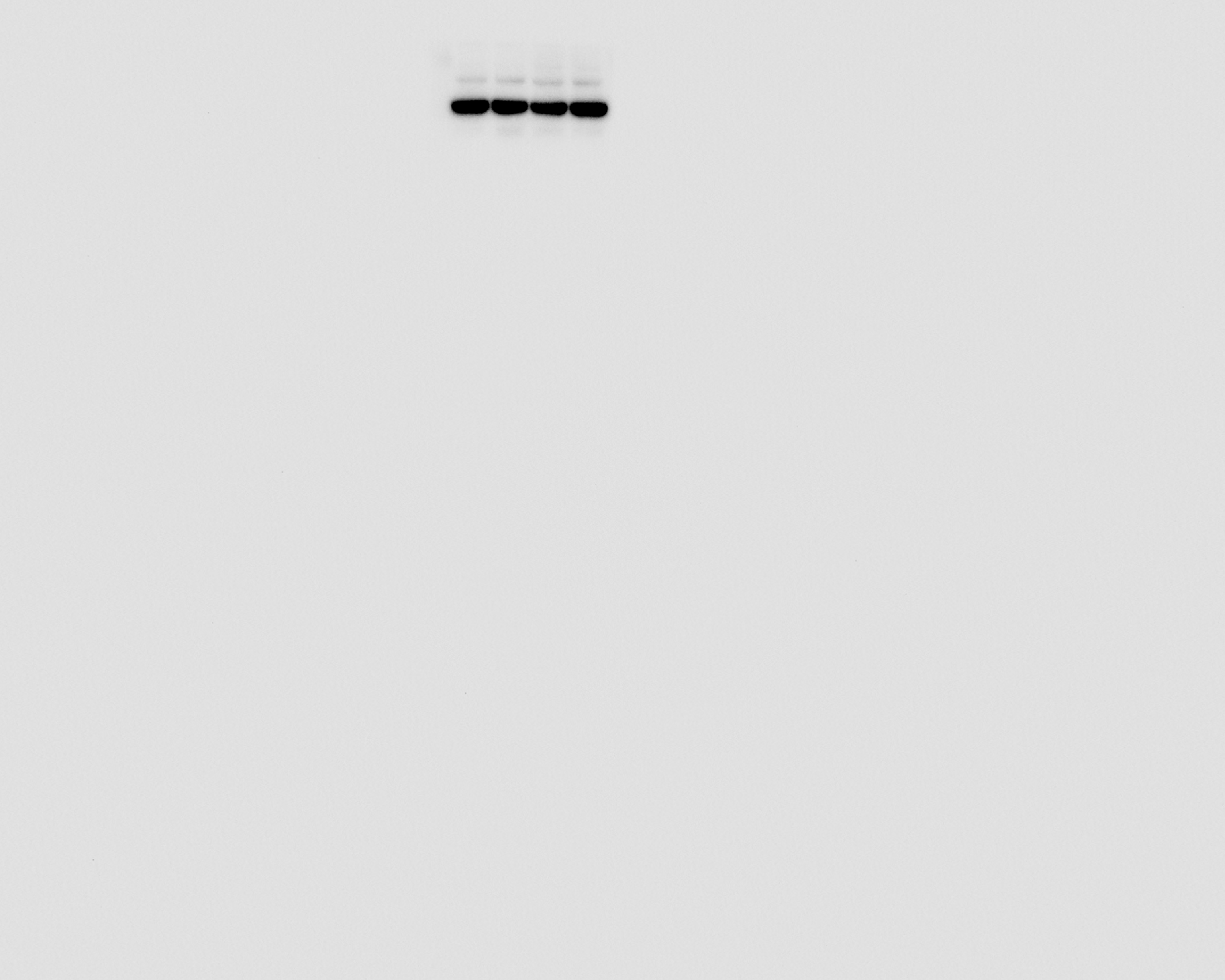

Supplement: Figure 9—figure supplement 1—source data 2. [file elife-89740-fig9-figsupp1-data2.zip › Figure 9-figure supplement 1-data 2/Figure 9-figure supplement 1—data 2-(B).tif]

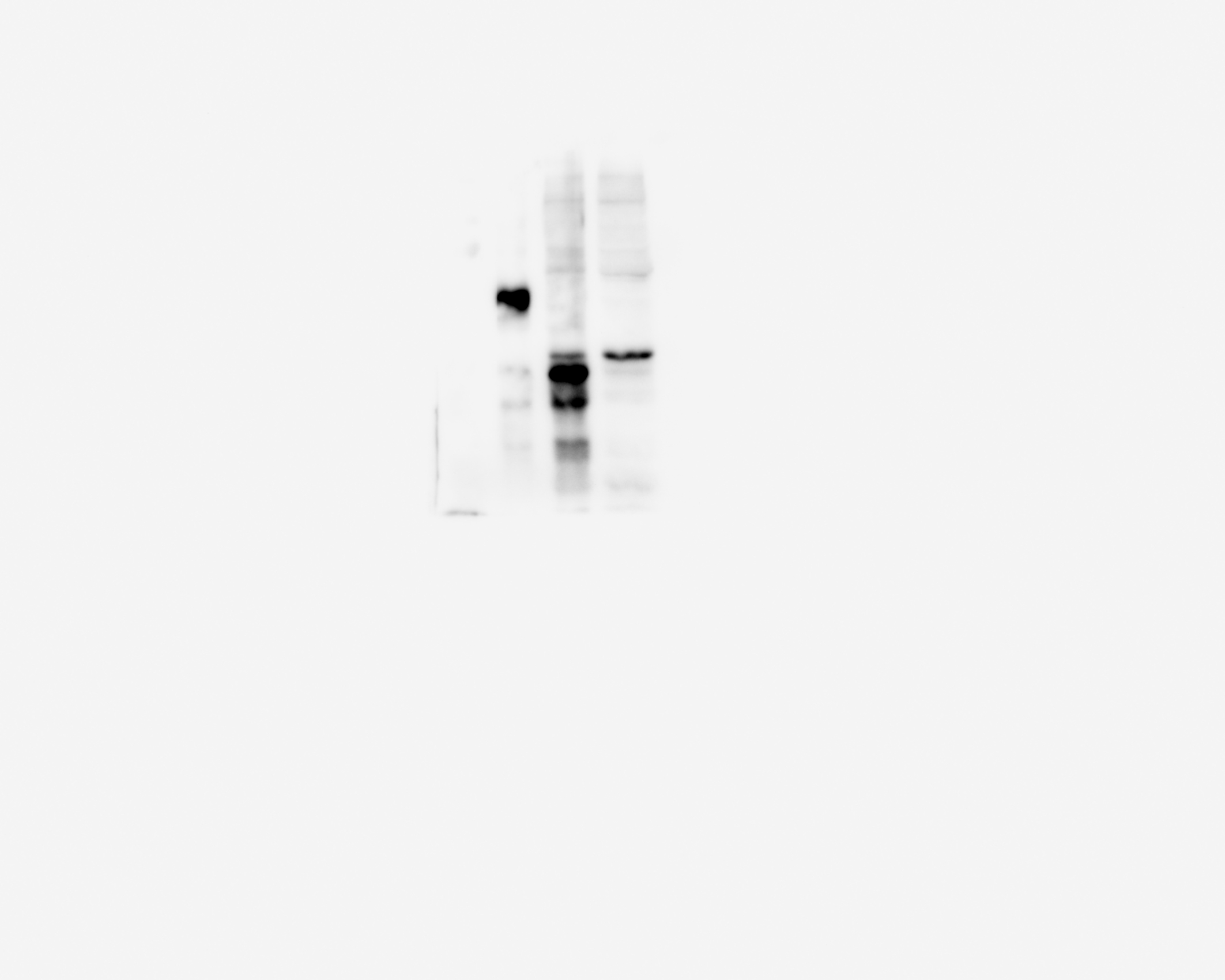

Supplement: Figure 10—source data 2. [file elife-89740-fig10-data2.zip › Figure 10-data2/Figure 10—data 2-(A).jpg]

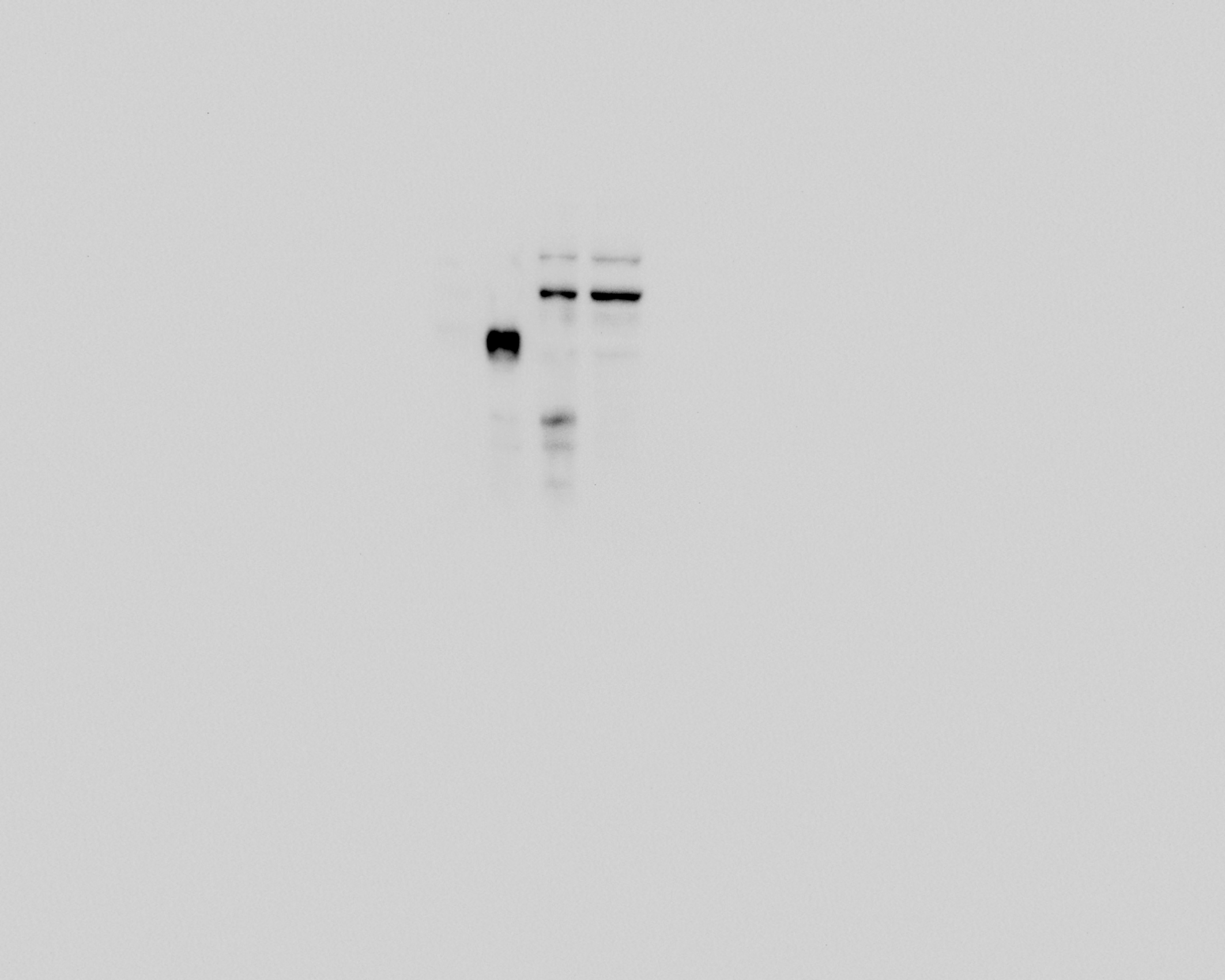

Supplement: Figure 10—source data 2. [file elife-89740-fig10-data2.zip › Figure 10-data2/Figure 10—data 2-(B).jpg]

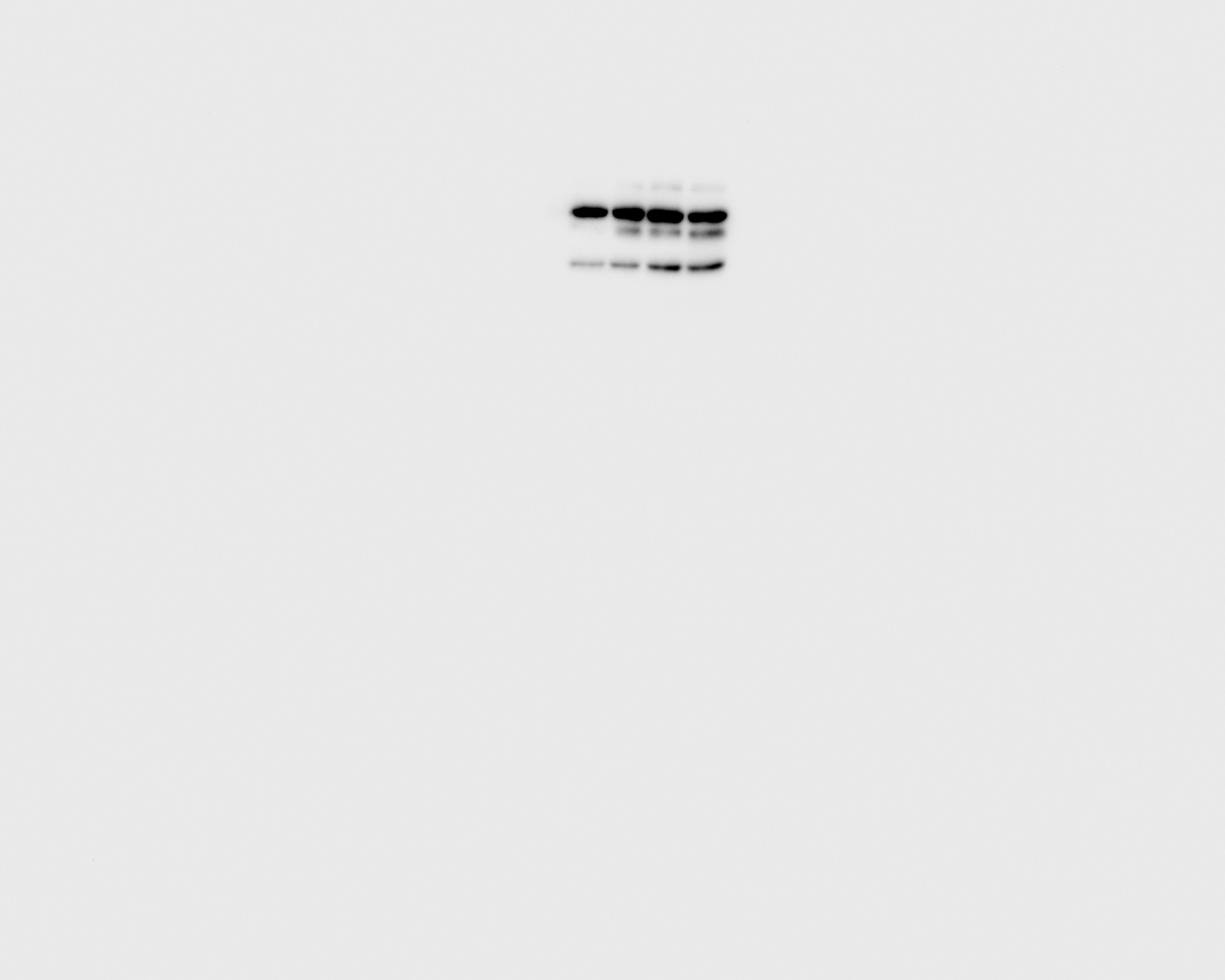

Supplement: Figure 10—source data 2. [file elife-89740-fig10-data2.zip › Figure 10-data2/Figure 10—data 2-(C).jpg]

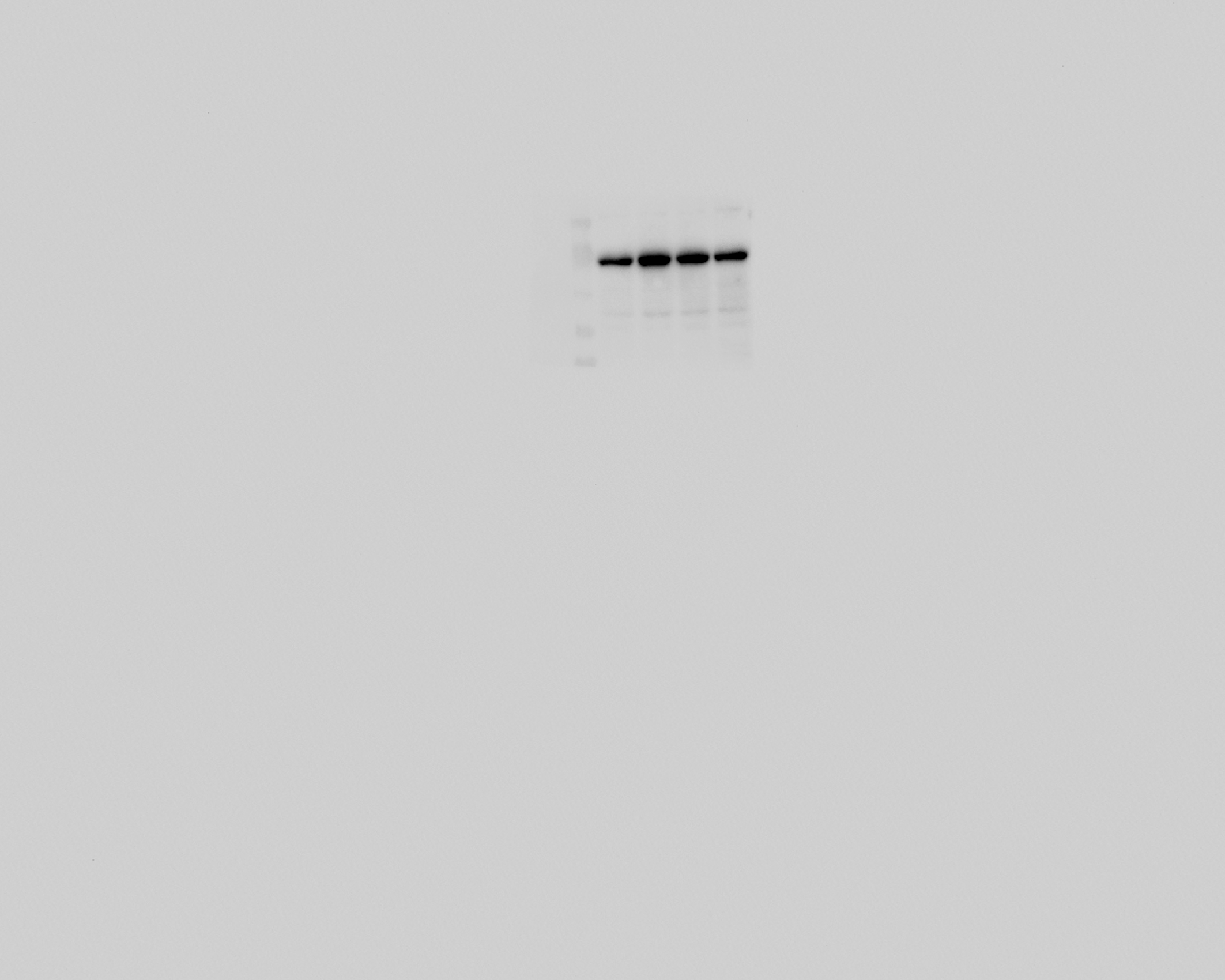

Supplement: Figure 10—source data 2. [file elife-89740-fig10-data2.zip › Figure 10-data2/Figure 10—data 2-(D).jpg]

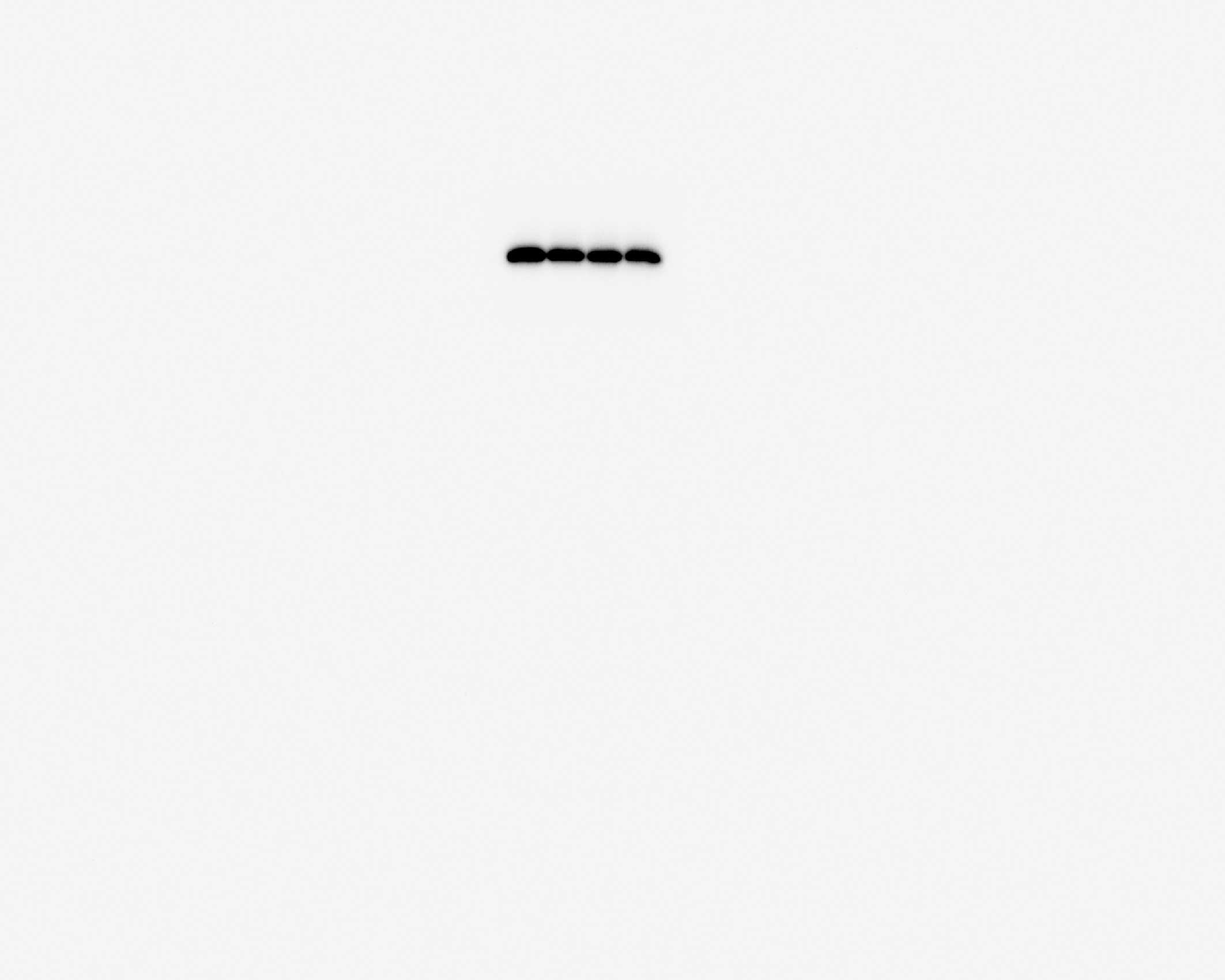

Supplement: Figure 10—source data 2. [file elife-89740-fig10-data2.zip › Figure 10-data2/Figure 10—data 2-(E).jpg]

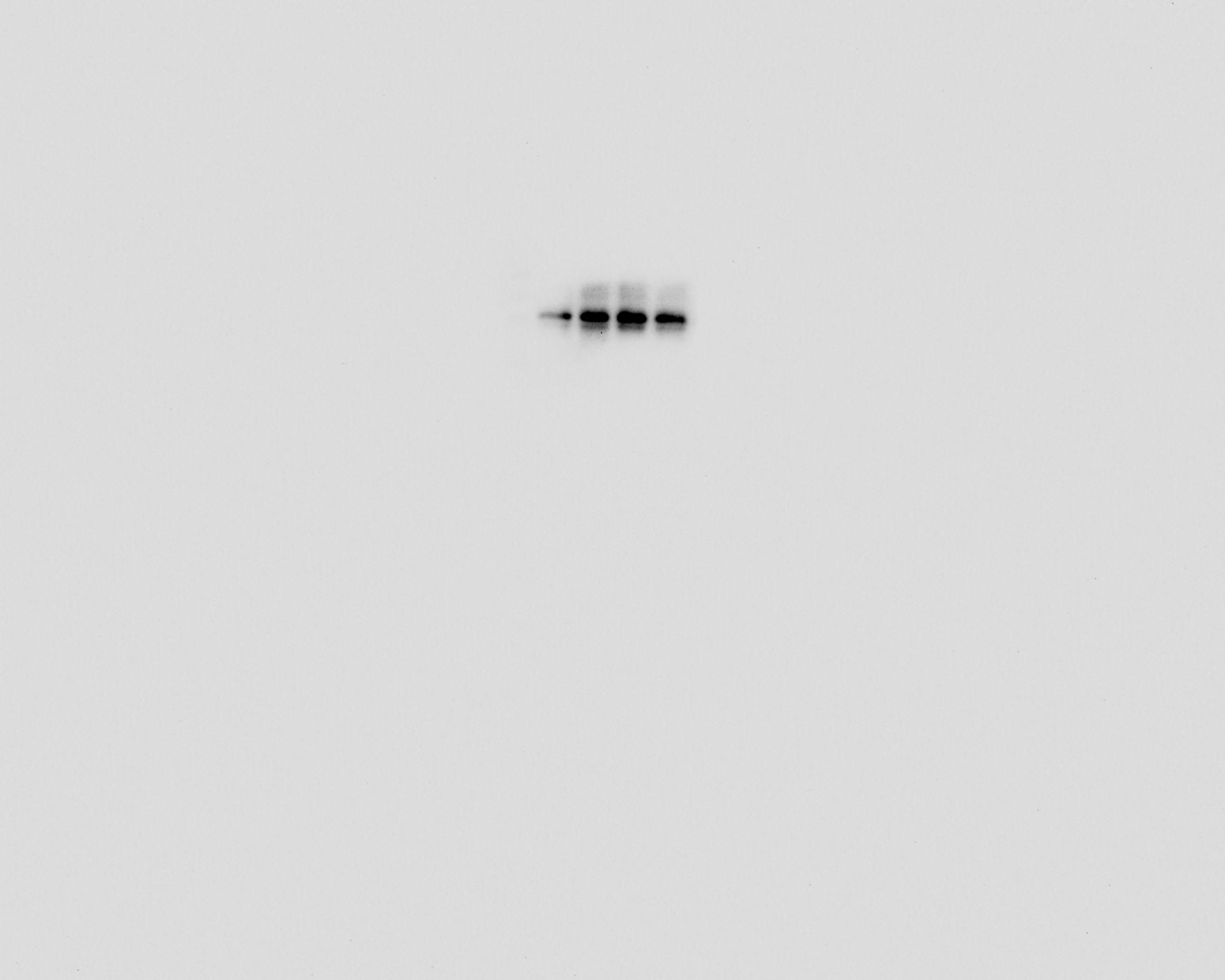

Supplement: Figure 10—source data 2. [file elife-89740-fig10-data2.zip › Figure 10-data2/Figure 10—data 2-(F).jpg]

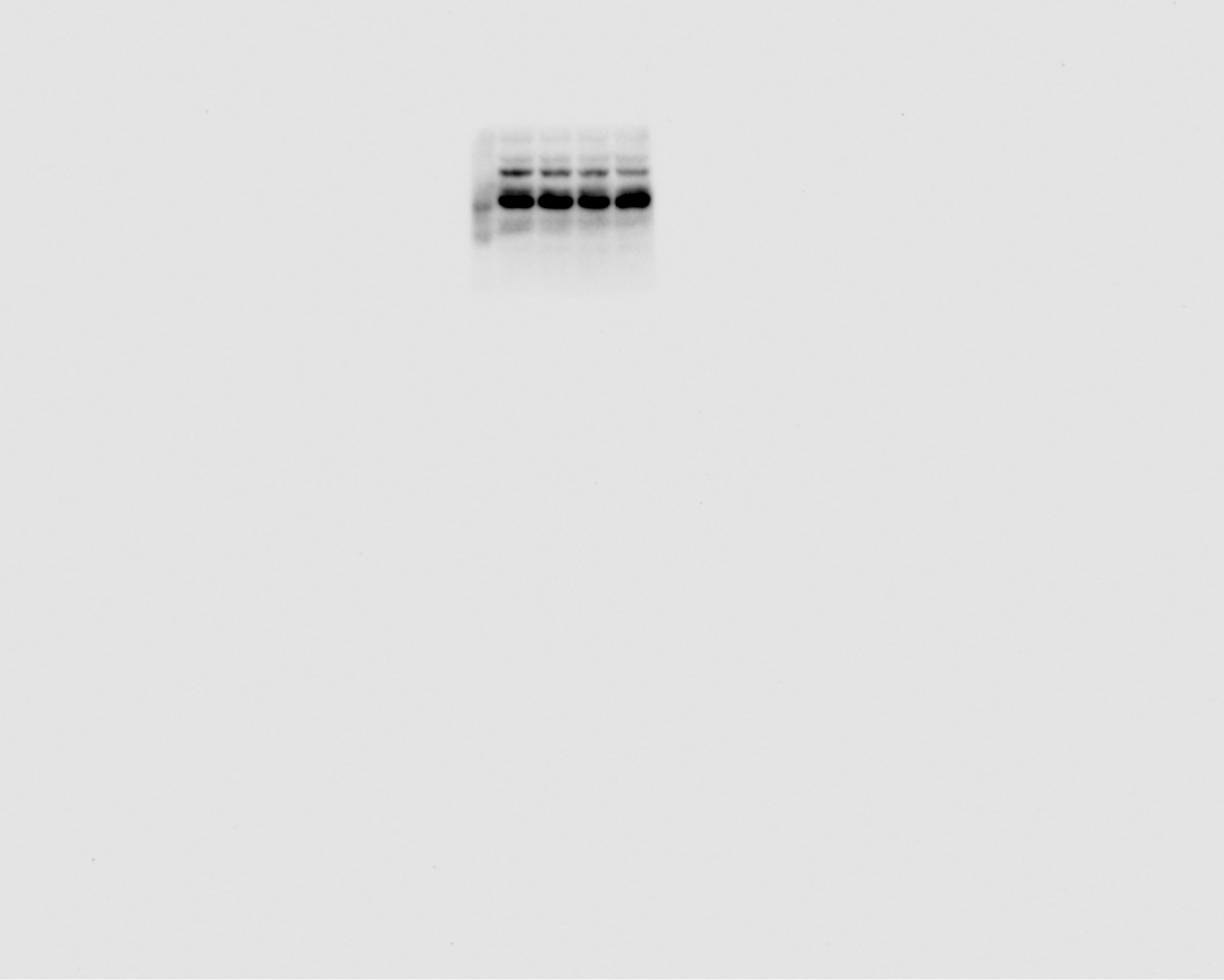

Supplement: Figure 10—source data 2. [file elife-89740-fig10-data2.zip › Figure 10-data2/Figure 10—data 2-(G).jpg]

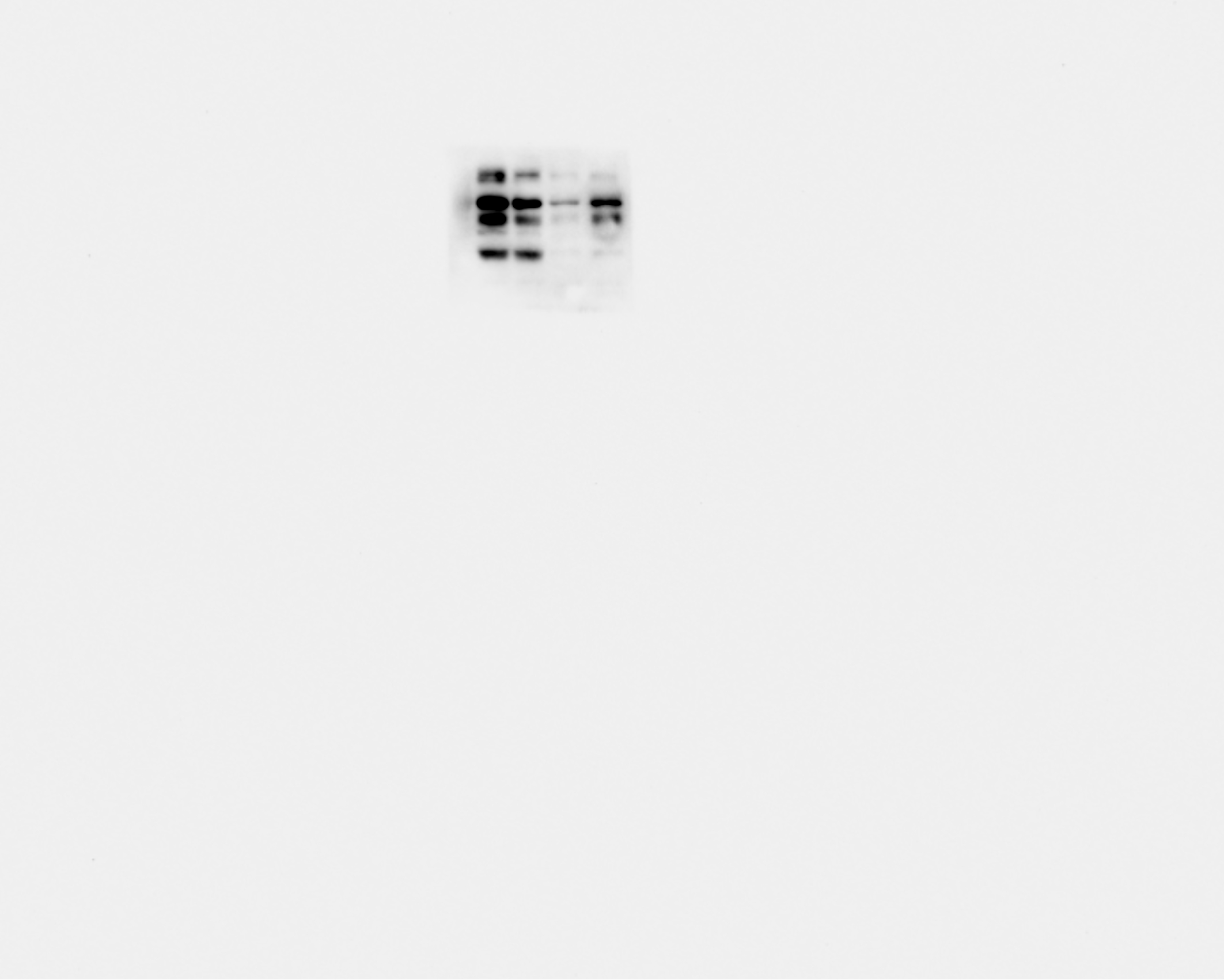

Supplement: Figure 10—source data 2. [file elife-89740-fig10-data2.zip › Figure 10-data2/Figure 10—data 2-(H).jpg]

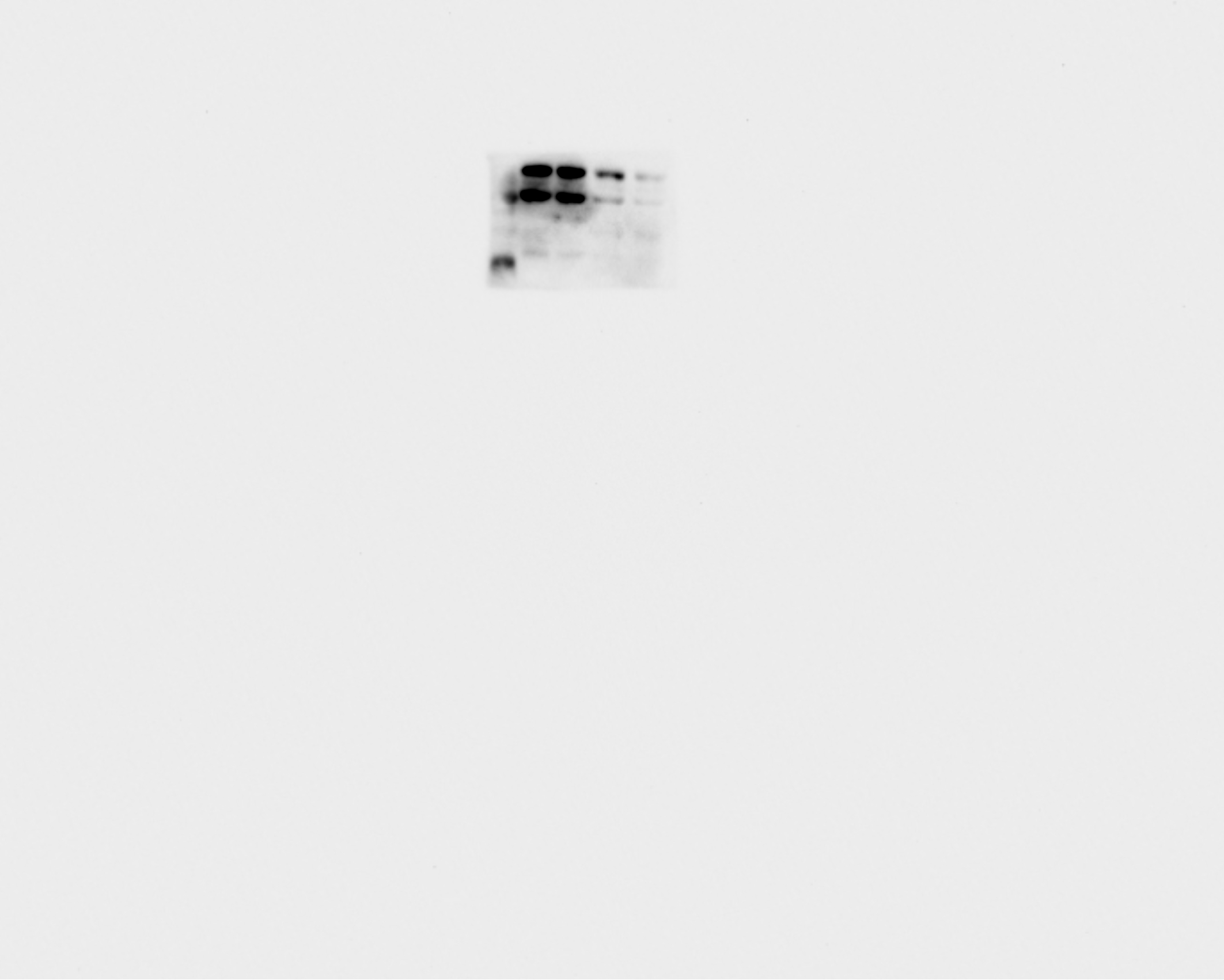

Supplement: Figure 10—source data 2. [file elife-89740-fig10-data2.zip › Figure 10-data2/Figure 10—data 2-(I).jpg]

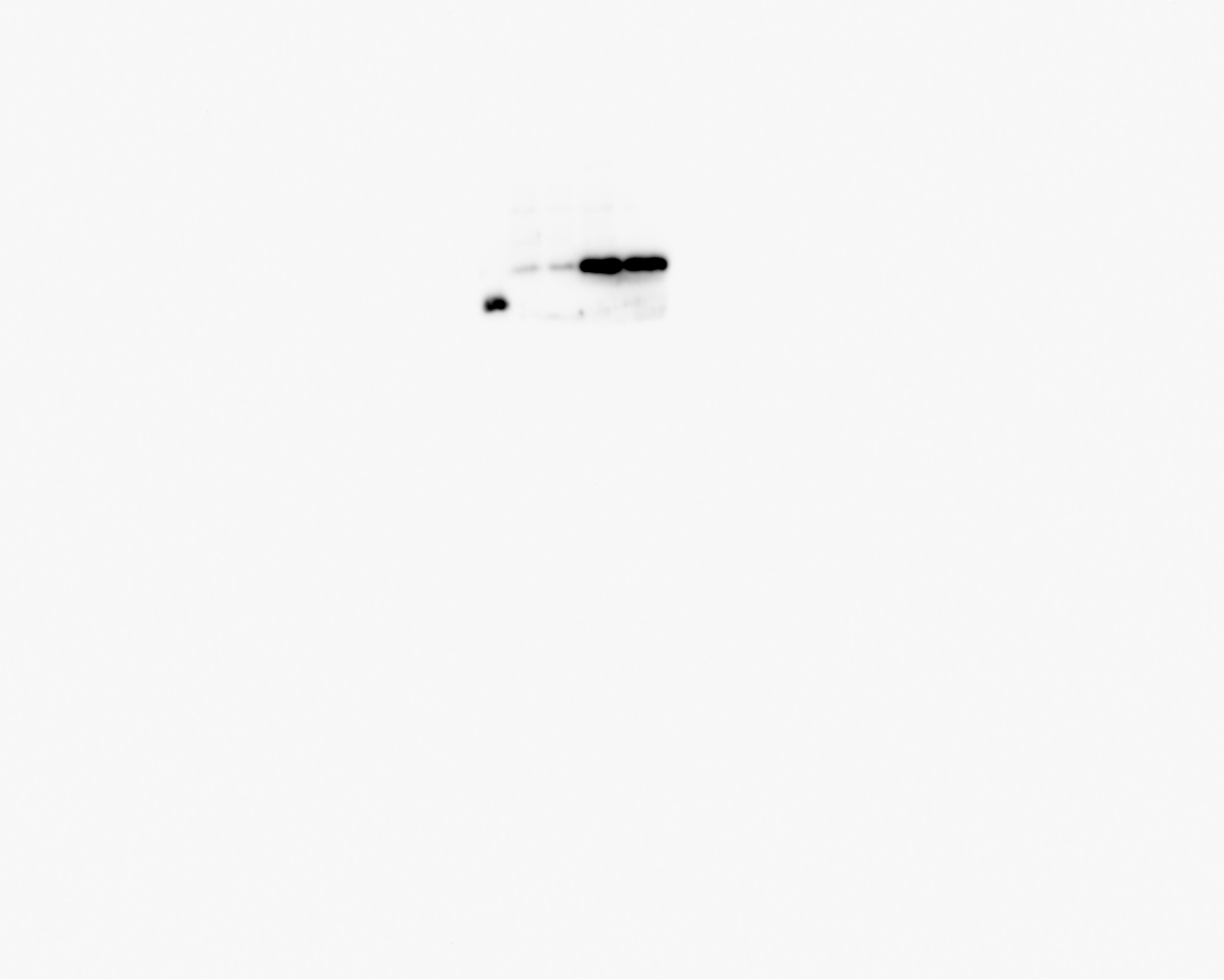

Supplement: Figure 10—source data 2. [file elife-89740-fig10-data2.zip › Figure 10-data2/Figure 10—data 2-(J).jpg]

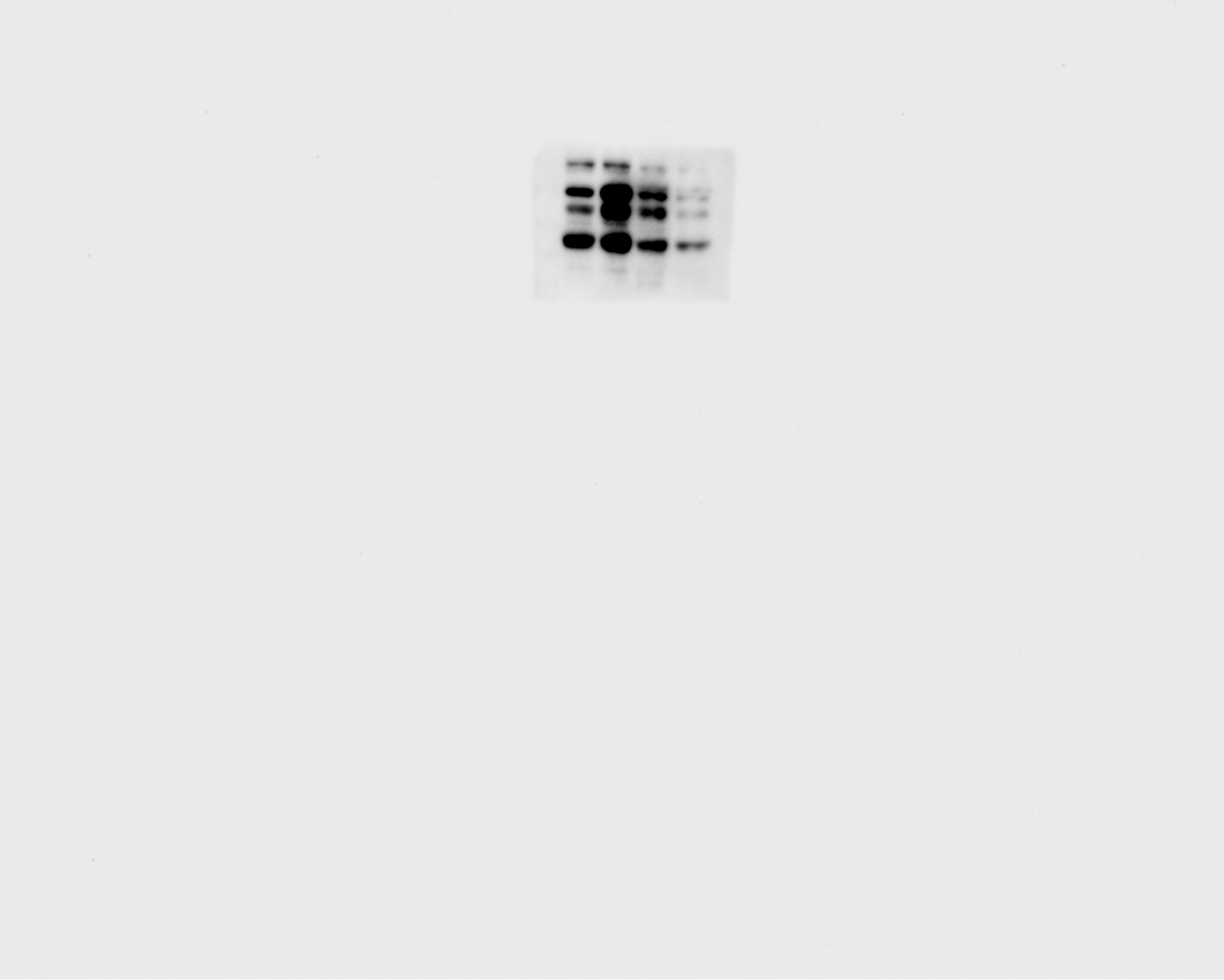

Supplement: Figure 10—source data 2. [file elife-89740-fig10-data2.zip › Figure 10-data2/Figure 10—data 2-(K).jpg]

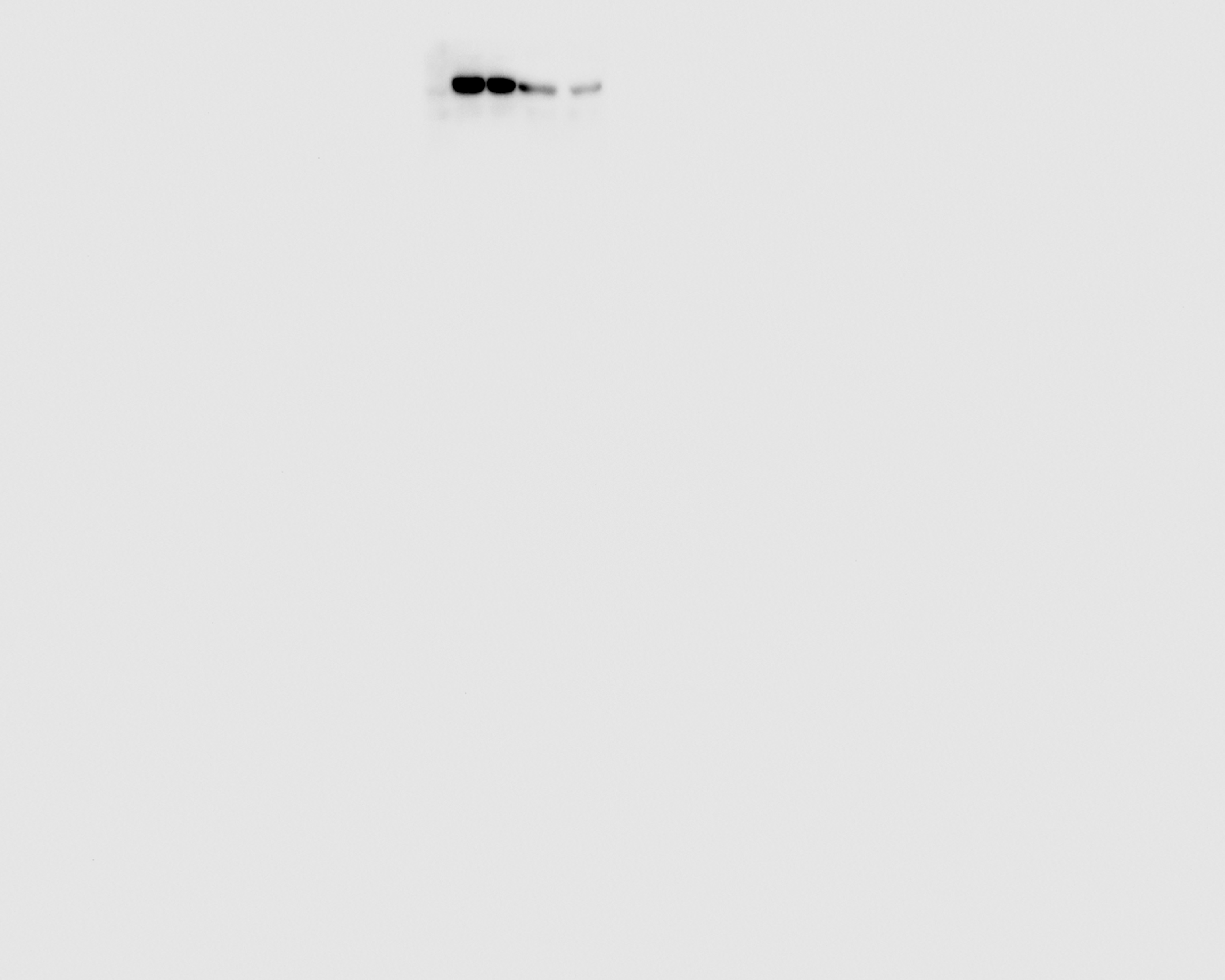

Supplement: Figure 10—source data 2. [file elife-89740-fig10-data2.zip › Figure 10-data2/Figure 10—data 2-(L).tif]

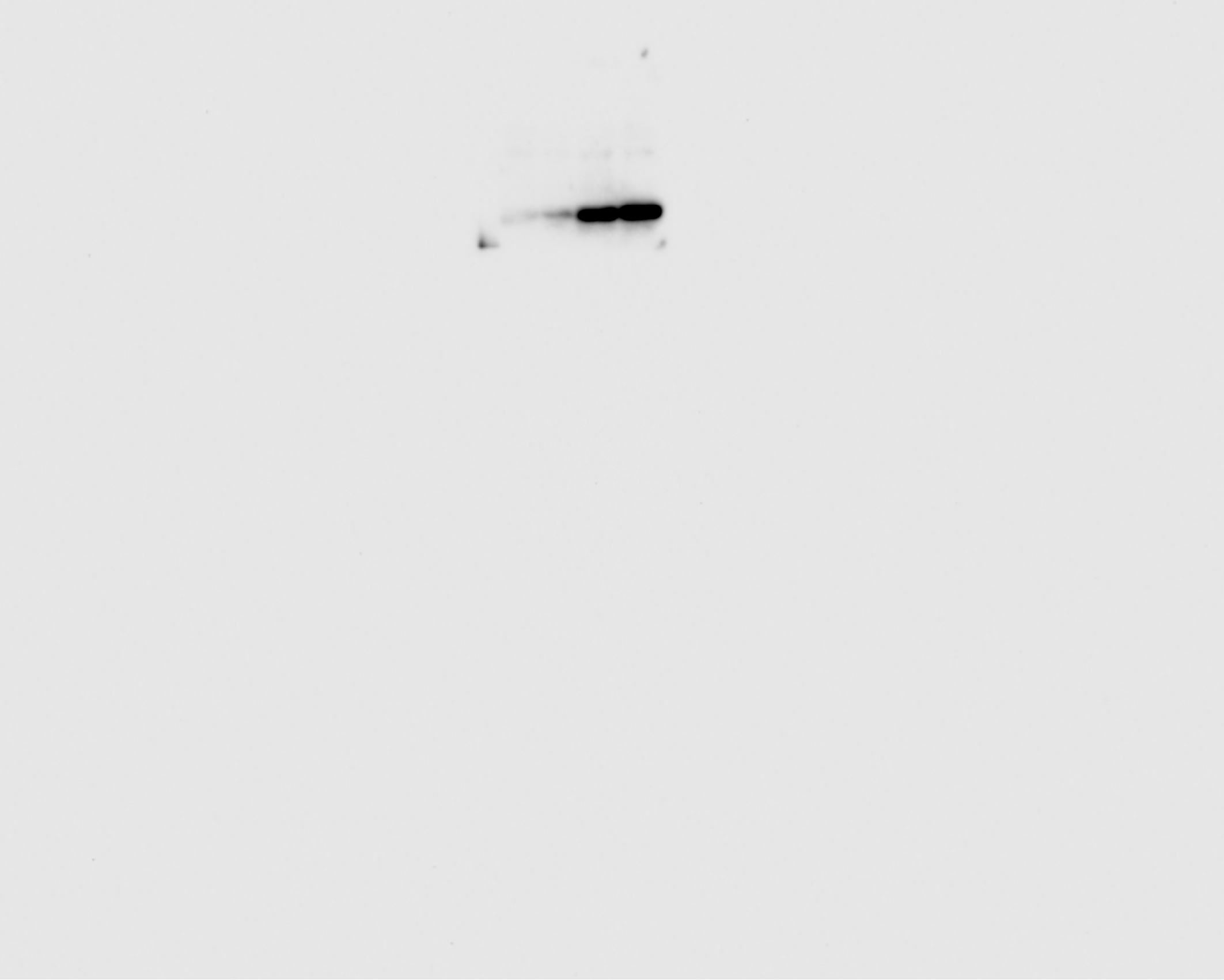

Supplement: Figure 10—source data 2. [file elife-89740-fig10-data2.zip › Figure 10-data2/Figure 10—data 2-(M).jpg]

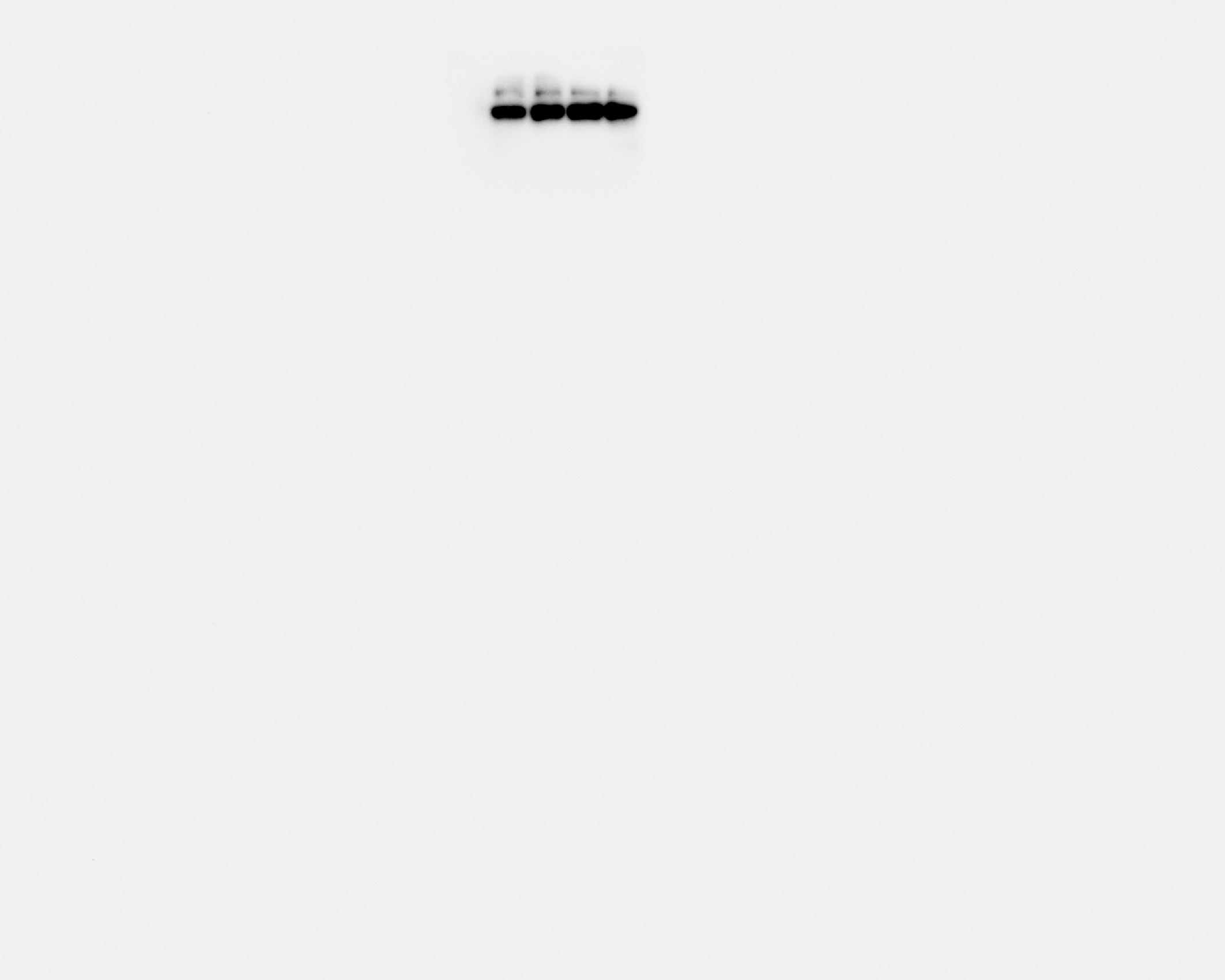

Supplement: Figure 10—source data 2. [file elife-89740-fig10-data2.zip › Figure 10-data2/Figure 10—data 2-(N).tif]

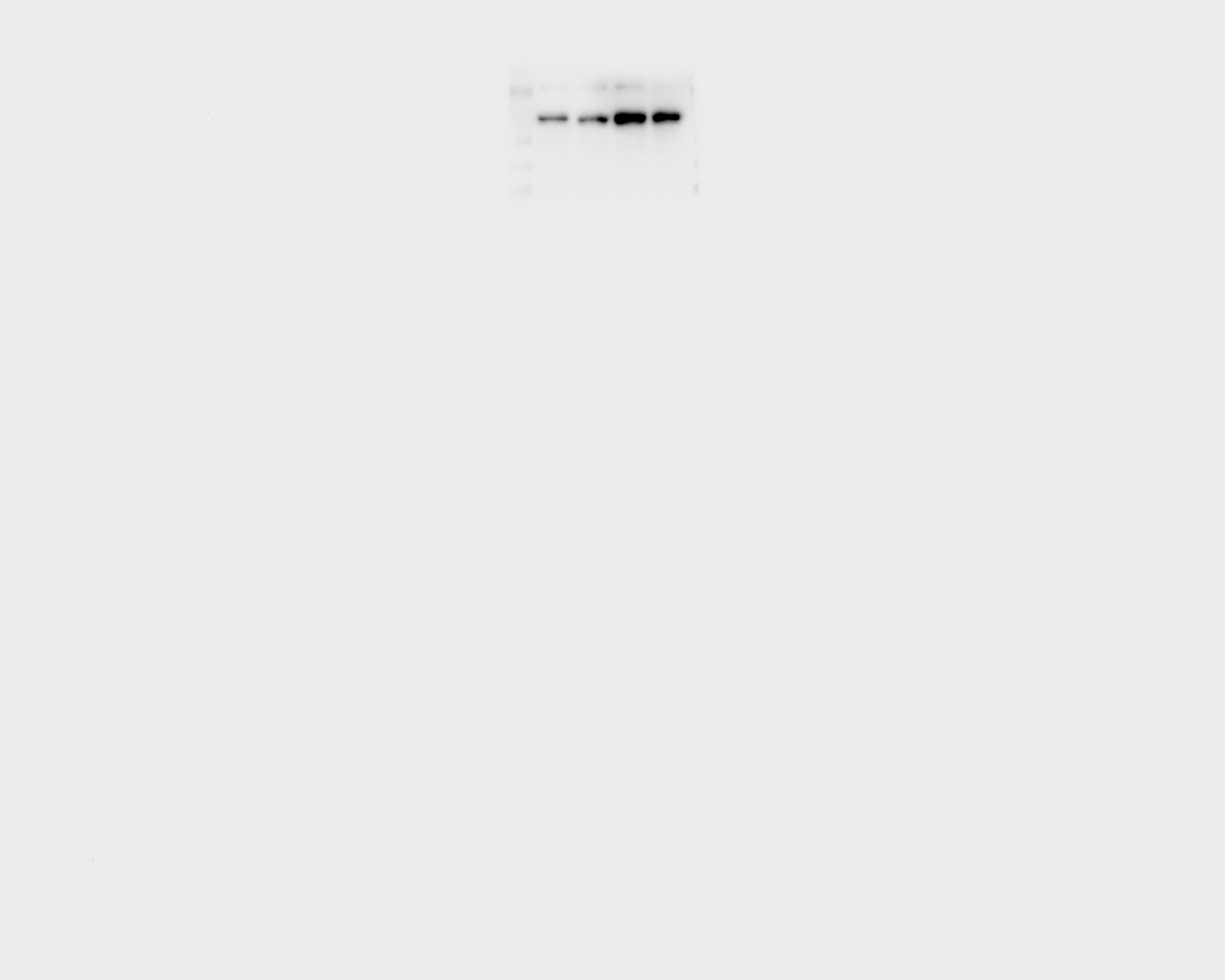

Supplement: Figure 10—source data 2. [file elife-89740-fig10-data2.zip › Figure 10-data2/Figure 10—data 2-(O).tif]

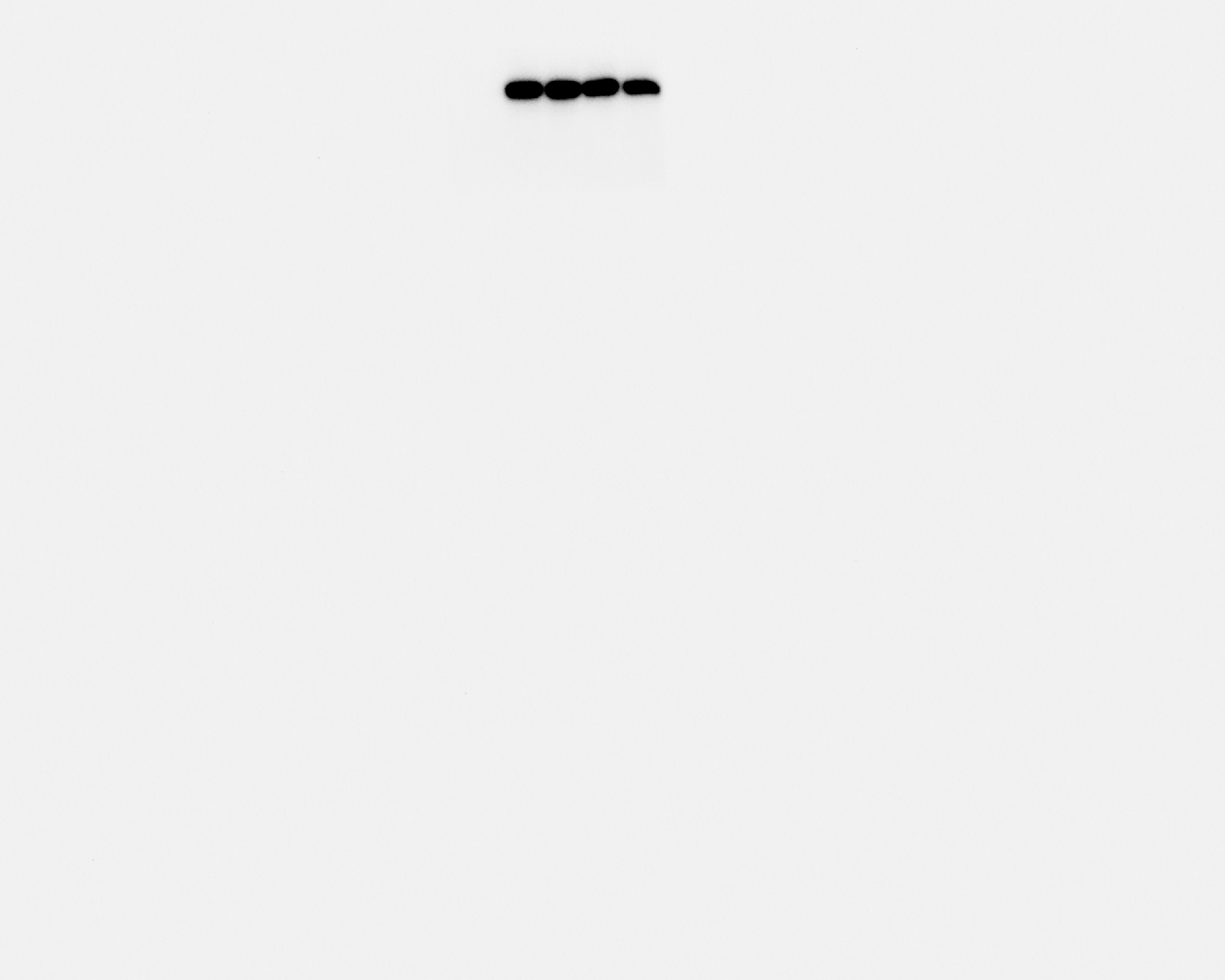

Supplement: Figure 10—source data 2. [file elife-89740-fig10-data2.zip › Figure 10-data2/Figure 10—data 2-(P).tif]

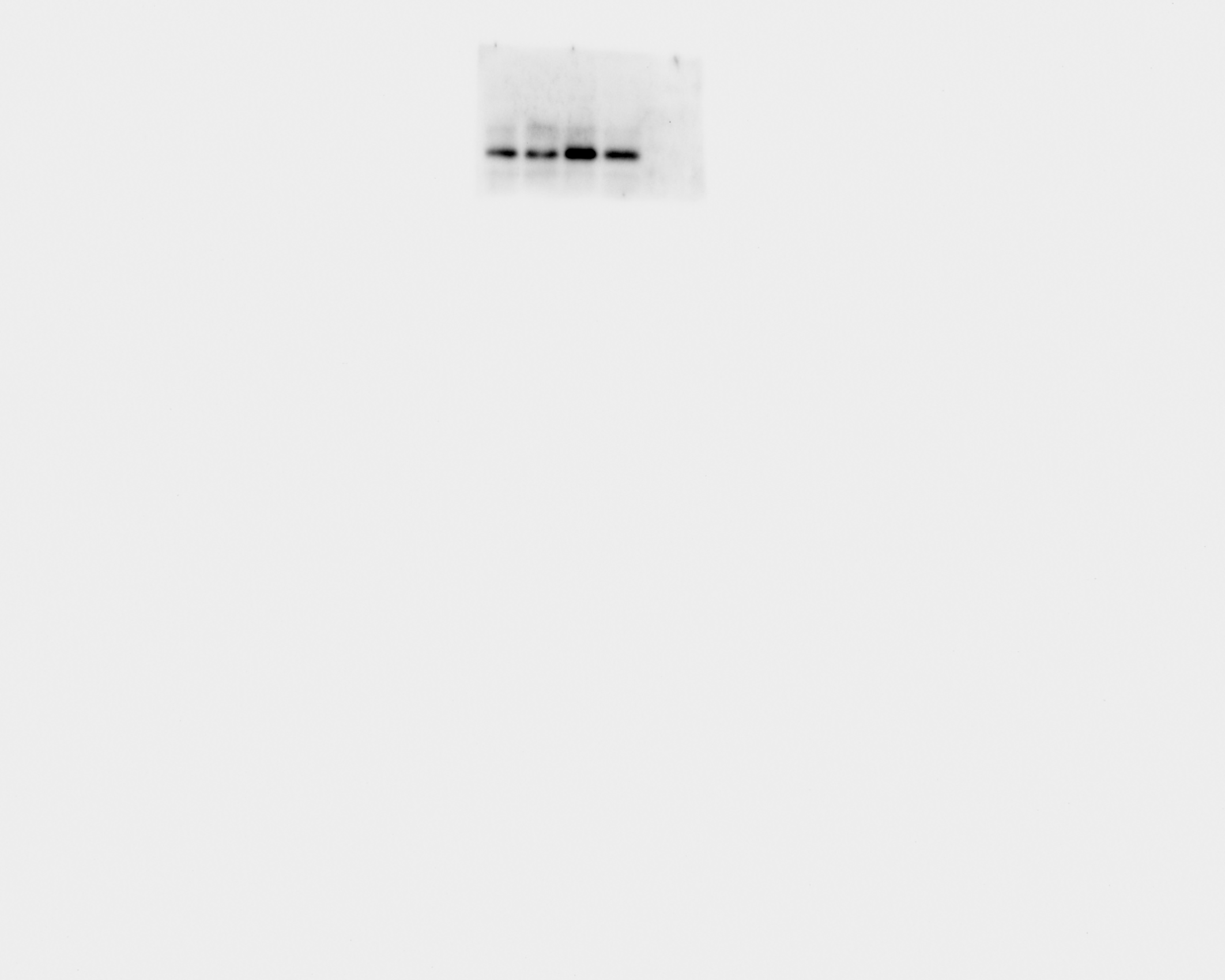

Supplement: Figure 10—source data 2. [file elife-89740-fig10-data2.zip › Figure 10-data2/Figure 10—data 2-(Q).tif]

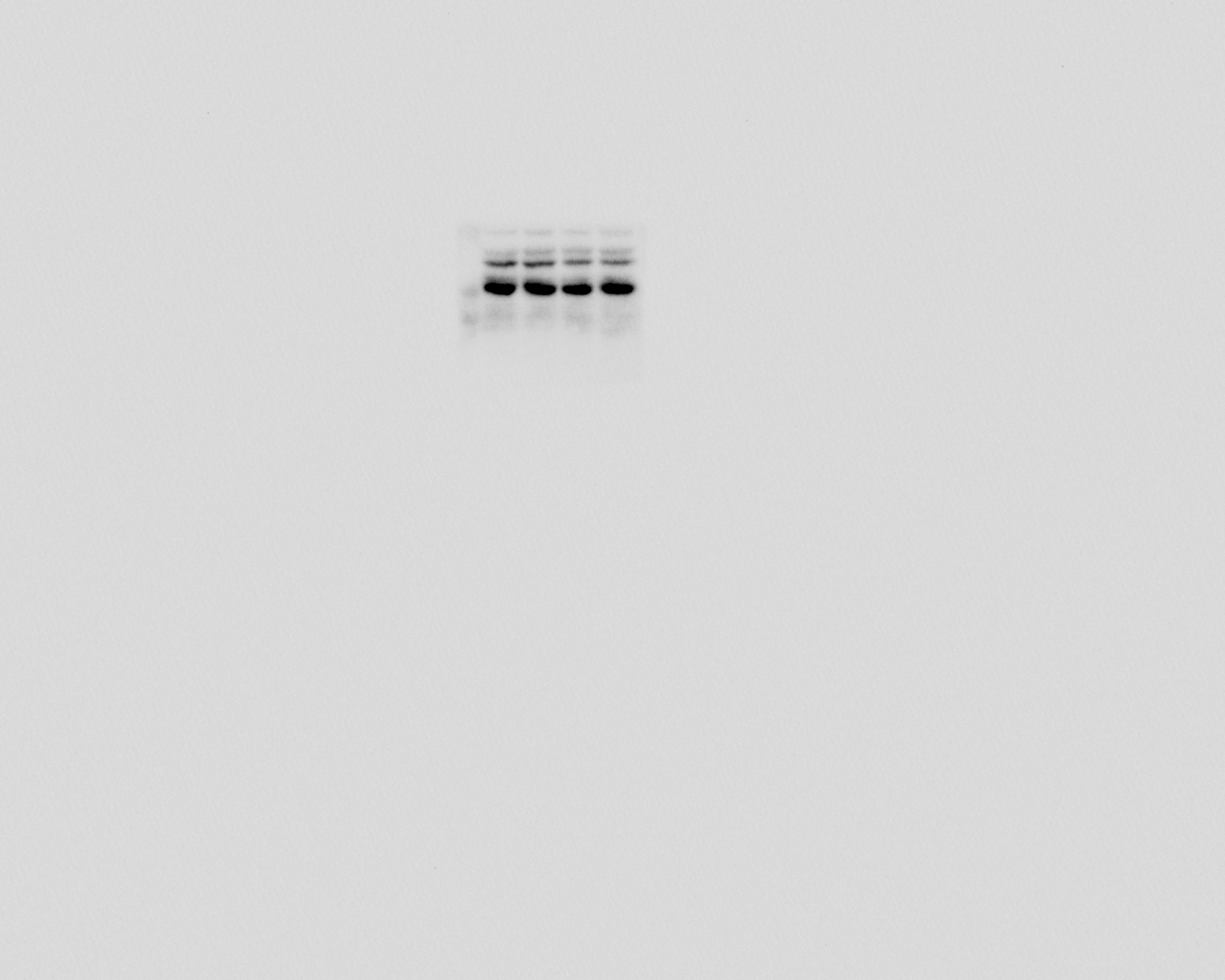

Supplement: Figure 10—source data 2. [file elife-89740-fig10-data2.zip › Figure 10-data2/Figure 10—data 2-(R).tif]
