# Supplementary material for: PSTPIP2 ameliorates aristolochic acid nephropathy by suppressing interleukin-19-mediated neutrophil extracellular trap formation
Source: eLife. 2024 Feb 5;13:e89740. doi: 10.7554/eLife.89740 (PMC10906995; doi:10.7554/eLife.89740)
Supplement: Figure 1—figure supplement 1—source data 2. [file elife-89740-fig1-figsupp1-data2.zip › Figure 1-figure supplement 1-data 2/Figure 1-figure supplement 1—source data 2.pptx]

## Slide 1
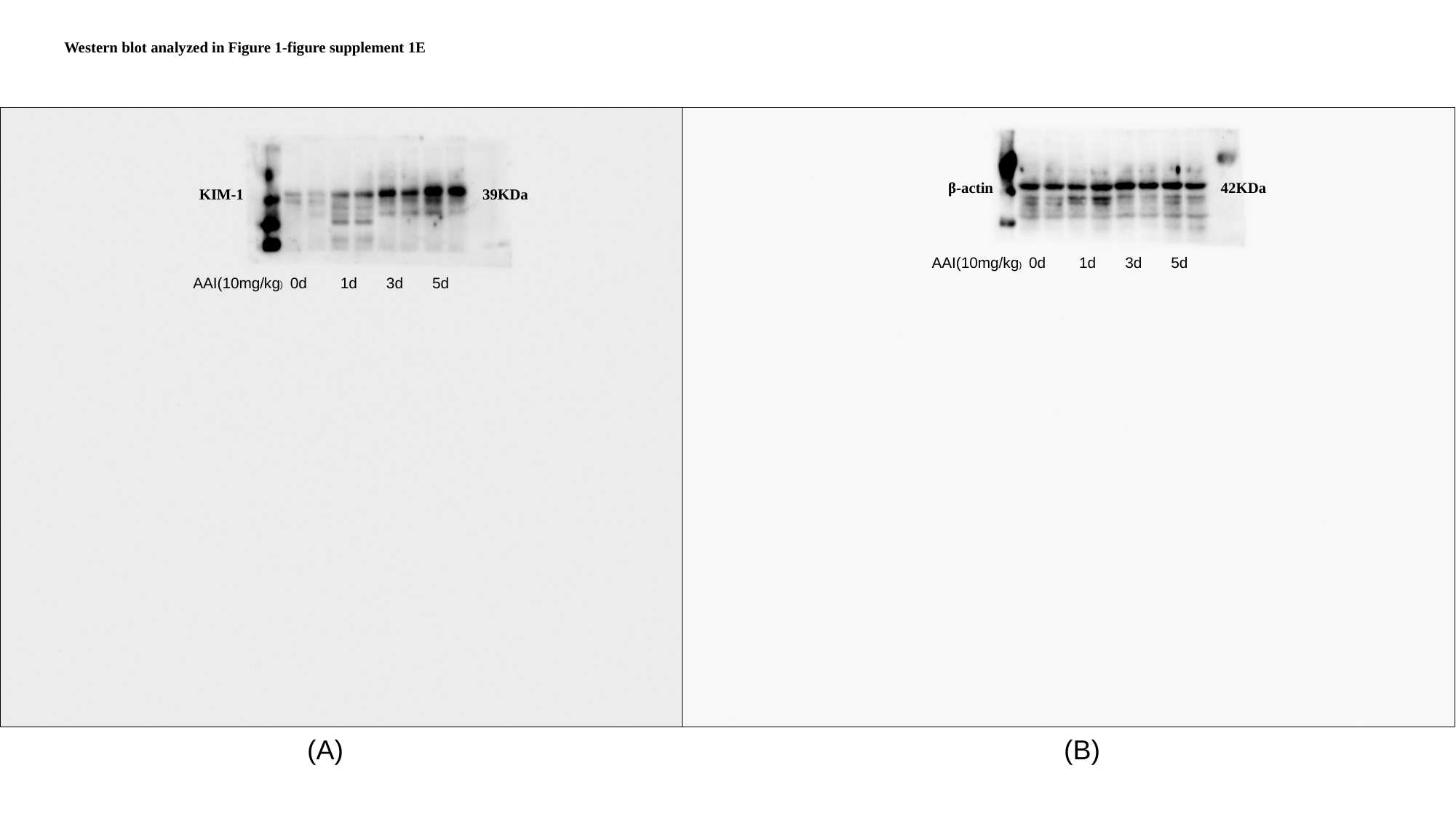

Western blot analyzed in Figure 1-figure supplement 1E
42KDa
β-actin
KIM-1
39KDa
AAI(10mg/kg) 0d 1d 3d 5d
AAI(10mg/kg) 0d 1d 3d 5d
(A)
(B)

## Slide 2
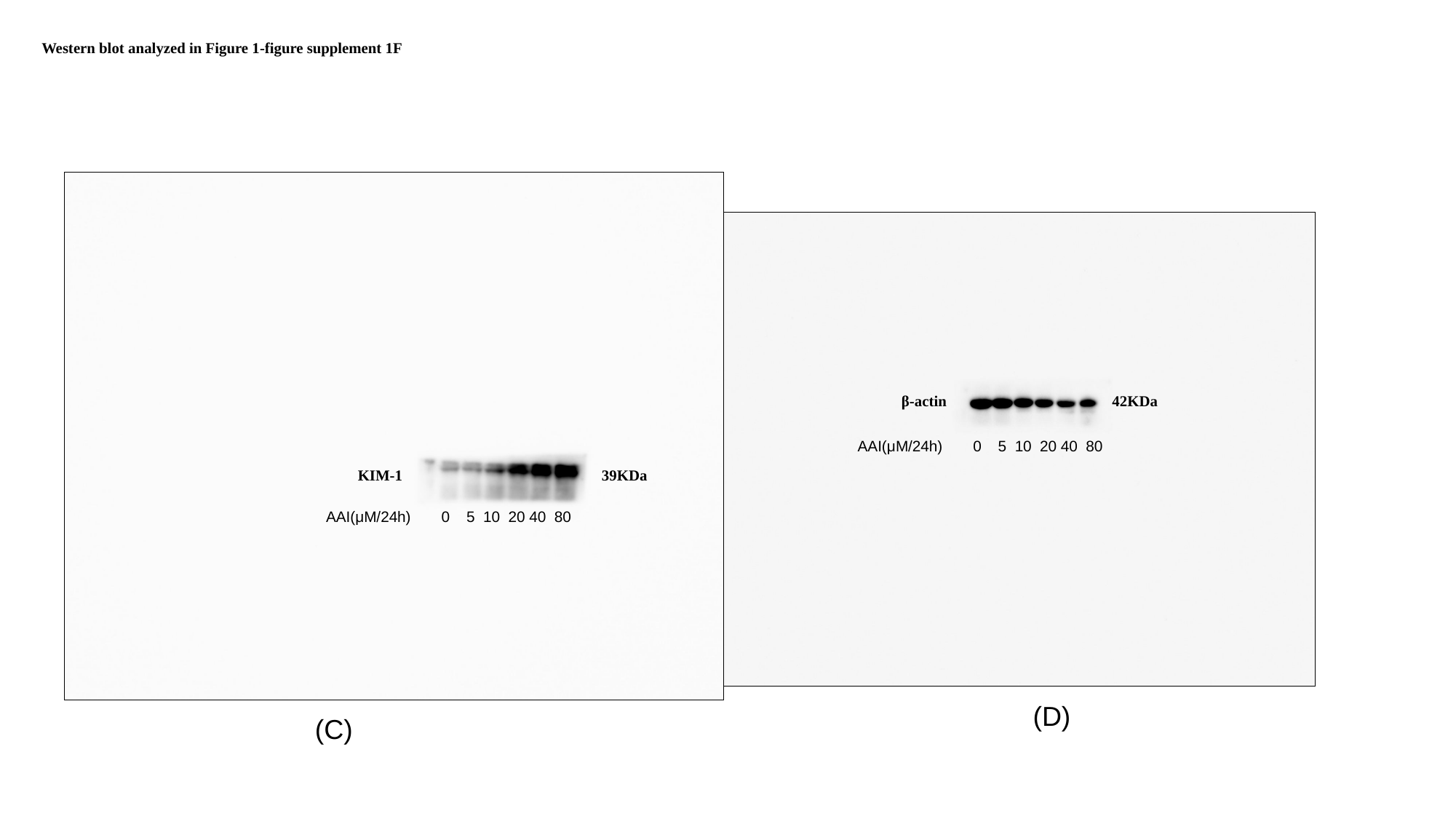

Western blot analyzed in Figure 1-figure supplement 1F
42KDa
β-actin
AAI(μM/24h)
 0 5 10 20 40 80
KIM-1
39KDa
AAI(μM/24h)
 0 5 10 20 40 80
(D)
(C)

## Slide 3
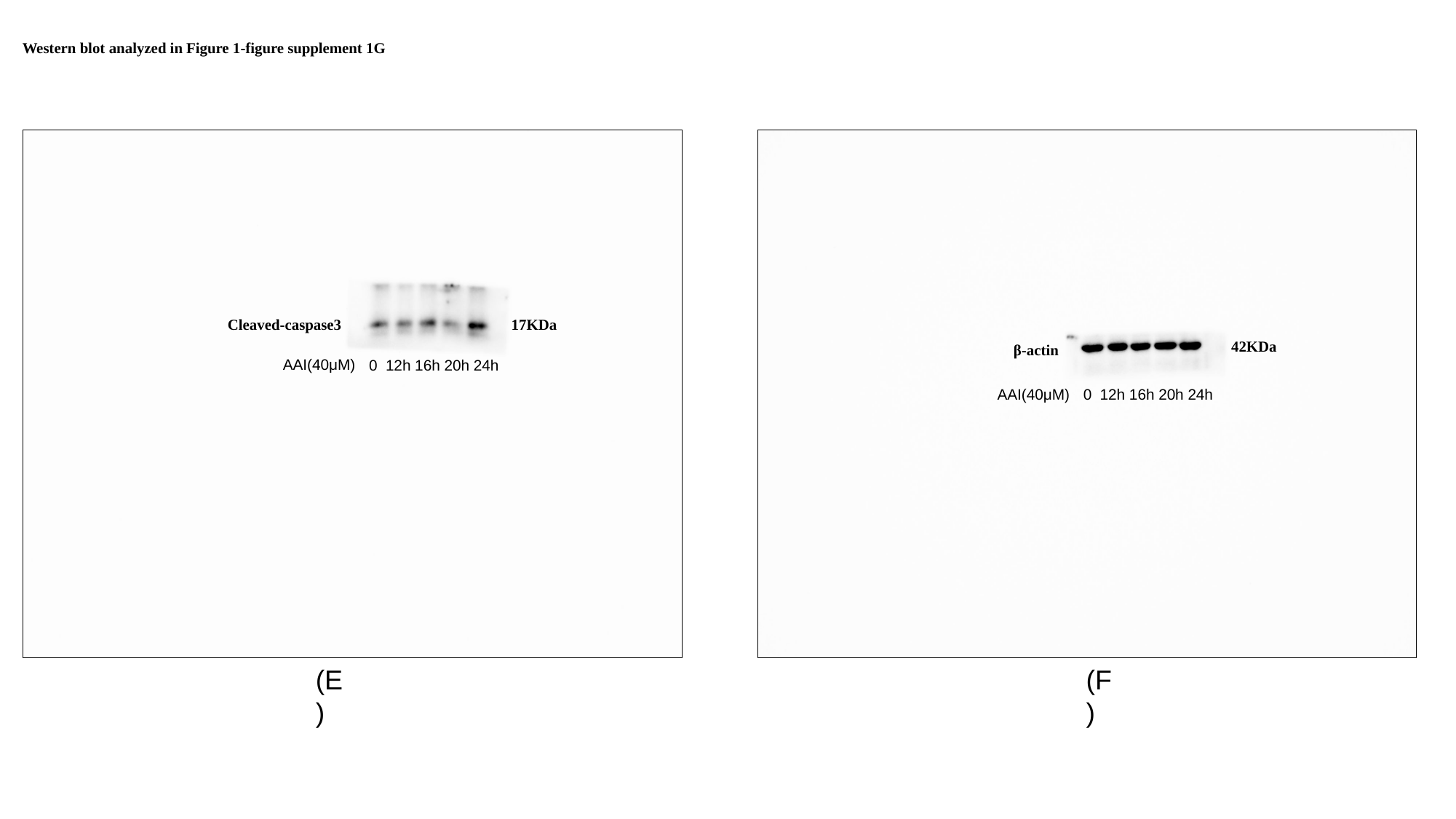

Western blot analyzed in Figure 1-figure supplement 1G
Cleaved-caspase3
17KDa
42KDa
β-actin
 AAI(40μM)
 0 12h 16h 20h 24h
 AAI(40μM)
 0 12h 16h 20h 24h
(E)
(F)
