# Supplementary material for: PSTPIP2 ameliorates aristolochic acid nephropathy by suppressing interleukin-19-mediated neutrophil extracellular trap formation
Source: eLife. 2024 Feb 5;13:e89740. doi: 10.7554/eLife.89740 (PMC10906995; doi:10.7554/eLife.89740)
Supplement: Figure 2—source data 2. [file elife-89740-fig2-data2.zip › Figure 2-data 2/Figure 2—source data 2.pptx]

## Slide 1
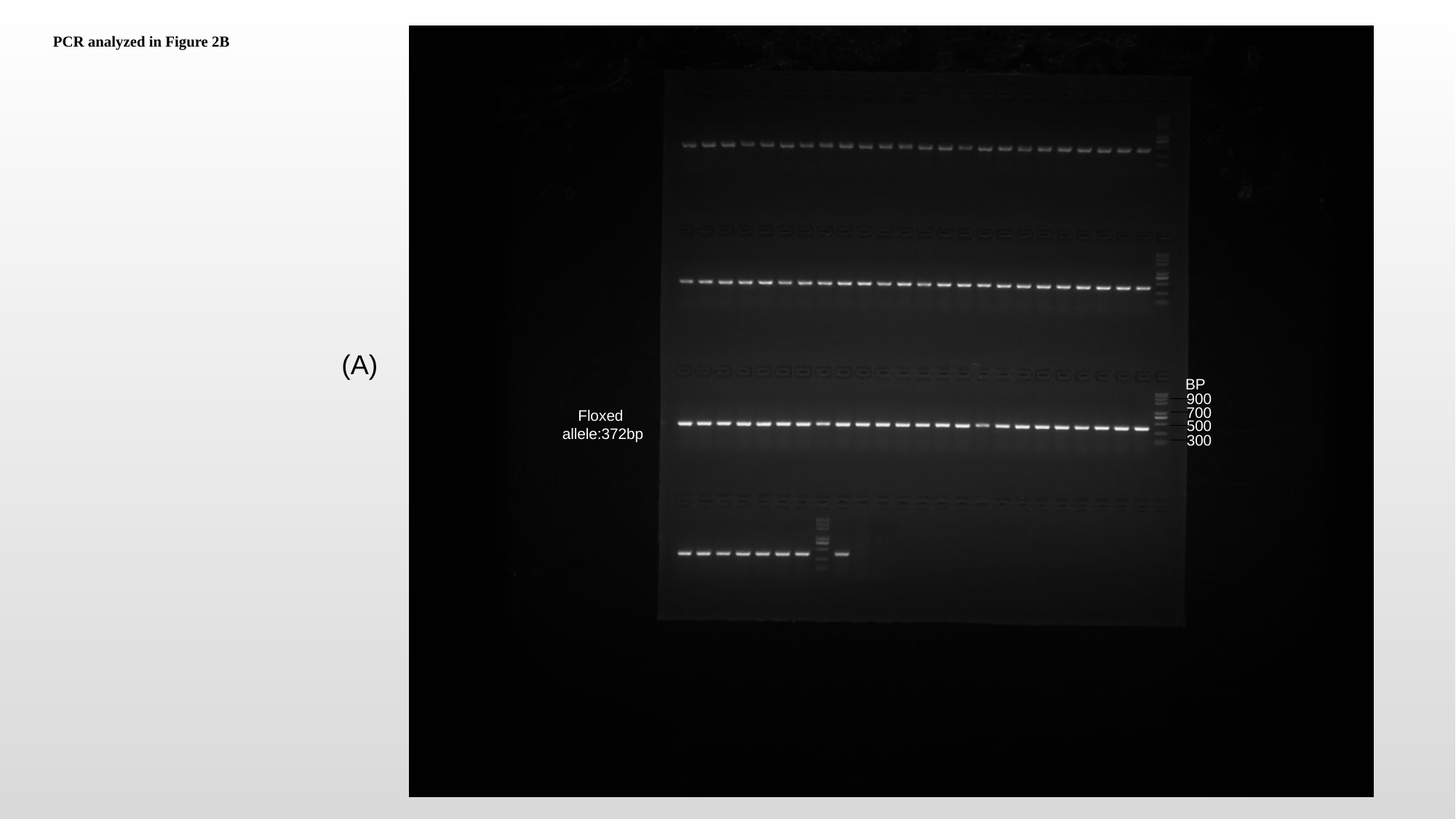

PCR analyzed in Figure 2B
(A)
BP
900
700
500
300
Floxed
allele:372bp

## Slide 2
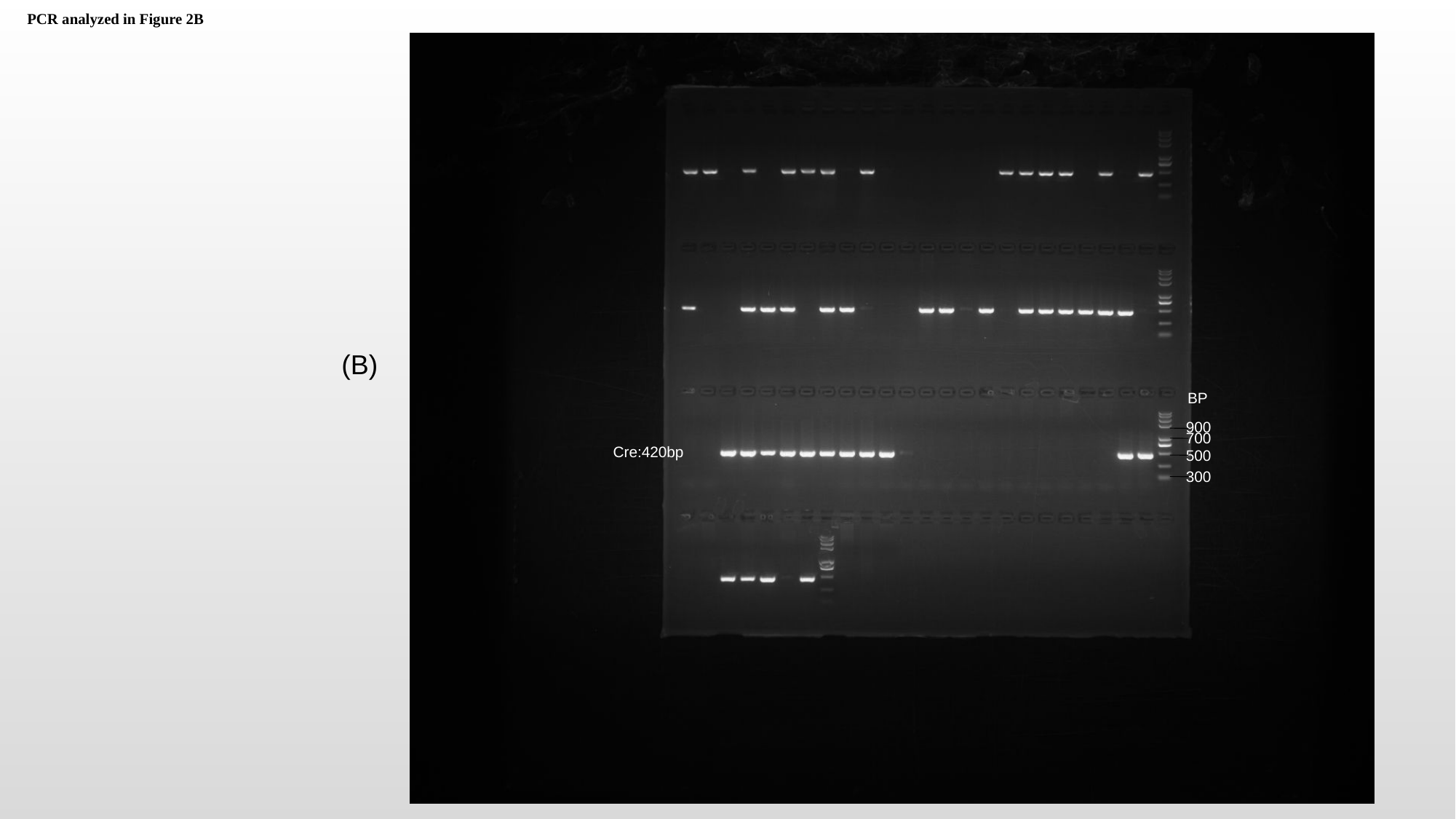

PCR analyzed in Figure 2B
(B)
BP
900
700
500
300
Cre:420bp

## Slide 3
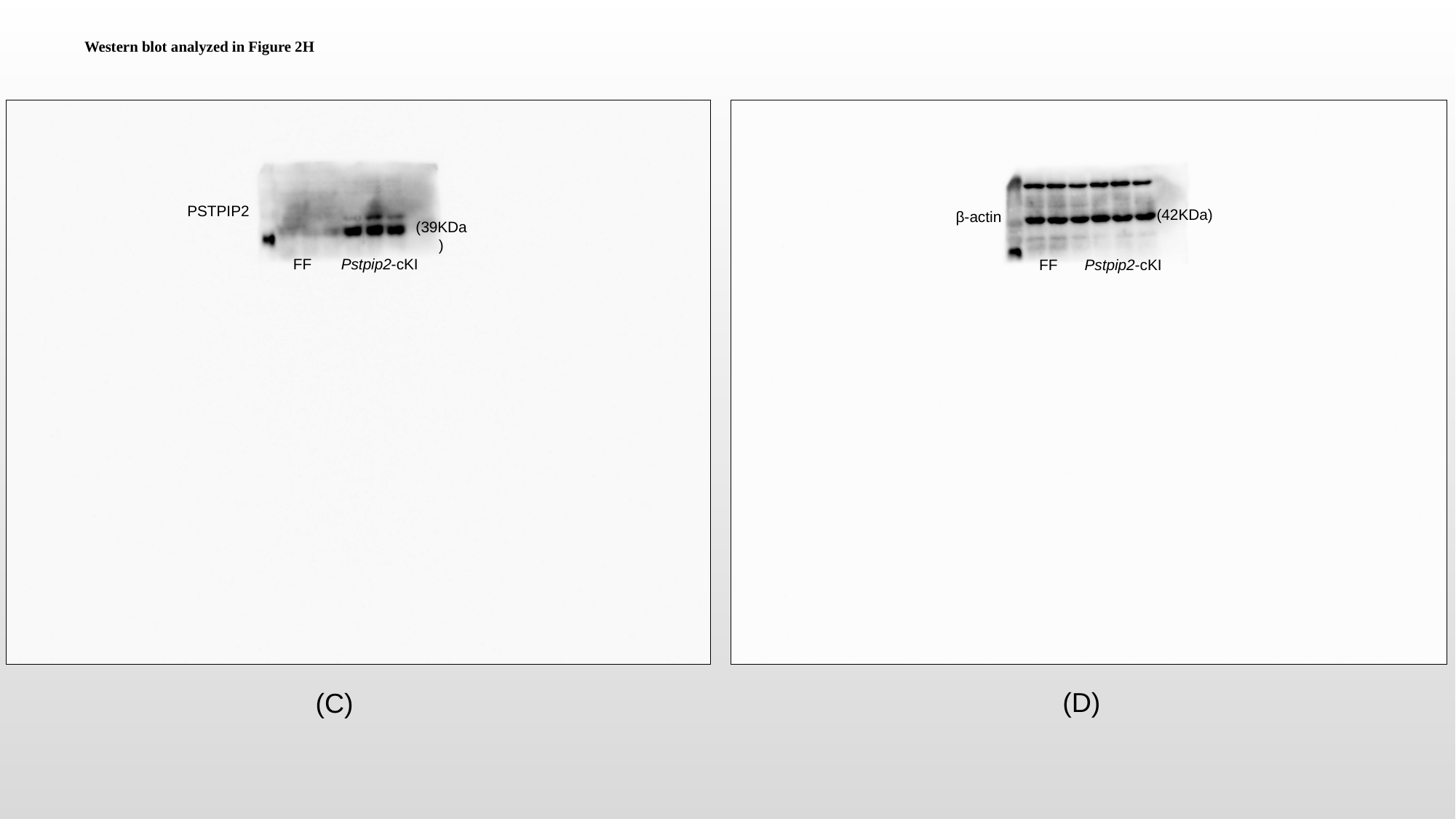

Western blot analyzed in Figure 2H
PSTPIP2
(42KDa)
β-actin
(39KDa)
FF
Pstpip2-cKI
FF
Pstpip2-cKI
(D)
(C)

## Slide 4
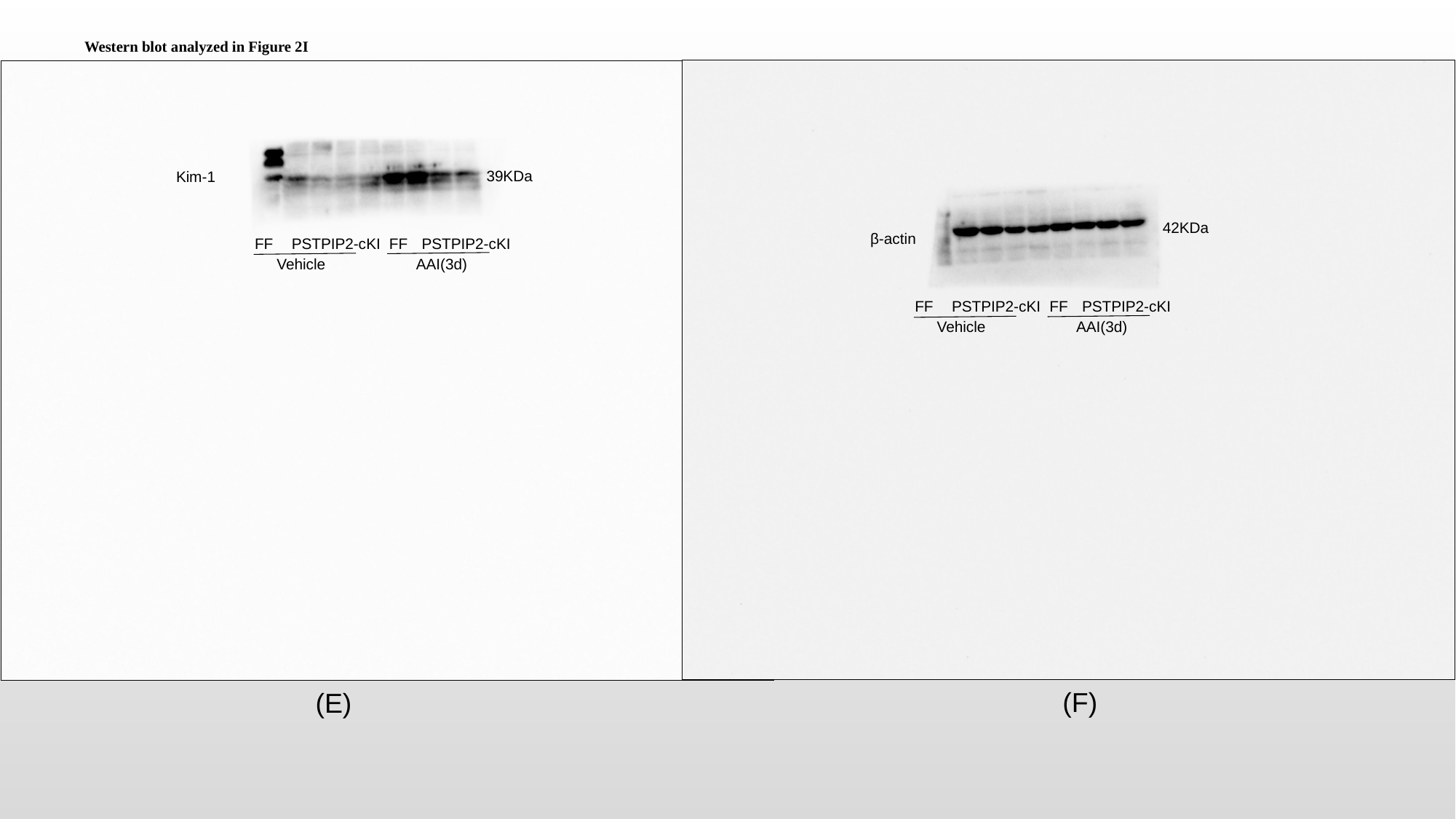

Western blot analyzed in Figure 2I
39KDa
Kim-1
42KDa
β-actin
FF
PSTPIP2-cKI
FF
PSTPIP2-cKI
Vehicle
AAI(3d)
FF
PSTPIP2-cKI
FF
PSTPIP2-cKI
Vehicle
AAI(3d)
(F)
(E)
