# Supplementary material for: PSTPIP2 ameliorates aristolochic acid nephropathy by suppressing interleukin-19-mediated neutrophil extracellular trap formation
Source: eLife. 2024 Feb 5;13:e89740. doi: 10.7554/eLife.89740 (PMC10906995; doi:10.7554/eLife.89740)
Supplement: Figure 2—figure supplement 1—source data 2. [file elife-89740-fig2-figsupp1-data2.zip › Figure 2-figure supplement 1-data 2/Figure 2-figure supplement 1—source data 2.pptx]

## Slide 1
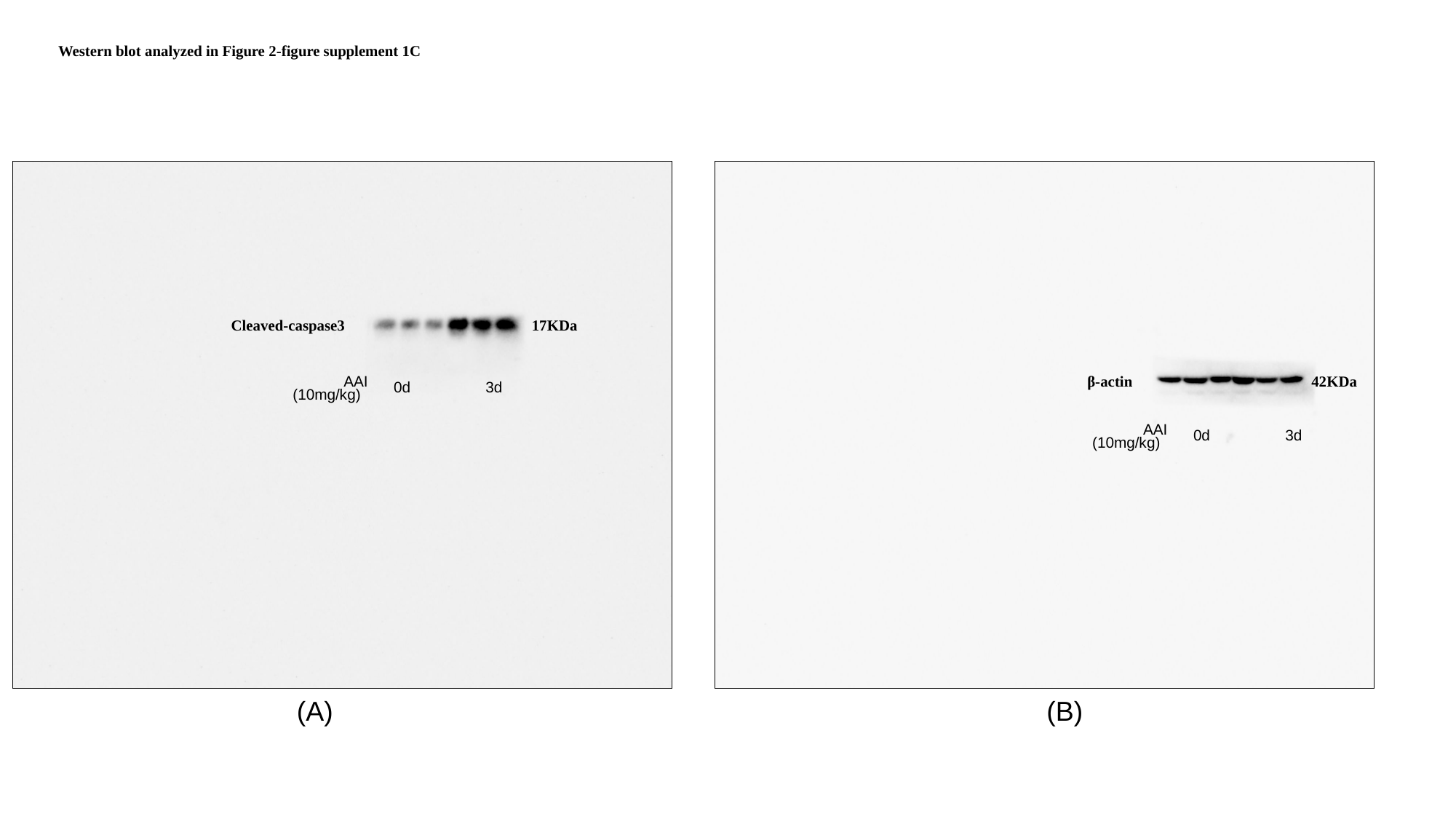

Western blot analyzed in Figure 2-figure supplement 1C
Cleaved-caspase3
17KDa
β-actin
42KDa
0d 3d
AAI
(10mg/kg)
0d 3d
AAI
(10mg/kg)
(A)
(B)

## Slide 2
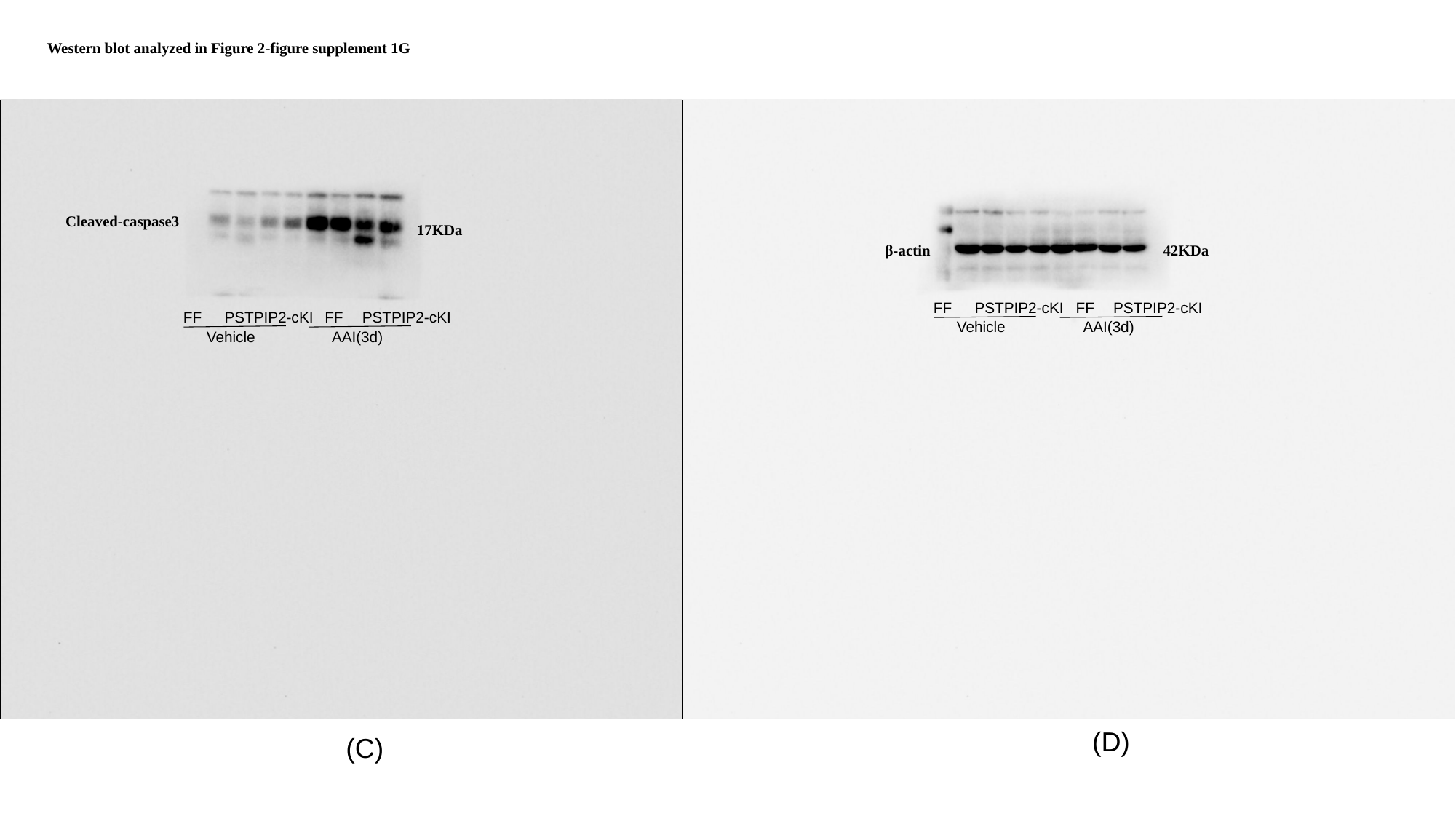

Western blot analyzed in Figure 2-figure supplement 1G
Cleaved-caspase3
17KDa
β-actin
42KDa
FF
PSTPIP2-cKI
Vehicle
FF
PSTPIP2-cKI
AAI(3d)
FF
PSTPIP2-cKI
Vehicle
FF
PSTPIP2-cKI
AAI(3d)
(D)
(C)
