# Supplementary material for: PSTPIP2 ameliorates aristolochic acid nephropathy by suppressing interleukin-19-mediated neutrophil extracellular trap formation
Source: eLife. 2024 Feb 5;13:e89740. doi: 10.7554/eLife.89740 (PMC10906995; doi:10.7554/eLife.89740)
Supplement: Figure 3—source data 2. [file elife-89740-fig3-data2.zip › Figure 3-data 2/Figure 3—source data 2.pptx]

## Slide 1
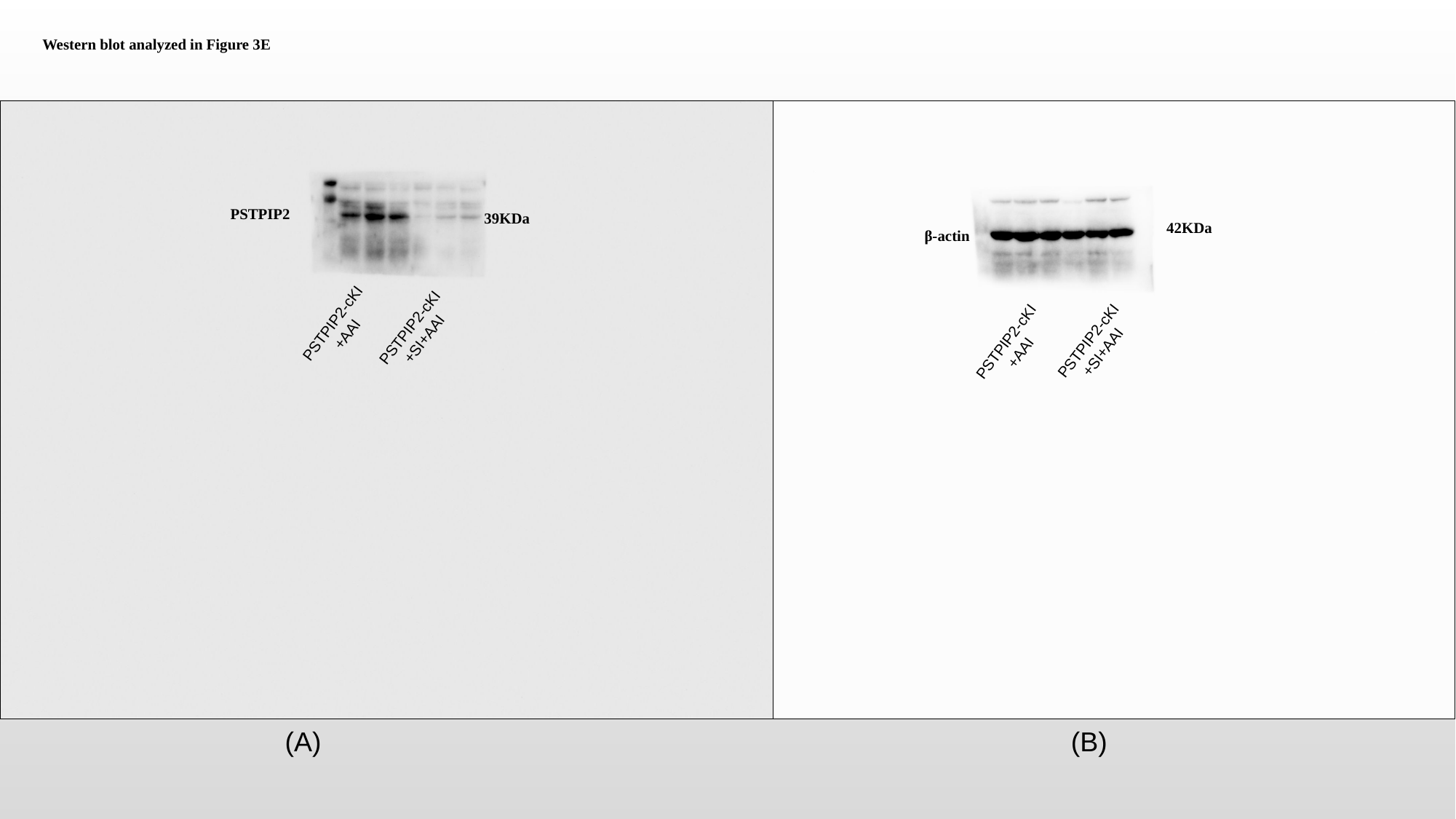

Western blot analyzed in Figure 3E
PSTPIP2
39KDa
42KDa
β-actin
PSTPIP2-cKI
+AAI
PSTPIP2-cKI
+SI+AAI
PSTPIP2-cKI
+SI+AAI
PSTPIP2-cKI
+AAI
(A)
(B)

## Slide 2
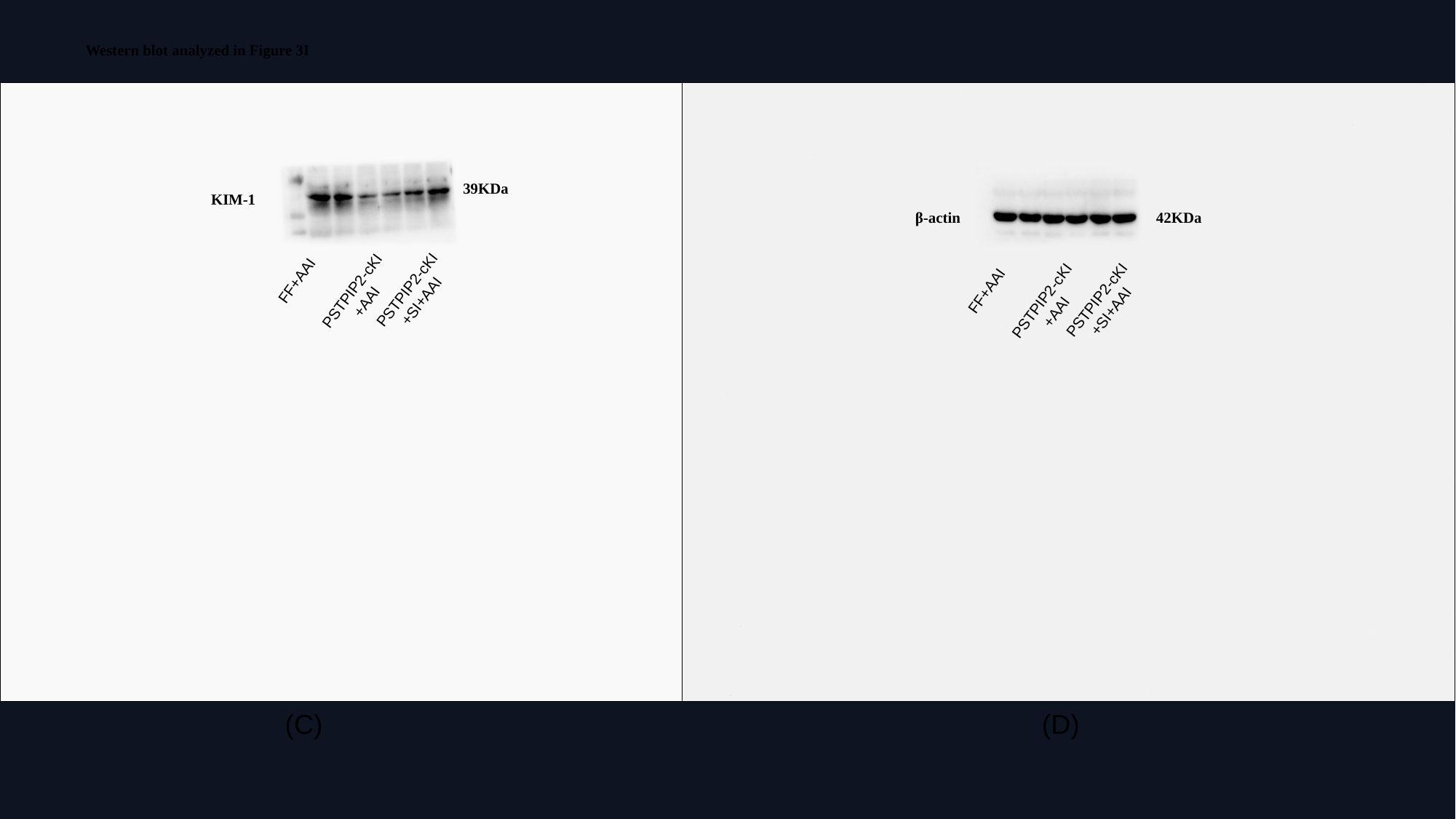

Western blot analyzed in Figure 3I
39KDa
KIM-1
β-actin
42KDa
FF+AAI
PSTPIP2-cKI
+SI+AAI
PSTPIP2-cKI
+AAI
FF+AAI
PSTPIP2-cKI
+SI+AAI
PSTPIP2-cKI
+AAI
(C)
(D)

## Slide 3
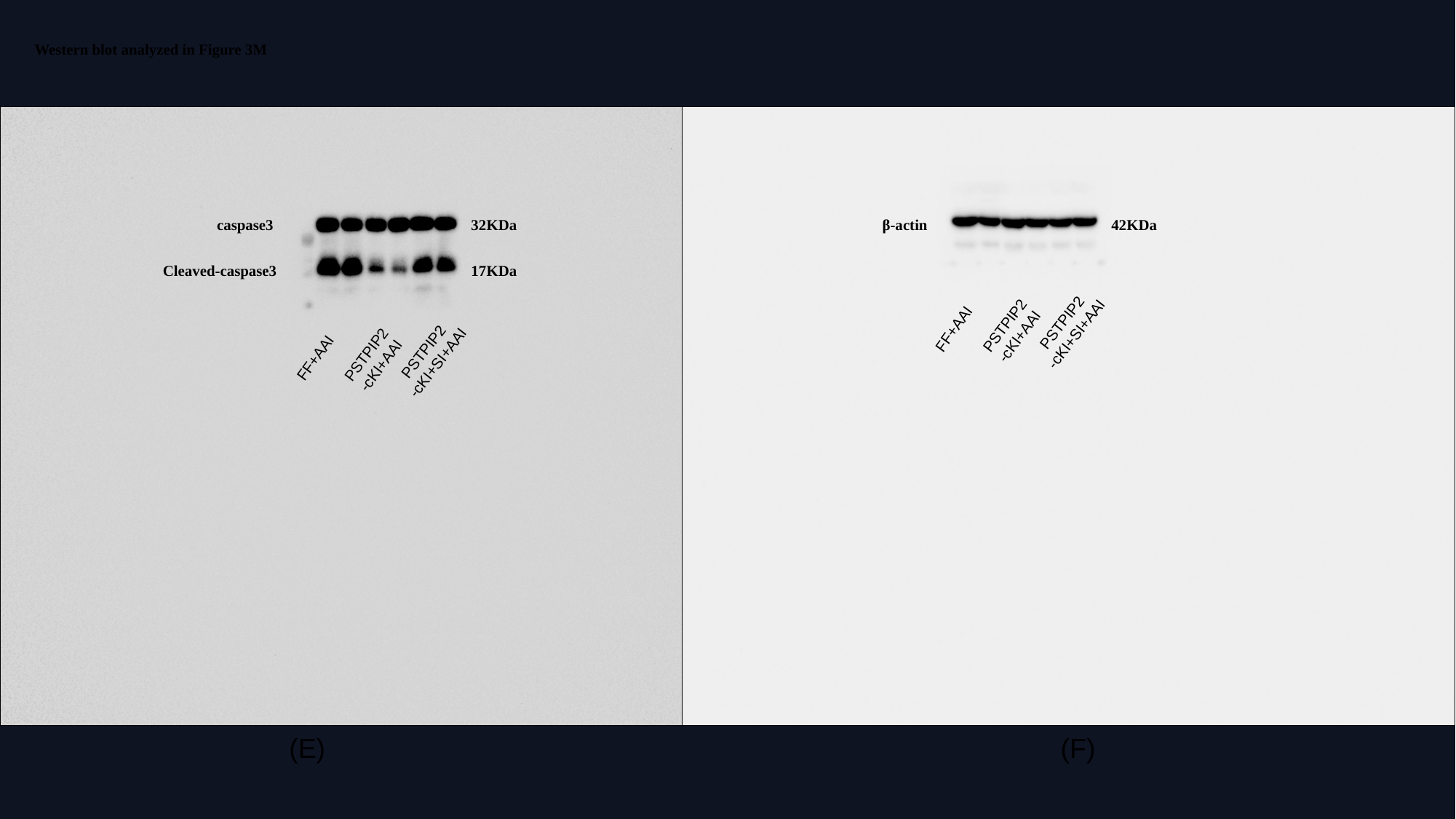

Western blot analyzed in Figure 3M
32KDa
caspase3
β-actin
42KDa
17KDa
Cleaved-caspase3
PSTPIP2
-cKI+SI+AAI
PSTPIP2
-cKI+AAI
FF+AAI
PSTPIP2
-cKI+SI+AAI
PSTPIP2
-cKI+AAI
FF+AAI
(E)
(F)
