# Supplementary material for: PSTPIP2 ameliorates aristolochic acid nephropathy by suppressing interleukin-19-mediated neutrophil extracellular trap formation
Source: eLife. 2024 Feb 5;13:e89740. doi: 10.7554/eLife.89740 (PMC10906995; doi:10.7554/eLife.89740)
Supplement: Figure 5—source data 2. [file elife-89740-fig5-data2.zip › Figure 5-data 2/Figure 5—source data 2.pptx]

## Slide 1
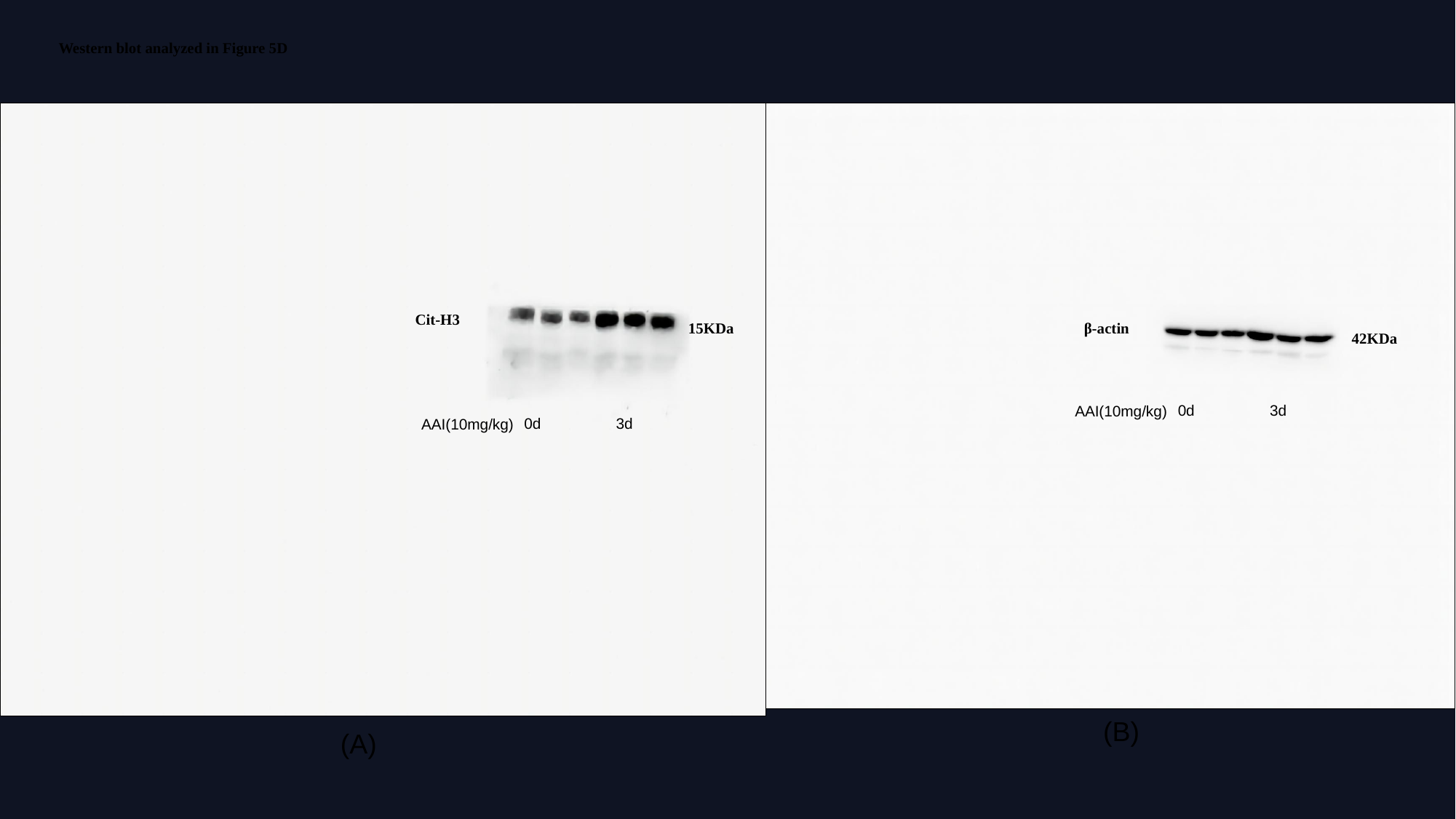

Western blot analyzed in Figure 5D
Cit-H3
15KDa
β-actin
42KDa
 0d 3d
 0d 3d
AAI(10mg/kg)
AAI(10mg/kg)
(B)
(A)

## Slide 2
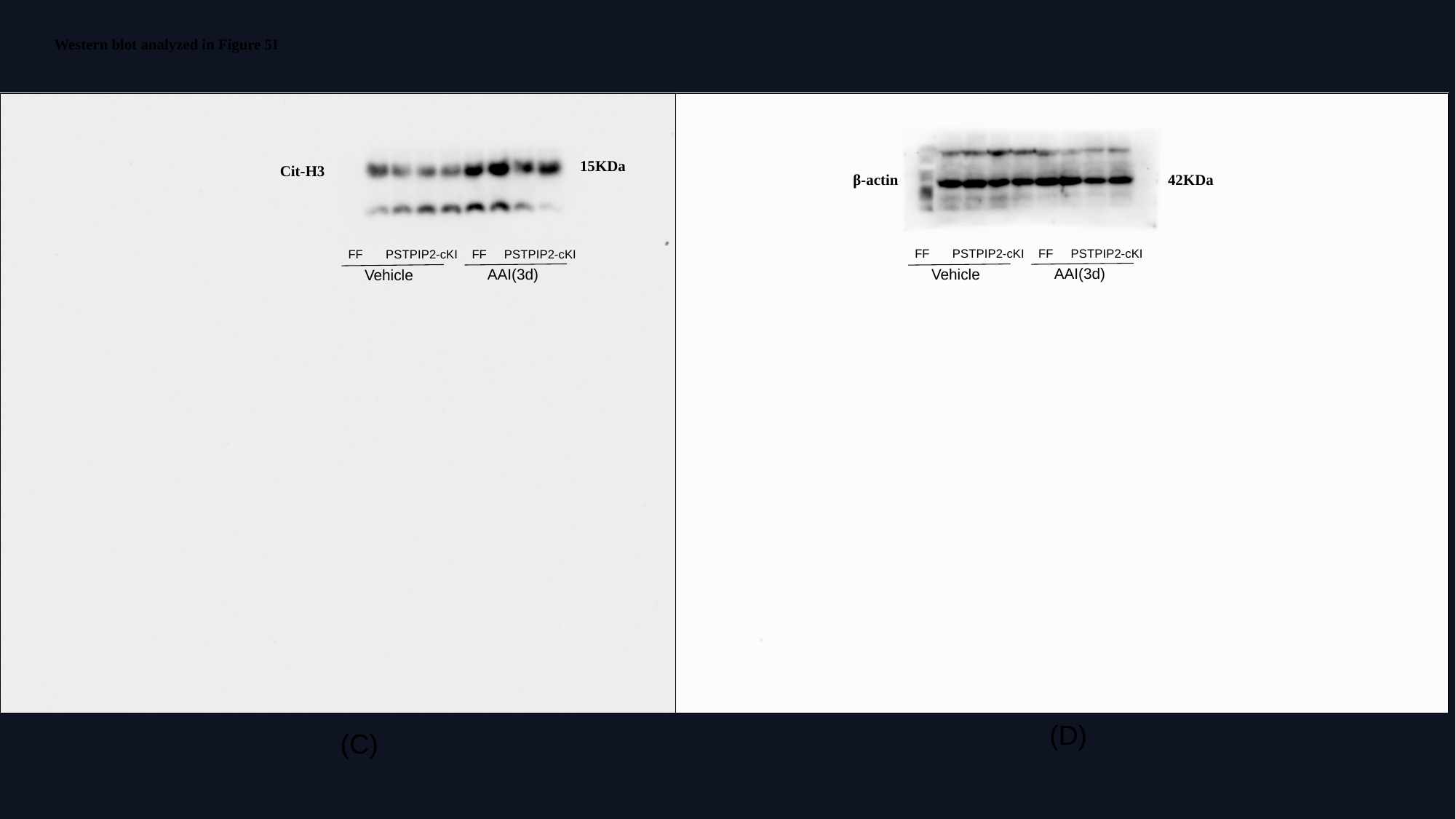

Western blot analyzed in Figure 5I
15KDa
Cit-H3
42KDa
β-actin
FF
PSTPIP2-cKI
FF
PSTPIP2-cKI
AAI(3d)
Vehicle
FF
PSTPIP2-cKI
FF
PSTPIP2-cKI
AAI(3d)
Vehicle
(D)
(C)
