# Supplementary material for: PSTPIP2 ameliorates aristolochic acid nephropathy by suppressing interleukin-19-mediated neutrophil extracellular trap formation
Source: eLife. 2024 Feb 5;13:e89740. doi: 10.7554/eLife.89740 (PMC10906995; doi:10.7554/eLife.89740)
Supplement: Figure 5—figure supplement 1—source data 2. [file elife-89740-fig5-figsupp1-data2.zip › Figure 5-figure supplement 1-data 2/Figure 5-figure supplement 1—source data 2.pptx]

## Slide 1
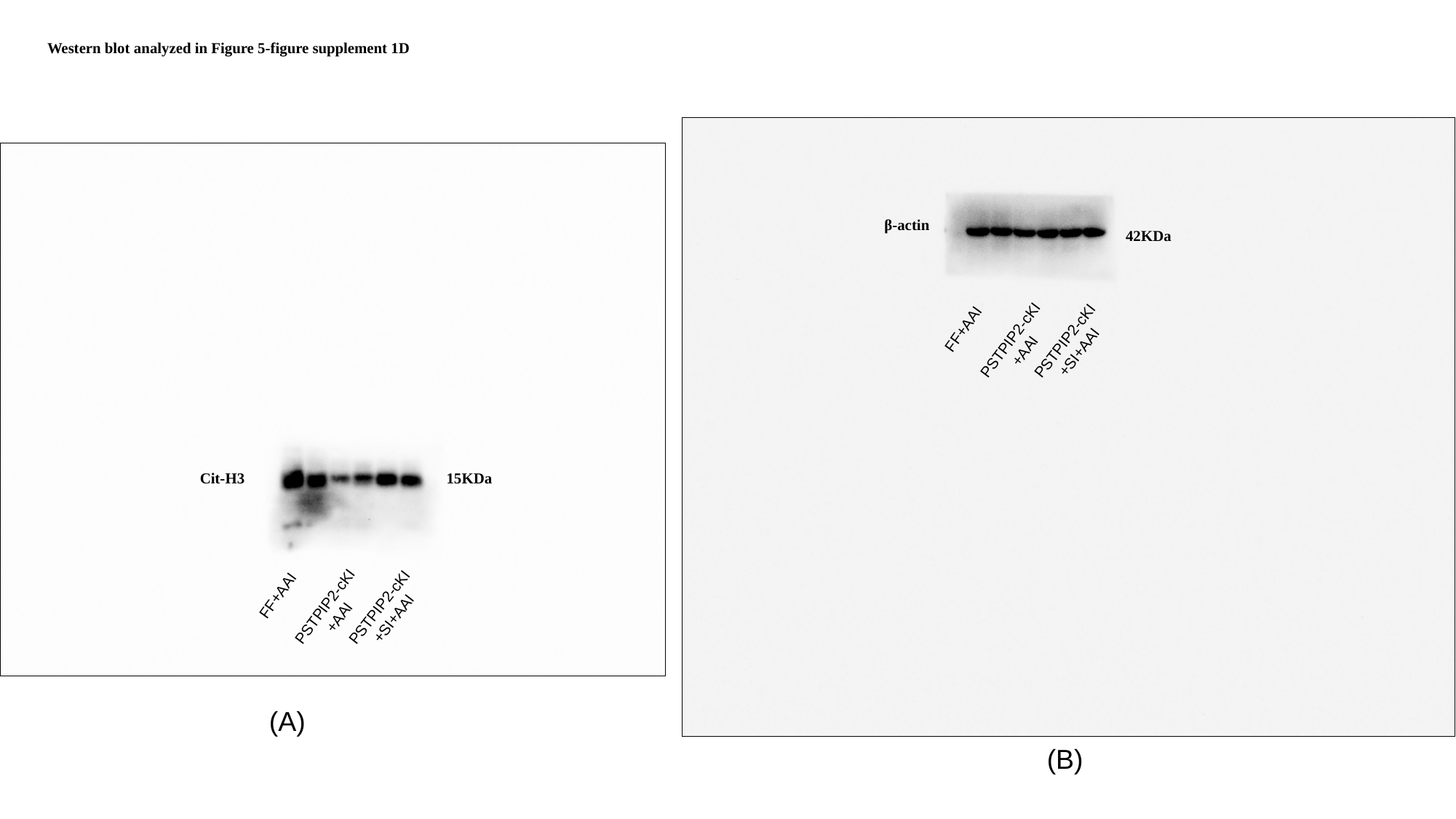

Western blot analyzed in Figure 5-figure supplement 1D
β-actin
42KDa
FF+AAI
PSTPIP2-cKI
+AAI
PSTPIP2-cKI
+SI+AAI
Cit-H3
15KDa
FF+AAI
PSTPIP2-cKI
+AAI
PSTPIP2-cKI
+SI+AAI
(A)
(B)
