# Supplementary material for: PSTPIP2 ameliorates aristolochic acid nephropathy by suppressing interleukin-19-mediated neutrophil extracellular trap formation
Source: eLife. 2024 Feb 5;13:e89740. doi: 10.7554/eLife.89740 (PMC10906995; doi:10.7554/eLife.89740)
Supplement: Figure 5—figure supplement 4—source data 2. [file elife-89740-fig5-figsupp4-data2.zip › Figure 5-figure supplement 4- data 2/Figure 5-figure supplement 4—source data 2.pptx]

## Slide 1
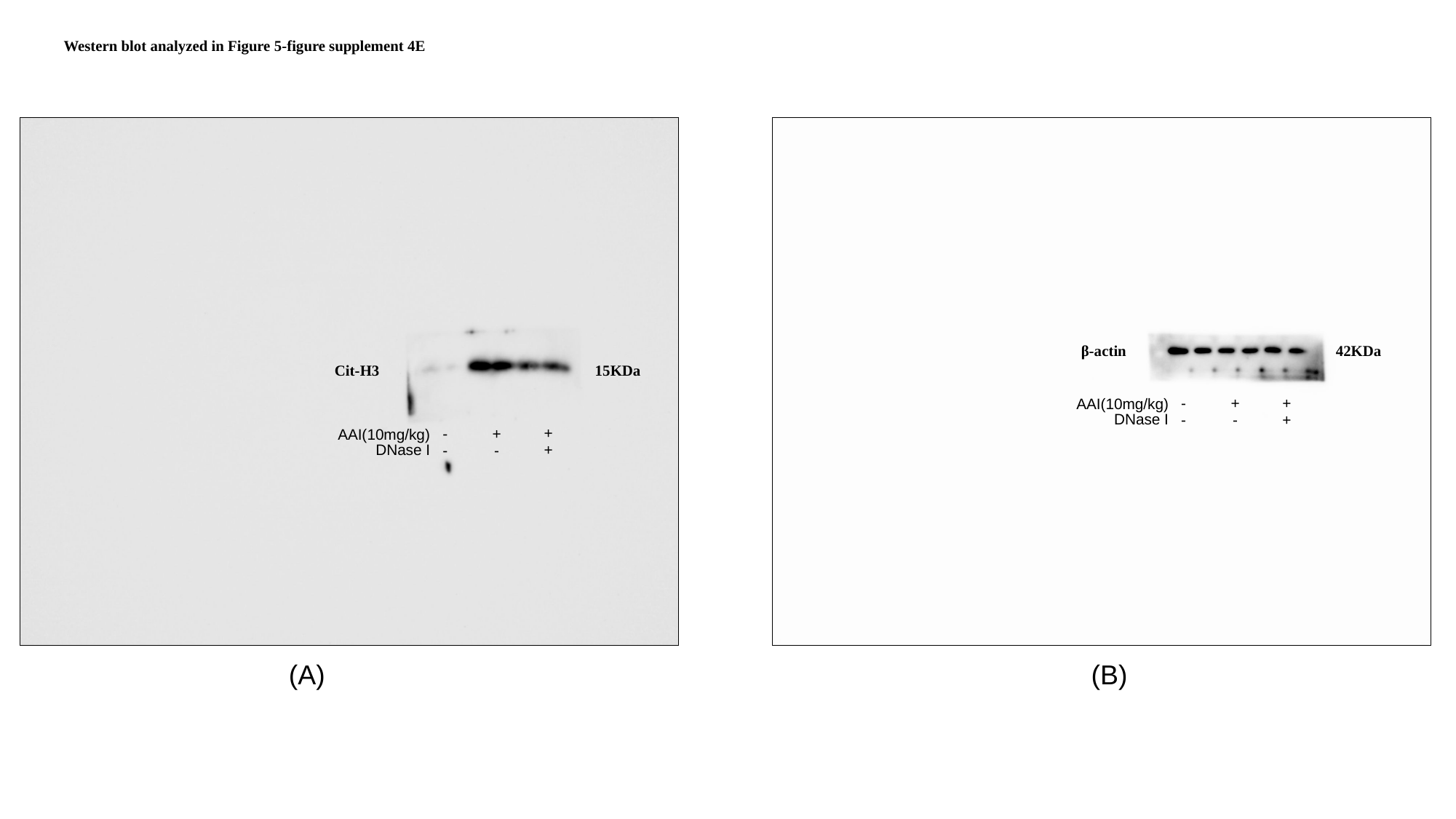

Western blot analyzed in Figure 5-figure supplement 4E
β-actin
42KDa
Cit-H3
15KDa
AAI(10mg/kg)
DNase I
+
+
-
-
+
-
AAI(10mg/kg)
DNase I
+
+
-
-
+
-
(A)
(B)

## Slide 2
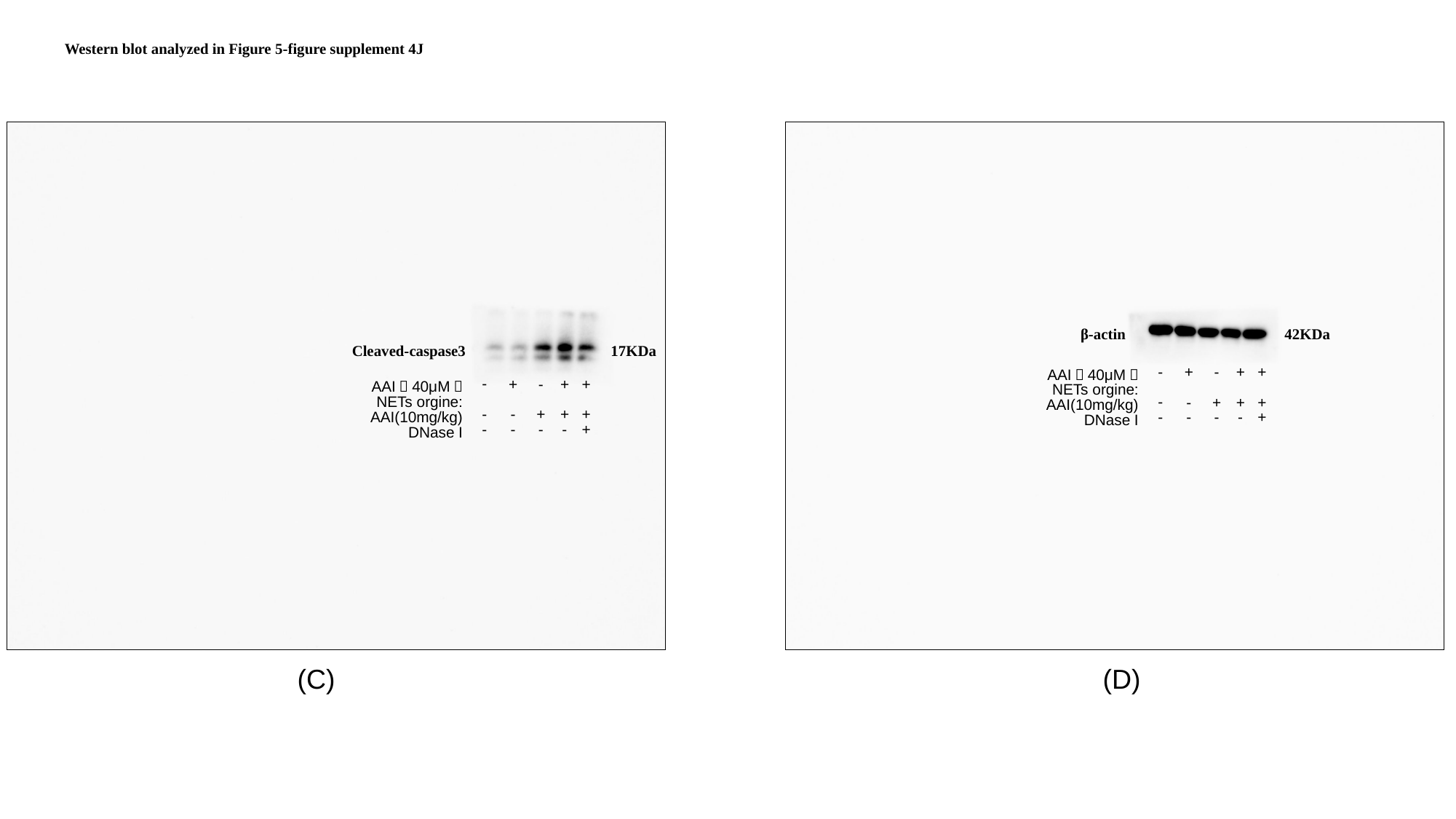

Western blot analyzed in Figure 5-figure supplement 4J
42KDa
β-actin
17KDa
Cleaved-caspase3
-
-
-
+
-
-
-
+
-
+
+
-
+
+
+
 AAI（40μM）
NETs orgine:
AAI(10mg/kg)
DNase I
-
-
-
+
-
-
-
+
-
+
+
-
+
+
+
 AAI（40μM）
NETs orgine:
AAI(10mg/kg)
DNase I
(C)
(D)
