# Supplementary material for: PSTPIP2 ameliorates aristolochic acid nephropathy by suppressing interleukin-19-mediated neutrophil extracellular trap formation
Source: eLife. 2024 Feb 5;13:e89740. doi: 10.7554/eLife.89740 (PMC10906995; doi:10.7554/eLife.89740)
Supplement: Figure 6—figure supplement 1—source data 2. [file elife-89740-fig6-figsupp1-data2.zip › Figure 6-figure supplement 1-data 2/Figure 6-figure supplement 1—source data 2.pptx]

## Slide 1
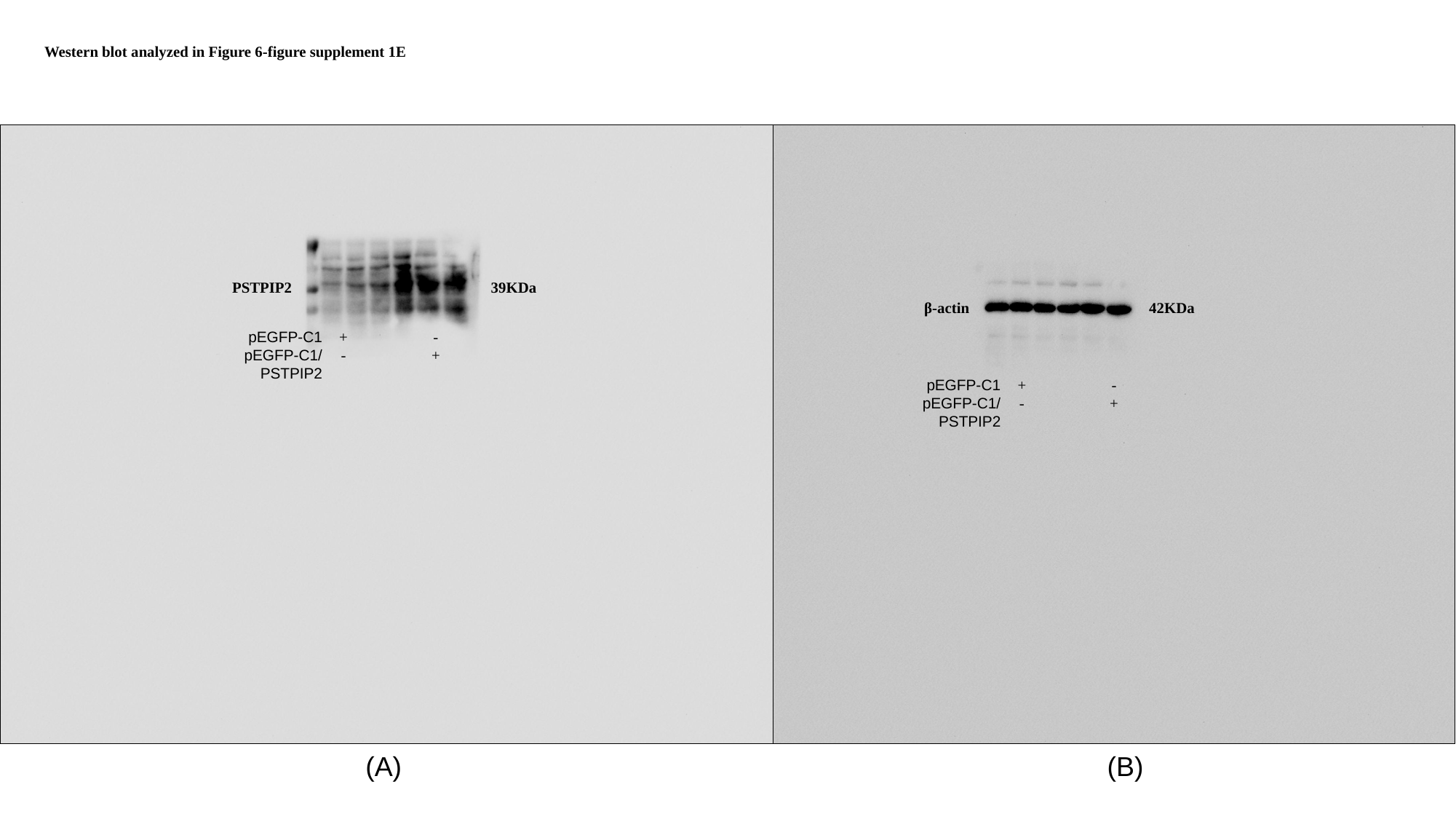

Western blot analyzed in Figure 6-figure supplement 1E
PSTPIP2
39KDa
β-actin
42KDa
pEGFP-C1
pEGFP-C1/PSTPIP2
+
-
-+
pEGFP-C1
pEGFP-C1/PSTPIP2
+
-
-+
(A)
(B)
