# Supplementary material for: PSTPIP2 ameliorates aristolochic acid nephropathy by suppressing interleukin-19-mediated neutrophil extracellular trap formation
Source: eLife. 2024 Feb 5;13:e89740. doi: 10.7554/eLife.89740 (PMC10906995; doi:10.7554/eLife.89740)
Supplement: Figure 7—source data 2. [file elife-89740-fig7-data2.zip › Figure 7-data 2/Figure 7-data 2.pptx]

## Slide 1
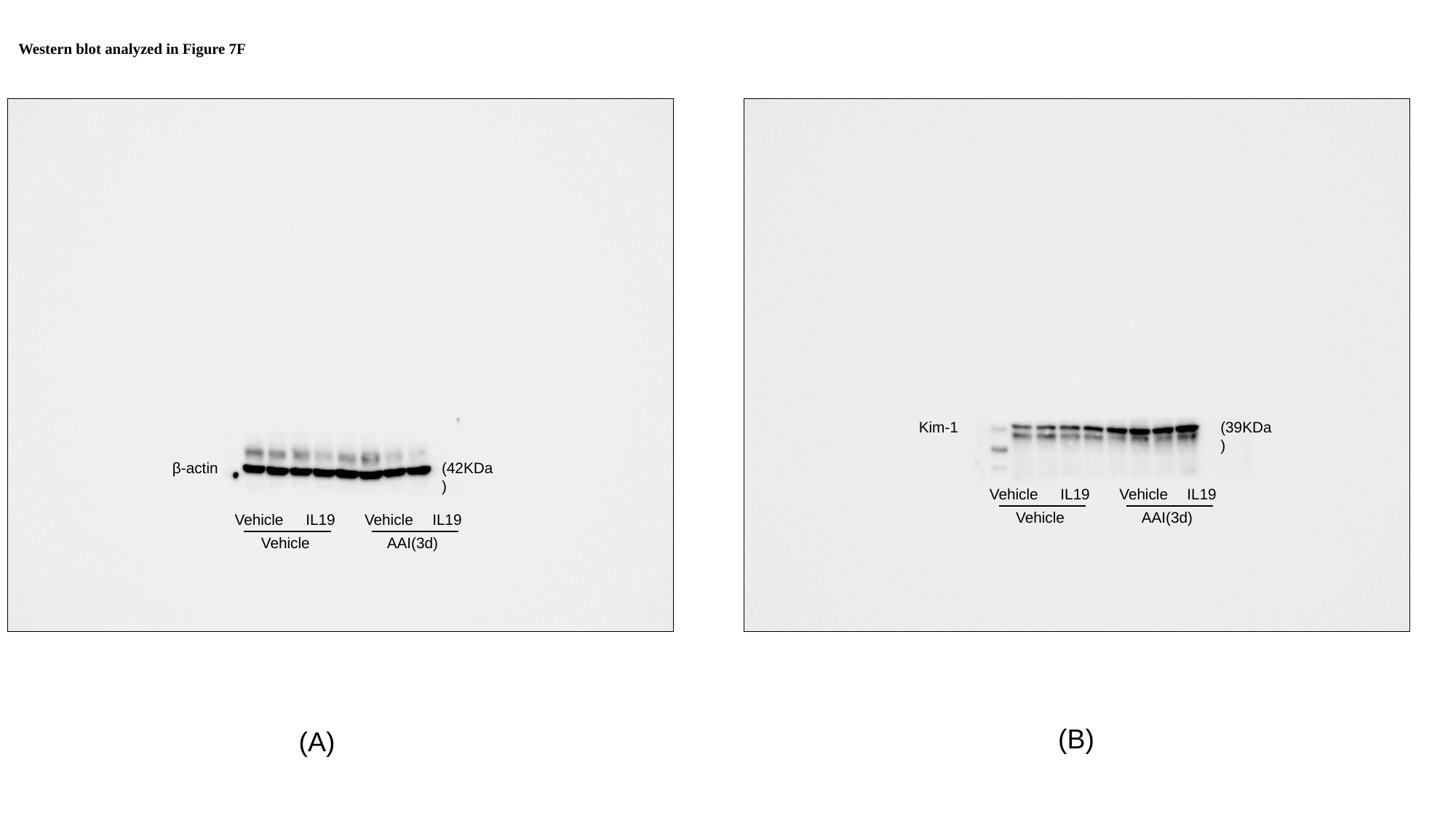

Western blot analyzed in Figure 7F
Kim-1
(39KDa)
β-actin
(42KDa)
Vehicle
IL19
Vehicle
IL19
Vehicle
AAI(3d)
Vehicle
IL19
Vehicle
IL19
Vehicle
AAI(3d)
(B)
(A)
