# Supplementary material for: PSTPIP2 ameliorates aristolochic acid nephropathy by suppressing interleukin-19-mediated neutrophil extracellular trap formation
Source: eLife. 2024 Feb 5;13:e89740. doi: 10.7554/eLife.89740 (PMC10906995; doi:10.7554/eLife.89740)
Supplement: Figure 8—source data 2. [file elife-89740-fig8-data2.zip › Figure 8-data 2/Figure 8-data 2.pptx]

## Slide 1
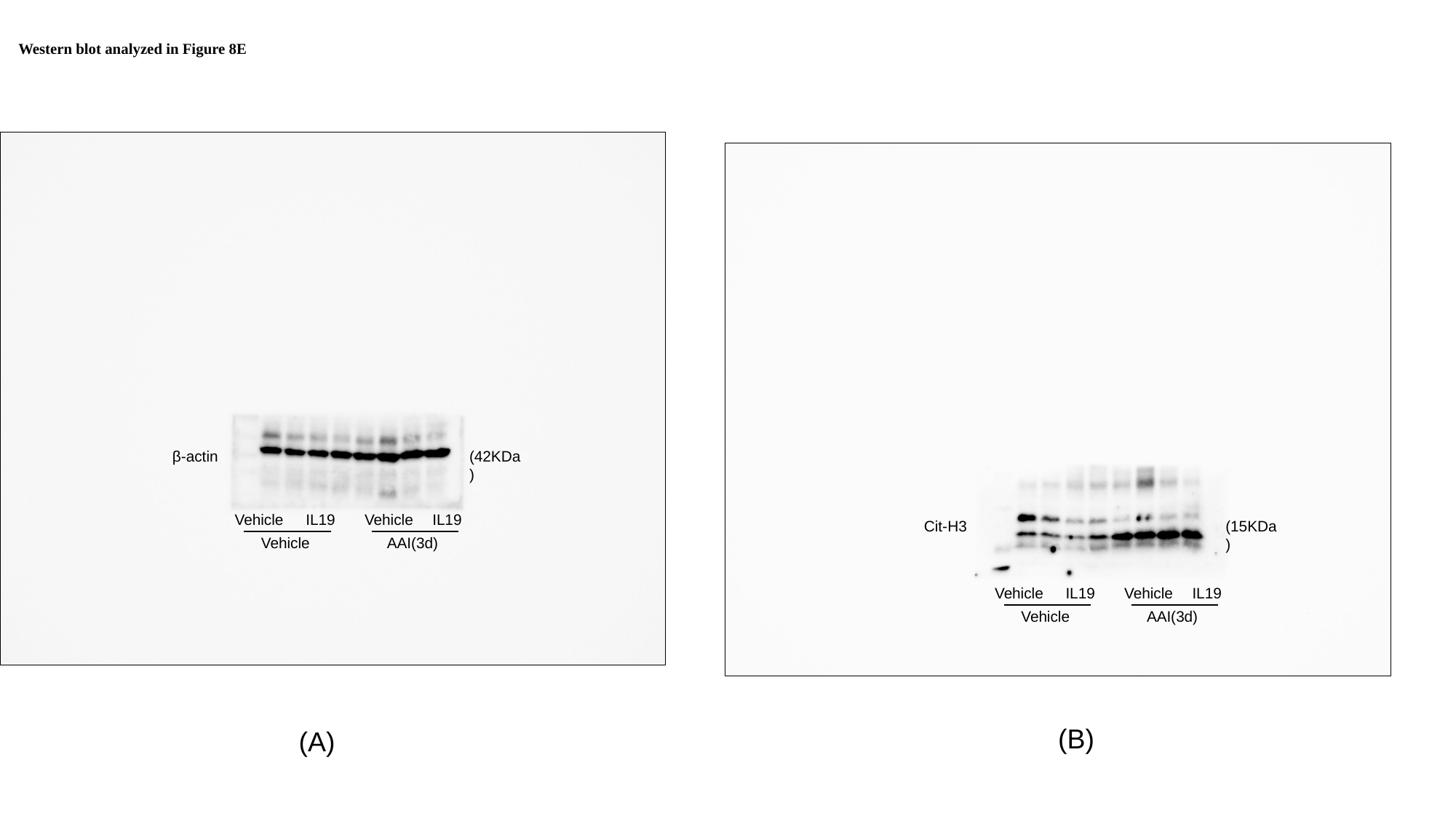

Western blot analyzed in Figure 8E
β-actin
(42KDa)
Vehicle
IL19
Vehicle
IL19
Vehicle
AAI(3d)
Cit-H3
(15KDa)
Vehicle
IL19
Vehicle
IL19
Vehicle
AAI(3d)
(B)
(A)
