# Supplementary material for: PSTPIP2 ameliorates aristolochic acid nephropathy by suppressing interleukin-19-mediated neutrophil extracellular trap formation
Source: eLife. 2024 Feb 5;13:e89740. doi: 10.7554/eLife.89740 (PMC10906995; doi:10.7554/eLife.89740)
Supplement: Figure 9—source data 2. [file elife-89740-fig9-data2.zip › Figure 9-data 2/Figure 9—source data 2.pptx]

## Slide 1
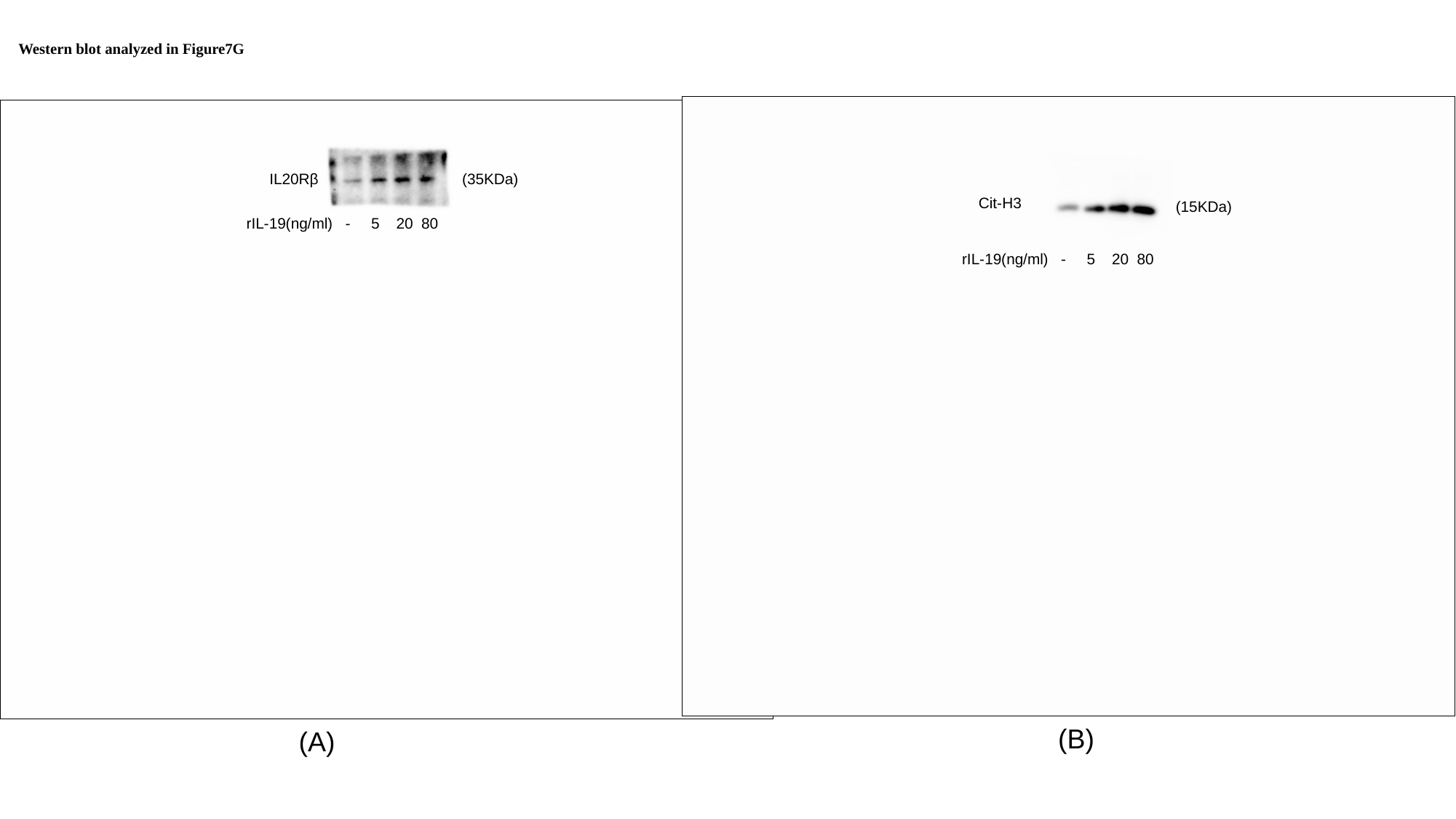

Western blot analyzed in Figure7G
IL20Rβ
(35KDa)
Cit-H3
(15KDa)
rIL-19(ng/ml) - 5 20 80
rIL-19(ng/ml) - 5 20 80
(B)
(A)

## Slide 2
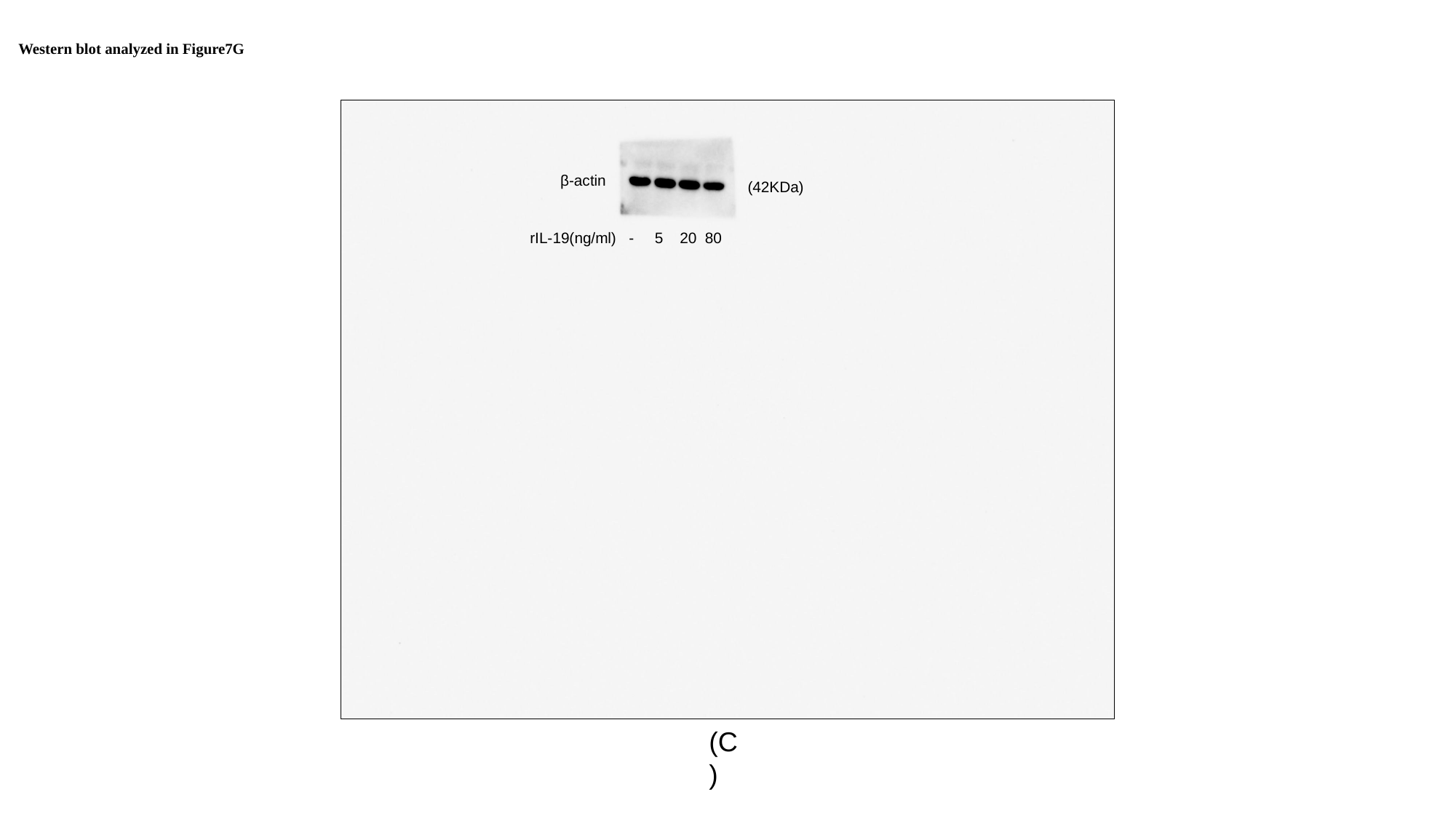

Western blot analyzed in Figure7G
β-actin
(42KDa)
rIL-19(ng/ml) - 5 20 80
(C)
