# Supplementary material for: PSTPIP2 ameliorates aristolochic acid nephropathy by suppressing interleukin-19-mediated neutrophil extracellular trap formation
Source: eLife. 2024 Feb 5;13:e89740. doi: 10.7554/eLife.89740 (PMC10906995; doi:10.7554/eLife.89740)
Supplement: Figure 9—figure supplement 1—source data 2. [file elife-89740-fig9-figsupp1-data2.zip › Figure 9-figure supplement 1-data 2/Figure 9-figure supplement 1—source data 2.pptx]

## Slide 1
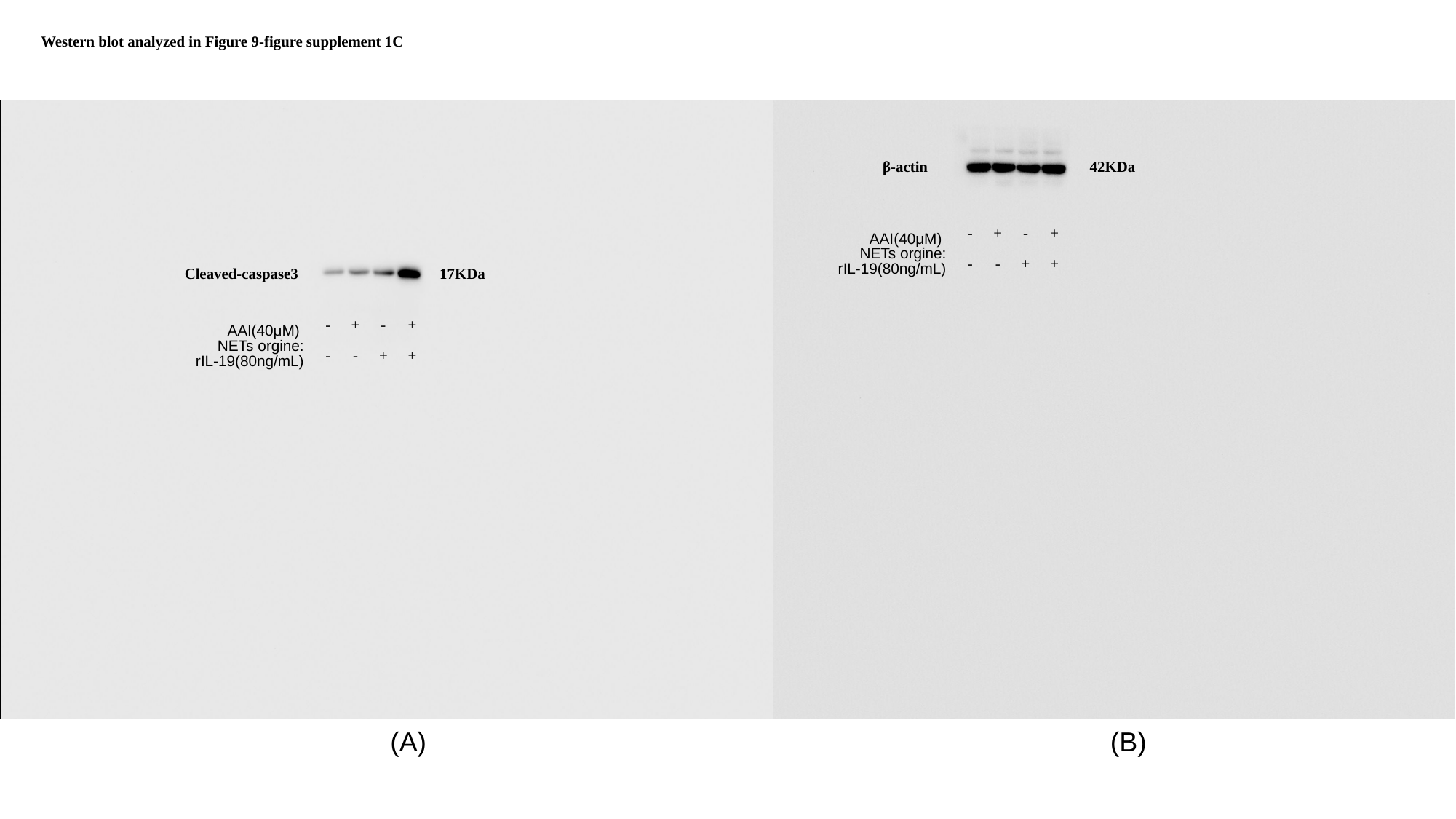

Western blot analyzed in Figure 9-figure supplement 1C
β-actin
42KDa
-
-
+
-
-
+
+
+
 AAI(40μM)
NETs orgine:
rIL-19(80ng/mL)
Cleaved-caspase3
17KDa
-
-
+
-
-
+
+
+
 AAI(40μM)
NETs orgine:
rIL-19(80ng/mL)
(A)
(B)
