# Supplementary material for: PSTPIP2 ameliorates aristolochic acid nephropathy by suppressing interleukin-19-mediated neutrophil extracellular trap formation
Source: eLife. 2024 Feb 5;13:e89740. doi: 10.7554/eLife.89740 (PMC10906995; doi:10.7554/eLife.89740)
Supplement: Figure 10—source data 2. [file elife-89740-fig10-data2.zip › Figure 10-data2/Figure 10—source data 2.pptx]

## Slide 1
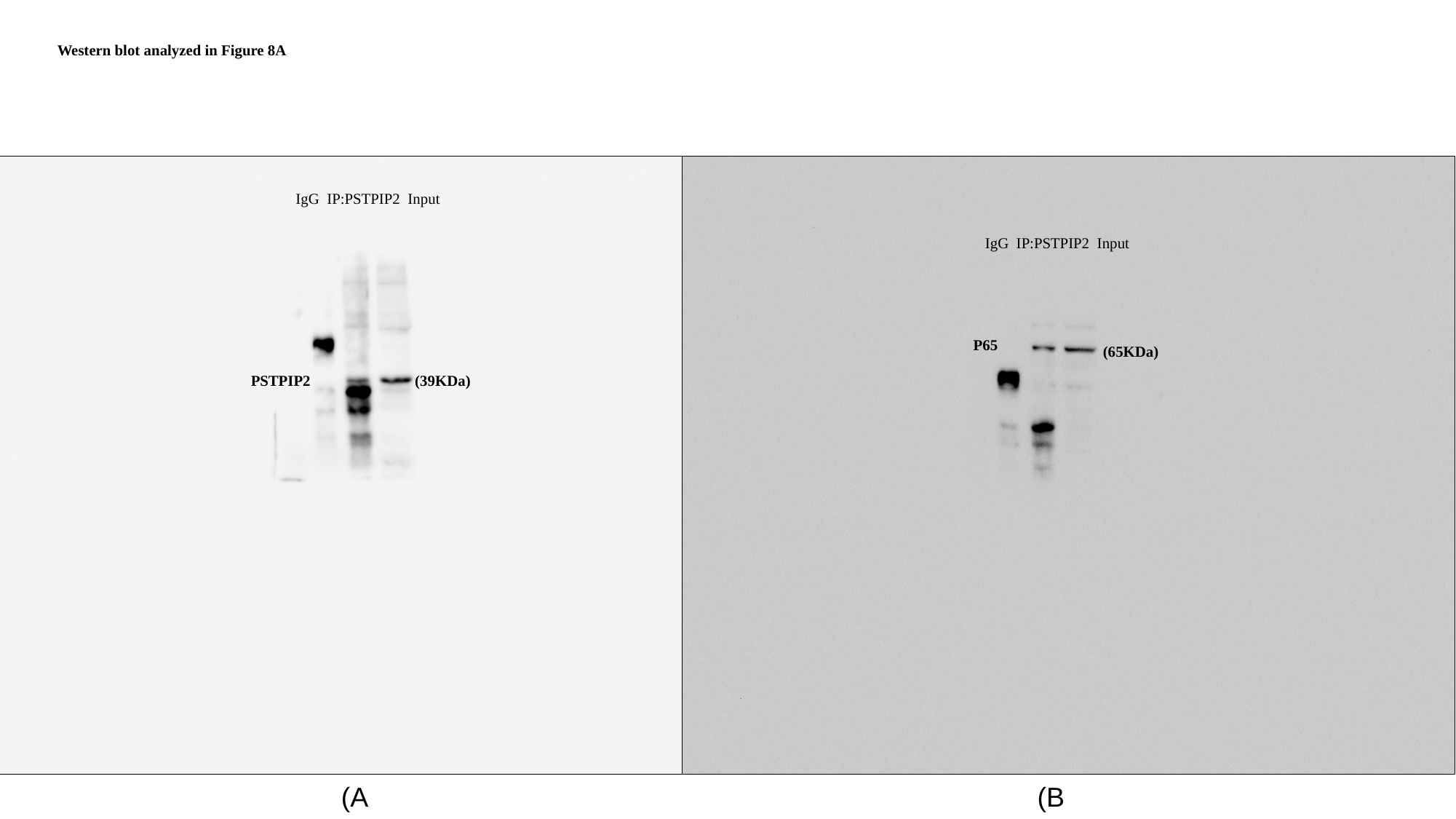

Western blot analyzed in Figure 8A
 IgG IP:PSTPIP2 Input
 IgG IP:PSTPIP2 Input
P65
(65KDa)
PSTPIP2
(39KDa)
(A)
(B)

## Slide 2
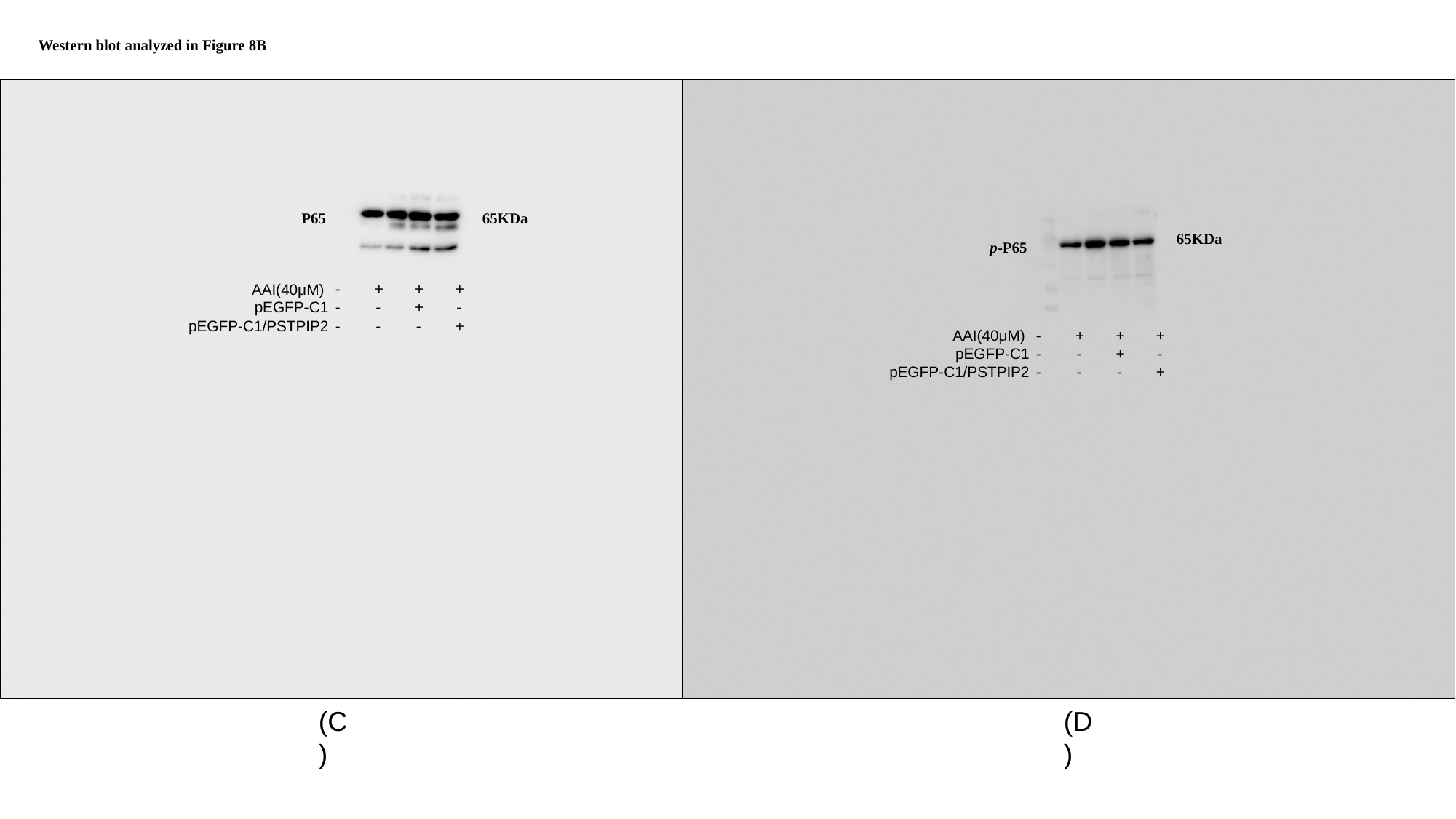

Western blot analyzed in Figure 8B
P65
65KDa
65KDa
p-P65
-
-
-
+-
-
++
-
+
-+
AAI(40μM)
pEGFP-C1
pEGFP-C1/PSTPIP2
-
-
-
+-
-
++
-
+
-+
AAI(40μM)
pEGFP-C1
pEGFP-C1/PSTPIP2
(C)
(D)

## Slide 3
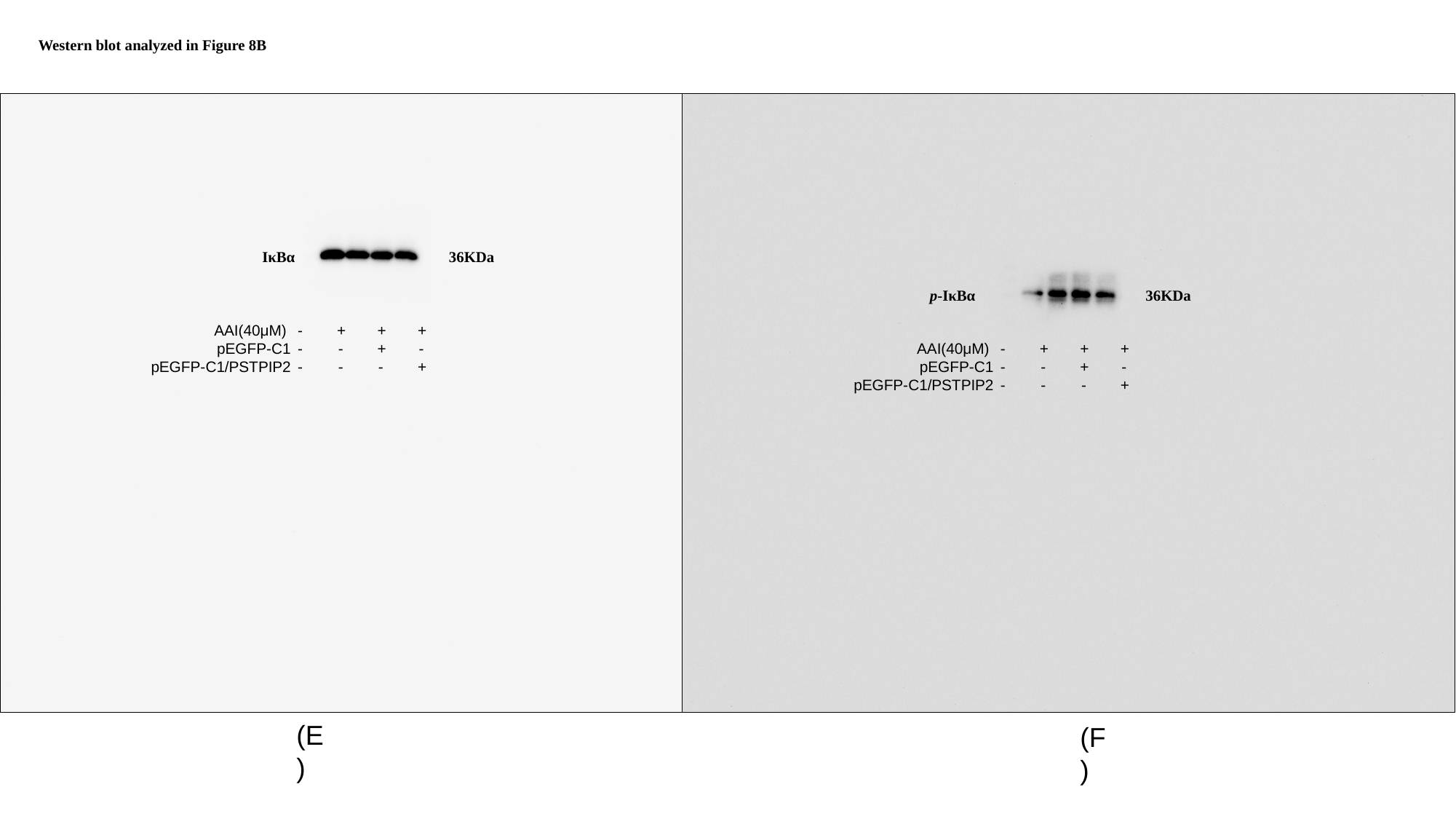

Western blot analyzed in Figure 8B
36KDa
IκBα
36KDa
p-IκBα
-
-
-
+-
-
++
-
+
-+
AAI(40μM)
pEGFP-C1
pEGFP-C1/PSTPIP2
-
-
-
+-
-
++
-
+
-+
AAI(40μM)
pEGFP-C1
pEGFP-C1/PSTPIP2
(E)
(F)

## Slide 4
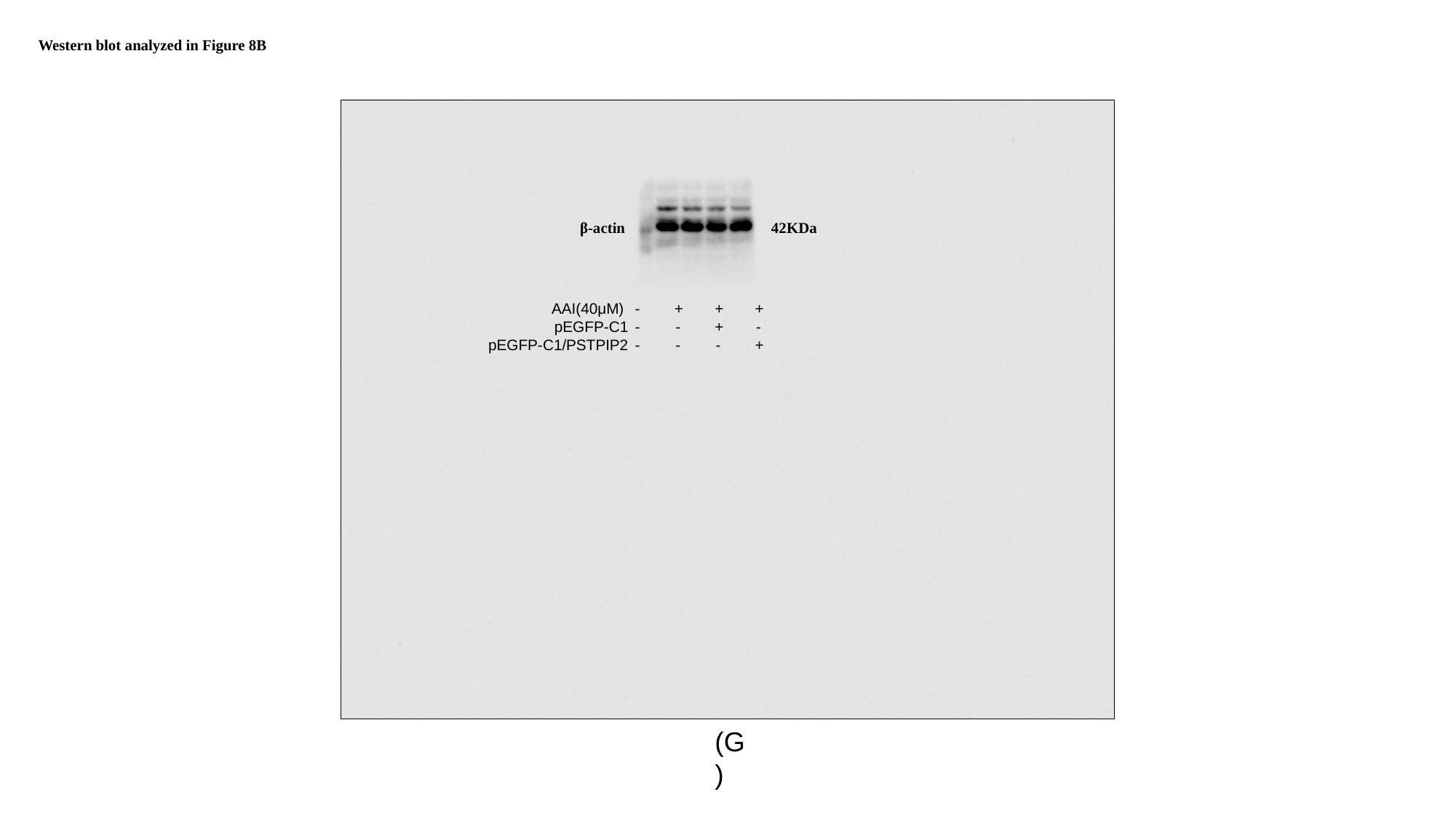

Western blot analyzed in Figure 8B
β-actin
42KDa
-
-
-
+-
-
++
-
+
-+
AAI(40μM)
pEGFP-C1
pEGFP-C1/PSTPIP2
(G)

## Slide 5
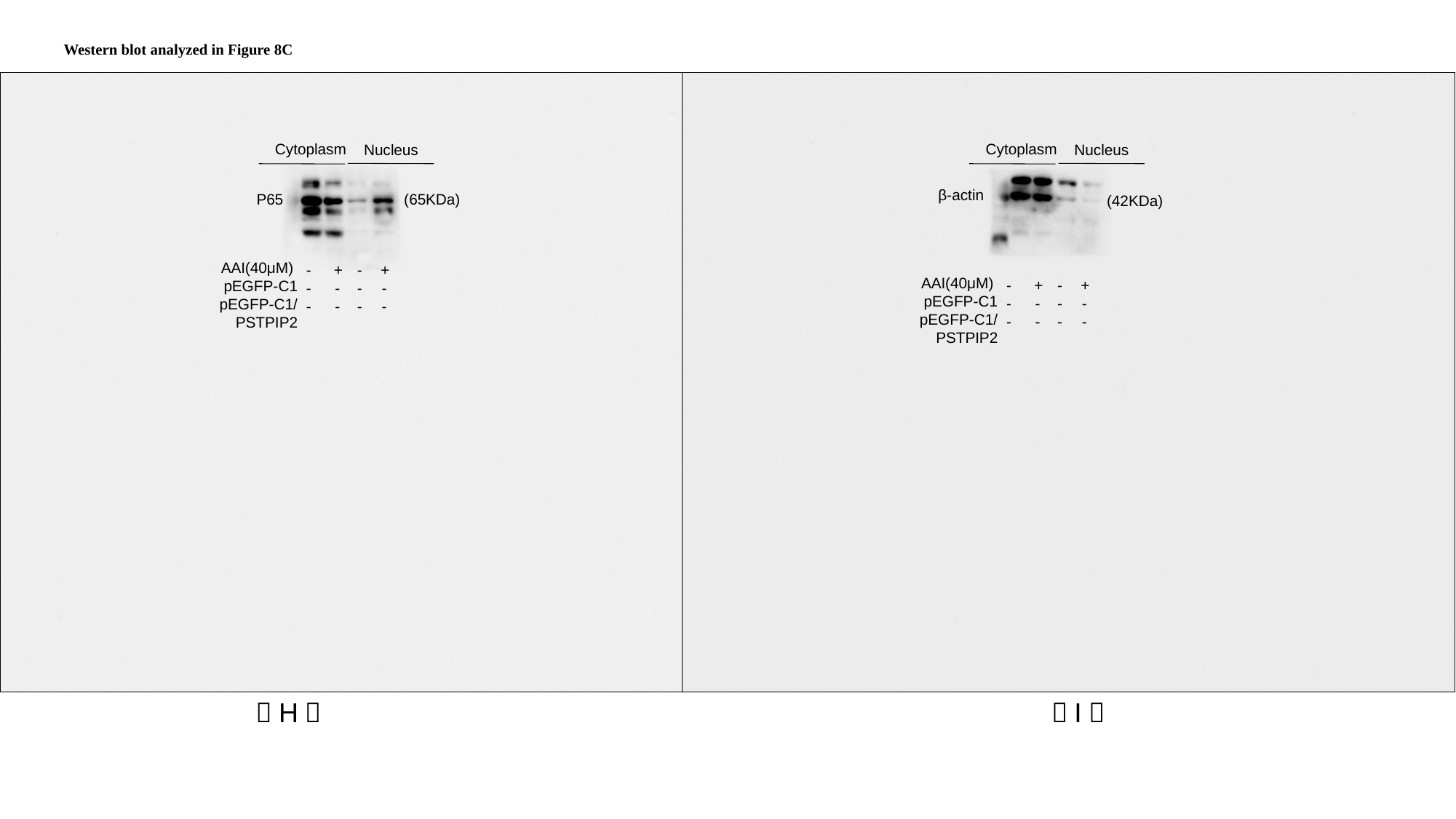

Western blot analyzed in Figure 8C
Cytoplasm
Nucleus
Cytoplasm
Nucleus
β-actin
P65
(65KDa)
(42KDa)
-
-
-
+-
-
-
-
-
+-
-
AAI(40μM)
pEGFP-C1
pEGFP-C1/
PSTPIP2
-
-
-
+-
-
-
-
-
+-
-
AAI(40μM)
pEGFP-C1
pEGFP-C1/
PSTPIP2
（H）
（I）

## Slide 6
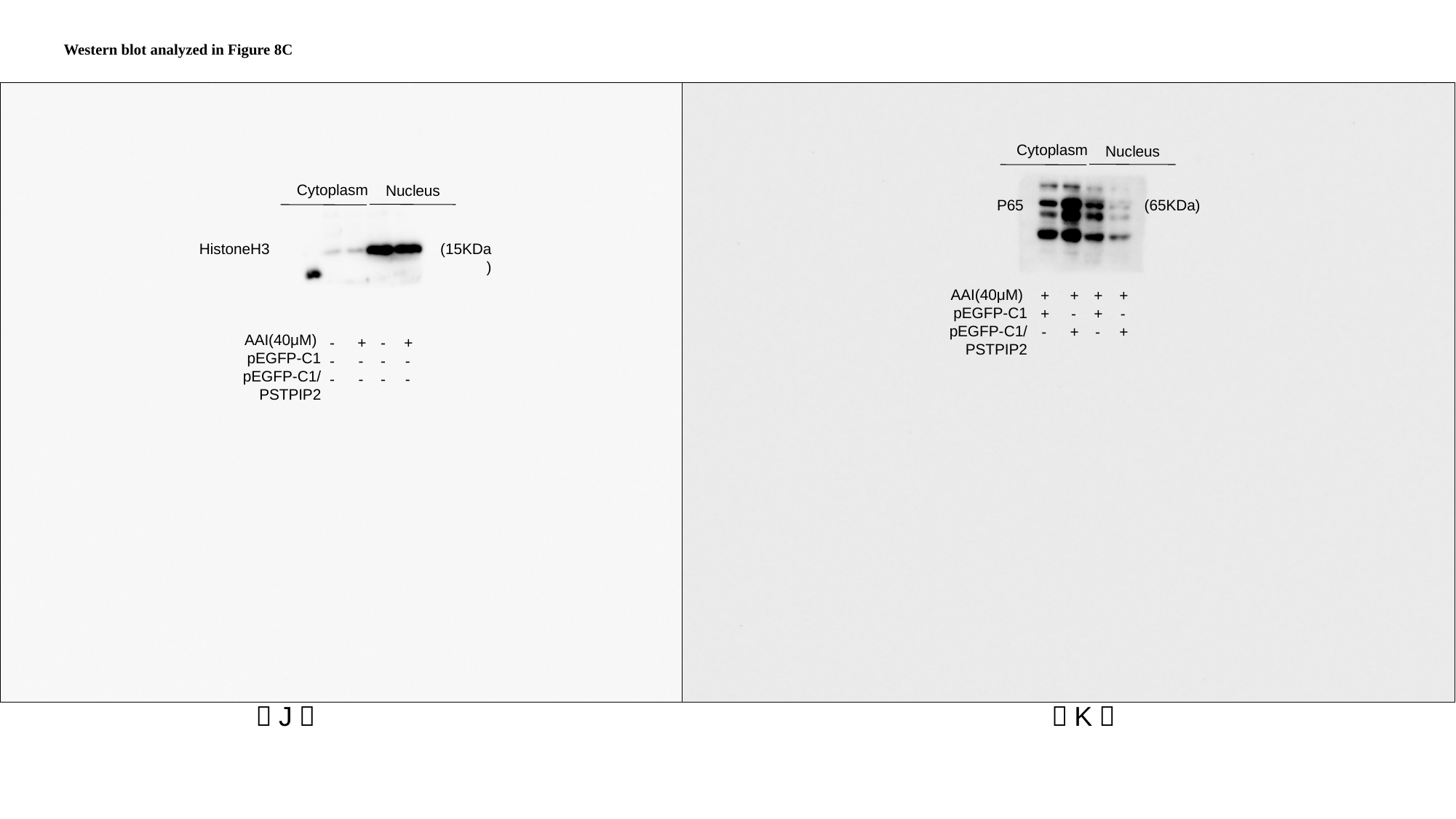

Western blot analyzed in Figure 8C
Cytoplasm
Nucleus
Cytoplasm
Nucleus
P65
(65KDa)
HistoneH3
(15KDa)
++
-
+
-+
++
-
+
-+
AAI(40μM)
pEGFP-C1
pEGFP-C1/
PSTPIP2
-
-
-
+-
-
-
-
-
+-
-
AAI(40μM)
pEGFP-C1
pEGFP-C1/
PSTPIP2
（J）
（K）

## Slide 7
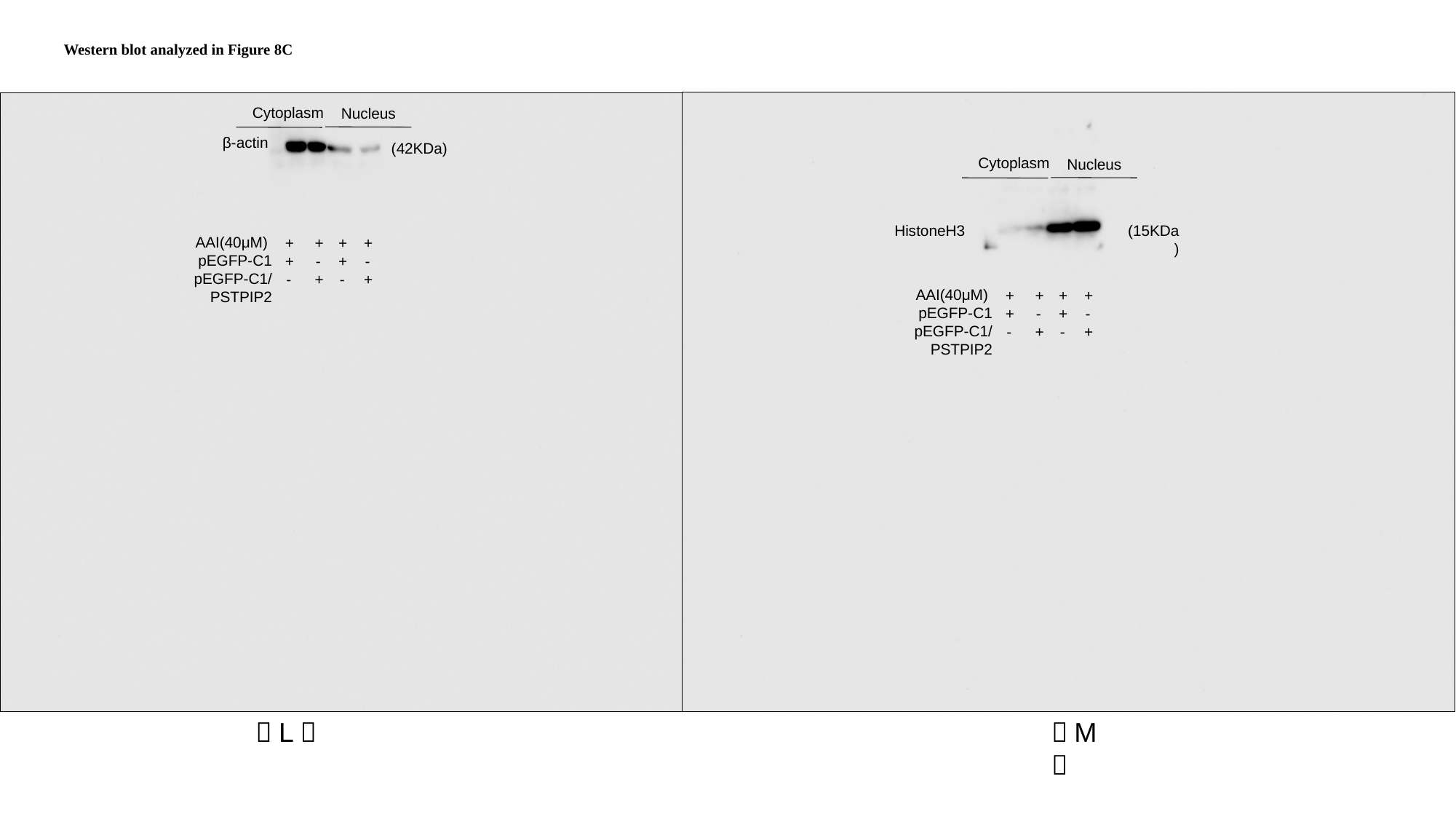

Western blot analyzed in Figure 8C
Cytoplasm
Nucleus
β-actin
(42KDa)
Cytoplasm
Nucleus
HistoneH3
(15KDa)
++
-
+
-+
++
-
+
-+
AAI(40μM)
pEGFP-C1
pEGFP-C1/
PSTPIP2
++
-
+
-+
++
-
+
-+
AAI(40μM)
pEGFP-C1
pEGFP-C1/
PSTPIP2
（L）
（M）

## Slide 8
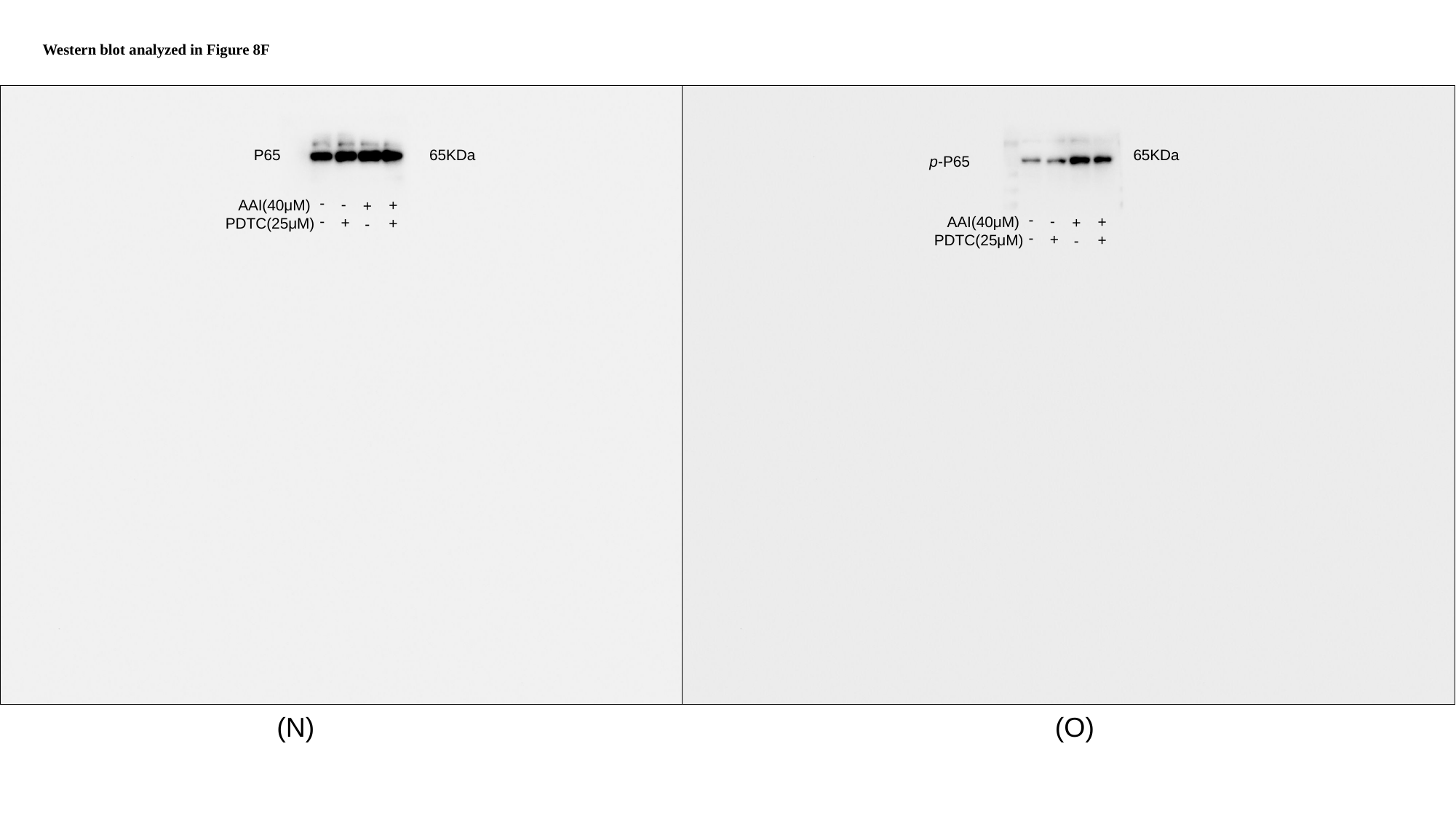

Western blot analyzed in Figure 8F
P65
65KDa
65KDa
p-P65
-
-
-
+
+
+
+
-
AAI(40μM)
PDTC(25μM)
-
-
-
+
+
+
+
-
AAI(40μM)
PDTC(25μM)
(N)
(O)

## Slide 9
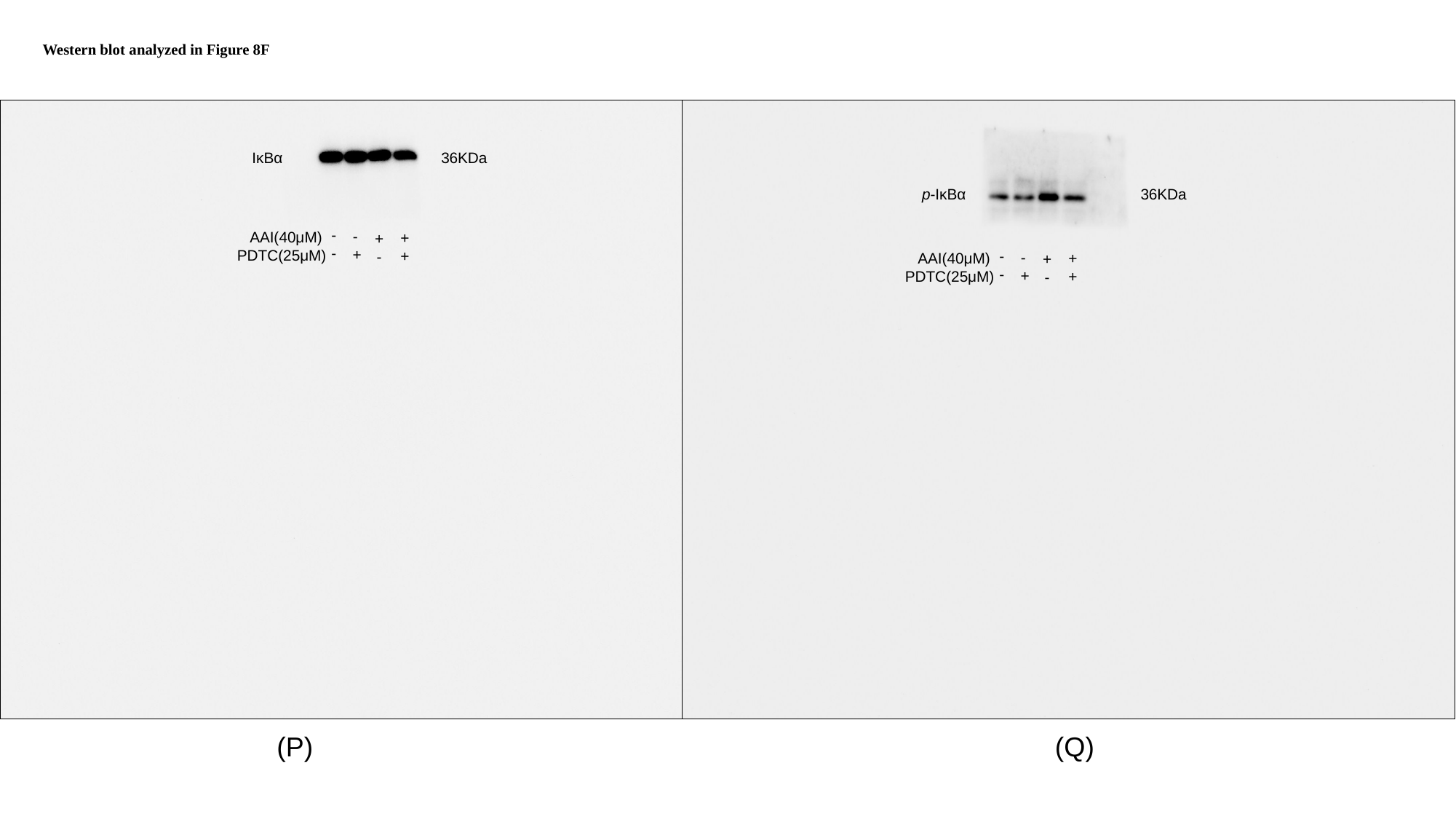

Western blot analyzed in Figure 8F
IκBα
36KDa
p-IκBα
36KDa
-
-
-
+
+
+
+
-
AAI(40μM)
PDTC(25μM)
-
-
-
+
+
+
+
-
AAI(40μM)
PDTC(25μM)
(P)
(Q)

## Slide 10
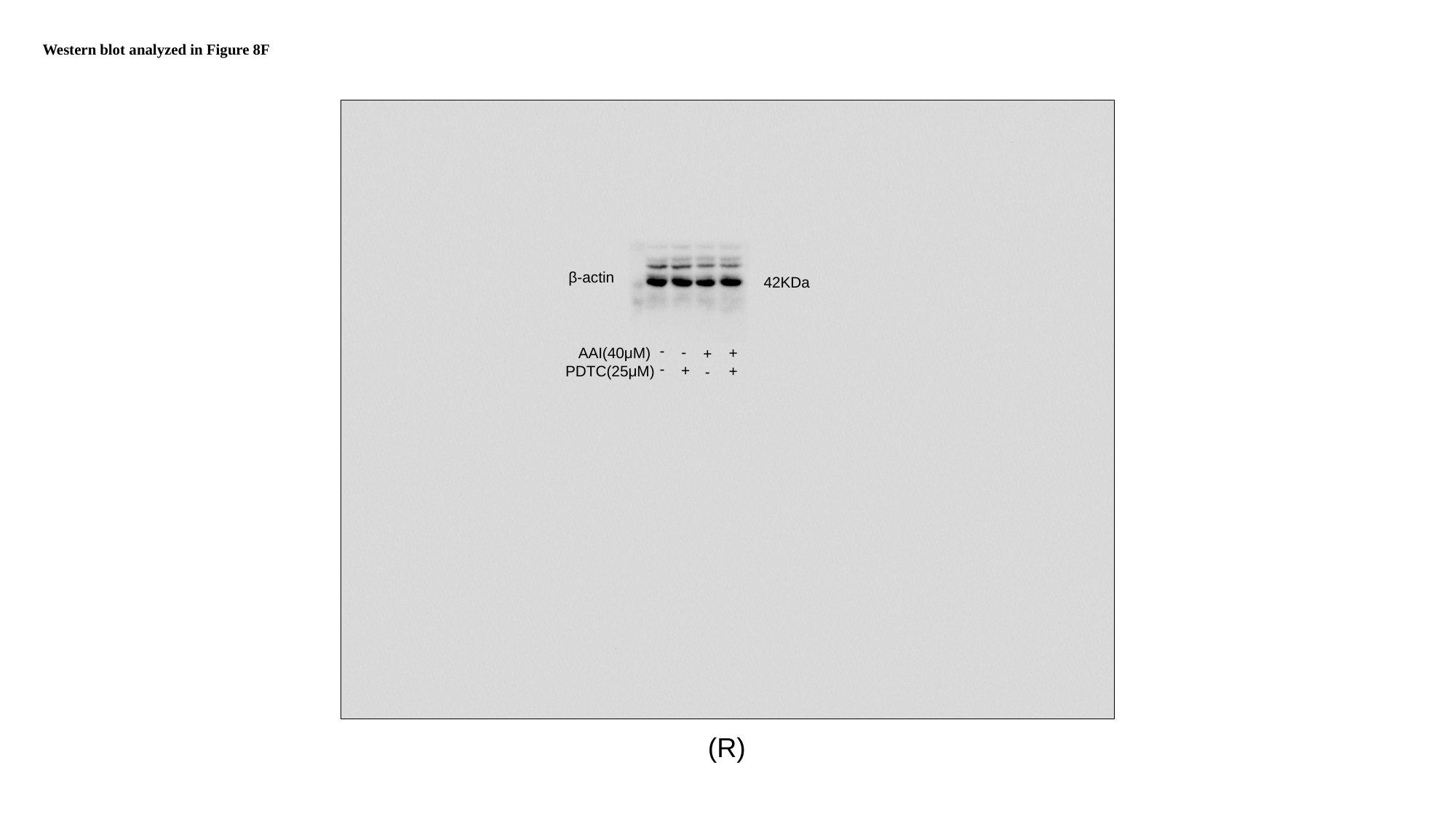

Western blot analyzed in Figure 8F
β-actin
42KDa
-
-
-
+
+
+
+
-
AAI(40μM)
PDTC(25μM)
(R)
